# Digital Biomarkers for Parkinson Disease: Bibliometric Analysis and a Scoping Review of Deep Learning for Freezing of Gait

**DOI:** 10.2196/71560

**Published:** 2025-05-20

**Authors:** Wenhao Qi, Shiying Shen, Chaoqun dong, Mengjiao Zhao, Shuaiqi Zang, Xiaohong Zhu, Jiaqi Li, Bin Wang, Yankai Shi, Yongze Dong, Huajuan Shen, Junling Kang, Xiaodong Lu, Guowei Jiang, Jingsong Du, Eryi Shu, Qingbo Zhou, Jinghua Wang, Shihua Cao

**Affiliations:** 1 School of Nursing Hangzhou Normal University Hangzhou China; 2 Department of Neurology Affiliated Hospital of Hangzhou Normal University Hangzhou China; 3 Zhejiang Key Laboratory for Research in Assessment of Cognitive Impairments Hangzhou China; 4 School of Nursing Zhejiang Chinese Medical University Hangzhou China; 5 Nursing Department Zhejiang Provincial People's Hospital Hangzhou China; 6 Department of Neurology The Third Affiliated Hospital of Zhejiang Chinese Medical University Hangzhou China; 7 Department of Psychiatry and Neuropsychology and Alzheimer Centrum Limburg School for Mental Health and Neuroscience Maastricht University Maastricht The Netherlands; 8 School of Health Management Zaozhuang University Zaozhuang China; 9 Zhejiang Medical & Health Group Hangzhou Hospital Hangzhou China; 10 Department of Geriatrics The Second Hospital, Cheeloo College of Medicine Shandong University Shandong China; 11 School of Nursing and Rehabilitation Cheeloo College of Medicine Shandong University Shandong China; 12 Engineering Research Center of Mobile Health Management System Ministry of Education Hangzhou China

**Keywords:** Parkinson disease, digital biomarker, artificial intelligence, AI, machine learning, deep learning, bibliometric analysis, PRISMA

## Abstract

**Background:**

With the rapid development of digital biomarkers in Parkinson disease (PD) research, it has become increasingly important to explore the current research trends and key areas of focus.

**Objective:**

This study aimed to comprehensively evaluate the current status, hot spots, and future trends of global PD biomarker research, and provide a systematic review of deep learning models for freezing of gait (FOG) digital biomarkers.

**Methods:**

This study used bibliometric analysis based on the Web of Science Core Collection database to conduct a comprehensive analysis of the multidimensional landscape of Parkinson digital biomarkers. After identifying research hot spots, the study also followed the PRISMA-ScR (Preferred Reporting Items for Systematic Reviews and Meta-Analyses Extension for Scoping Reviews) guidelines for a scoping review of deep learning models for FOG from 5 databases: Web of Science, PubMed, IEEE Xplore, Embase, and Google Scholar.

**Results:**

A total of 750 studies were included in the bibliometric analysis, and 40 studies were included in the scoping review. The analysis revealed a growing number of related publications, with 3700 researchers contributing. Neurology had the highest average annual participation rate (12.46/19, 66%). The United States contributed the most research (192/1171, 16.4%), with 210 participating institutions, which was the highest among all countries. In the study of deep learning models for FOG, the average accuracy of the models was 0.92, sensitivity was 0.88, specificity was 0.90, and area under the curve was 0.91. In addition, 31 (78%) studies indicated that the best models were primarily convolutional neural networks or convolutional neural networks–based architectures.

**Conclusions:**

Research on digital biomarkers for PD is currently at a stable stage of development, with widespread global interest from countries, institutions, and researchers. However, challenges remain, including insufficient interdisciplinary and interinstitutional collaboration, as well as a lack of corporate funding for related projects. Current research trends primarily focus on motor-related studies, particularly FOG monitoring. However, deep learning models for FOG still lack external validation and standardized performance reporting. Future research will likely progress toward deeper applications of artificial intelligence, enhanced interinstitutional collaboration, comprehensive analysis of different data types, and the exploration of digital biomarkers for a broader range of Parkinson symptoms.

**Trial Registration:**

Open Science Foundation (OSF Registries) OSF.IO/RG8Y3; https://doi.org/10.17605/OSF.IO/RG8Y3

## Introduction

### Background

Parkinson disease (PD) is the second most common neurodegenerative disorder, following Alzheimer disease, primarily caused by the selective loss of dopaminergic neurons in the substantia nigra of the midbrain [1]. The global prevalence of PD exceeds 6 million, a figure that has increased 2.5 fold compared to previous generations, with no effective cure available to date [2]. As such, PD has become one of the leading causes of neurogenic disability [3]. The symptoms of PD include both motor and nonmotor impairments. Motor symptoms consist of resting tremor, rigidity, bradykinesia, and postural instability [4,5], while nonmotor symptoms encompass cognitive dysfunction, olfactory dysfunction, mood disorders, sleep disturbances, and autonomic dysfunction [6,7]. These symptoms not only significantly reduce the quality of life for patients but also impose heavy economic and caregiving burdens on families and society [8]. Consequently, the development of more efficient and precise diagnostic methods and disease management strategies has become an urgent research priority [9].

Currently, the diagnosis of PD mainly relies on the identification of clinical symptoms and signs, including 3 core motor symptoms: bradykinesia and the presence of either resting tremor or rigidity [10]. The diagnostic process should follow the methodology described in the Movement Disorder Society-Unified Parkinson’s Disease Rating Scale [11]. Historically, pathological evidence of Lewy bodies found in autopsy studies was considered the gold standard for diagnosis [12]. Imaging techniques, such as brain magnetic resonance imaging [13], genetic testing, and biomarker studies (eg, α-synuclein and leucine-rich repeat kinase 2 gene mutations) [14,15], can provide key diagnostic information, but these methods are mainly used for research and as adjunctive diagnostic tools. They are costly and difficult to repeat. With the advancement of mobile digital technologies, digital devices are now able to more accurately track changes in patient symptoms, especially those subtle changes that are difficult to detect in traditional clinical assessments.

Freezing of gait (FOG) is a manifestation of motor dysfunction in PD that affects approximately 80% of patients in the middle to advanced stages. It is highly disabling as it reduces mobility and frequently leads to falls and fall-related injuries [16]. Clinical assessment relies on the new FOG questionnaire and visual observation [17], both of which present challenges related to reliability and subjectivity. With the advancement of wireless communication and microelectronics, wearable devices have become smaller, lighter, and more cost-effective [18]. Furthermore, automated detection offers the advantage of saving time and costs associated with expert reviews and postevent video analysis, enabling broader testing and enhancing real-time interventions. Therefore, the use of wearable sensors combined with statistical methods for the automatic detection and prediction of FOG has emerged as a promising tool.

For the complex neurodegenerative disorder of PD, digital biomarkers are gradually becoming an important tool to drive the transition from traditional medicine to precision medicine [19,20]. Specifically, the US Food and Drug Administration (FDA) defines digital biomarkers as “characteristics or a set of characteristics collected through digital health technologies, which are used to indicate normal biological processes, pathological processes, or biological responses to an exposure or intervention, including therapeutic interventions” [21]. In PD research, digital biomarkers typically refer to objective data related to health, collected and analyzed through digital devices (such as smartphones, wearables, and sensors) to characterize health status or disease progression [22]. Compared to traditional assessment methods that rely on subjective scales [23] or patient diaries [24], digital biomarkers offer significant advantages in real time, objectivity, and high sensitivity [25]. For instance, wearable devices allow continuous, unobtrusive data collection in patients’ everyday environments, capturing subtle behavioral and physiological changes. This not only enhances the efficiency of disease monitoring but also supports dynamic assessment of treatment effects and personalized interventions, providing crucial data for clinical decision-making [20].

To the best of our knowledge, no bibliometric studies have been conducted in the general context of the field of digital biomarkers. While 2 comprehensive review articles have examined digital biomarkers for motor symptoms of PD [20] and nonmotor symptoms [7], they did not identify research hot spots or trends. Furthermore, their methodological limitations prevent them from providing an analysis of research output, funding, and collaboration patterns in the field. One emerging research hot spot identified in this study is the use of deep learning models for FOG, for which no targeted systematic reviews currently exist. Existing studies on deep learning models for FOG have included only 16 [26] and 9 [27] relevant articles, respectively, focusing solely on sensor devices and neglecting data collection from other digital devices. In algorithm models, these studies do not delve into critical details, such as sample acquisition and model construction methods. Moreover, due to advancements in both device technology and algorithm architectures, existing reviews fail to include the latest research and overlook certain relevant databases. In addition, this topic aligns with a recent initiative led by the FDA Digital Health Center, the Science and Engineering Laboratory, and the precision FDA platform of the Digital Transformation Office. They launched the “Digitally-Derived Endpoints for Freezing-of-Gait Detection Challenge,” aimed at identifying artificial intelligence (AI) and machine learning models that can detect and predict FOG events associated with PD [28].

Therefore, this study provides a comprehensive overview of the research landscape, hot spots, and trends in digital biomarkers for PD using bibliometric analysis and scoping review methods, while offering valuable insights for future research directions.

### Research Problem and Aims

This study aimed to (1) conduct a comprehensive analysis of the research output, major authors, leading countries, and contributions of the most productive academic institutions in Parkinson-related digital biomarker research; (2) highlight the research hot spots and future trends in the field; (3) explore the interdisciplinary collaboration models among researchers in this domain; (4) provide a scoping review on the hot topics.

## Methods

### Overview

**Figure 1:**
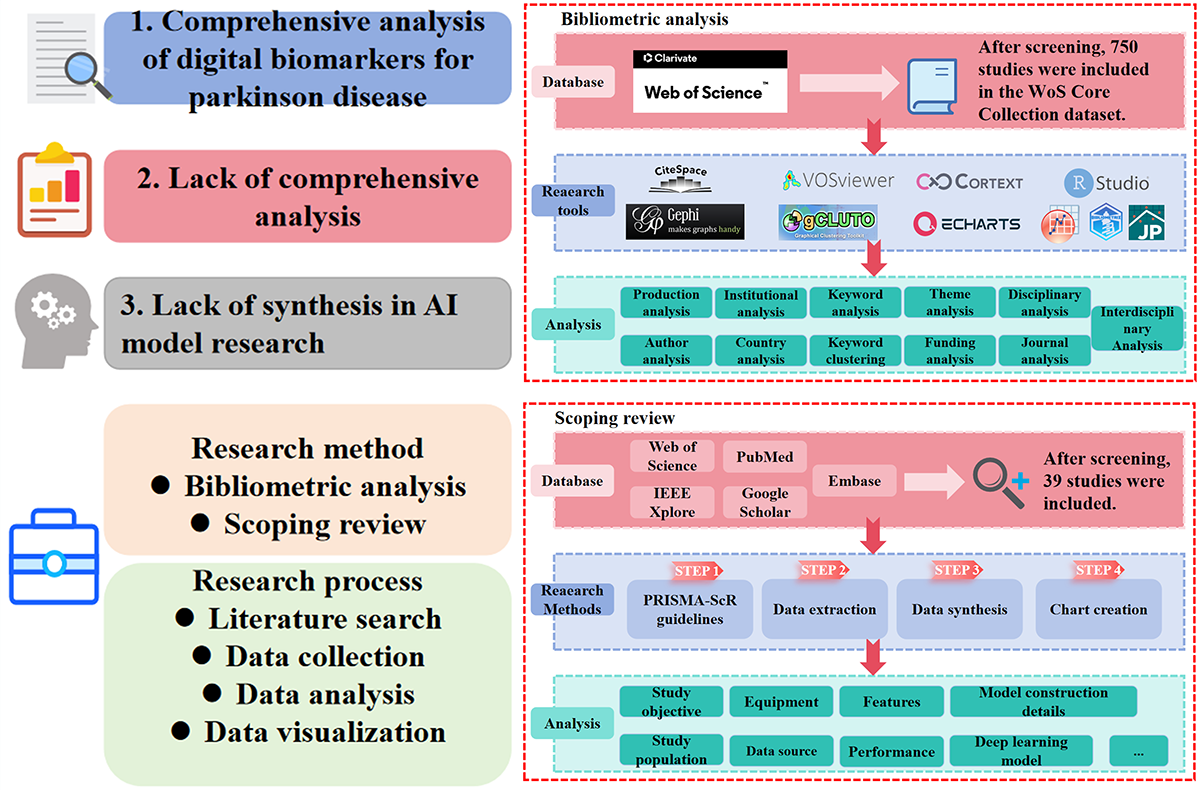
Specific framework and steps of bibliometric and scoping review research. AI: artificial intelligence; PRISMA-ScR: Preferred Reporting Items for Systematic Reviews and Meta-Analyses Extension for Scoping Reviews; WoS: Web of Science.

This study is divided into bibliometric analysis and scoping review sections, with the combination of these 2 methods providing complementary information, as has been implemented in the digital health field [29]. The first part uses bibliometric methods to examine the current state of the field from multiple dimensions, analyze the characteristics of researchers, and identify the status and trends of interdisciplinary collaboration. In addition, keyword analysis is performed to identify research trends and hot spots. The second part conducts an in-depth scoping review based on the identified hot topics from the first part, focusing on deep learning models for FOG digital biomarkers. This includes model paradigms, tasks, features, algorithms, and performance, covering the entire process from device data acquisition to FOG detection and prediction. An overview of the framework for specific methods is shown in Figure 1.

### Protocol and Registration

The scoping review section of this study follows the PRISMA-ScR (Preferred Reporting Items for Systematic Reviews and Meta-Analyses Extension for Scoping Reviews) guidelines and reports the search strategy used for literature selection [30]. The bibliometric analysis is conducted based on the bibliometric analysis framework proposed by Cobo et al [31]. The research protocol has been preregistered through the Open Science Framework.

### Data Sources and Search Strategy

Considering the need for standardization across different database formats and the interdisciplinary nature of the field, we compared preliminary search results from IEEE Xplore, Web of Science Core Collection (WoSCC), and PubMed databases, and ultimately selected Web of Science (WoS) as the primary database for this study. WoS has been widely used for interdisciplinary research analysis and has received positive feedback [32,33]. The search strategy was developed in collaboration with librarians, neurology experts, and medical informatics specialists, incorporating the Biomarkers, EndpointS, and other tools terminology [34]. Keywords, such as “Parkinson’s disease,” “digital biomarkers,” “digital health technologies,” and “diagnosis,” were used, and Boolean operators were used to construct the search query. To avoid daily updates of database resources, all search results were collected and exported in “plain text” format from the “Full Records and References” of WoSCC on October 1, 2024, with the specific search strategy provided in Multimedia Appendix 1.

The scoping review section conducted queries across 5 web-based databases, targeting interventions (ie, AI and deep learning) and the target disease (ie, FOG): WoS, IEEE Xplore, PubMed, Google Scholar, and Embase. We conducted an extensive search on Google Scholar, sorted the results by relevance, and included only the top 100 studies. To ensure comprehensive inclusion, backward and forward citation checks were performed. The search time span extended from the inception of the databases to December 26, 2024, with the specific search strategy provided in Multimedia Appendix 2.

### Inclusion and Screening Strategy

The detailed inclusion and exclusion strategy for the bibliometric section is provided in Textbox 1.

**Inclusion and exclusion strategy for the bibliometric section.**

**Inclusion criteria**

- The study must use data collected from wearable or digital devices, which are considered digital biomarkers; studies using other forms of behavioral or physiological data streams are excluded.
- The document type must be “Article,” excluding nonresearch articles, such as scoping reviews, meta-analyses, and systematic reviews.
- The study must be written in English.
- The study must have been peer reviewed and published in a journal.
- The digital biomarkers in the study must be used for the diagnosis, identification, or assessment of Parkinson disease or related symptoms.

**Exclusion criteria**

- Studies for which the full text is not accessible
- Duplicate publications
- Books, editorials, commentaries, and retracted studies
- The research focuses on the development of digital devices, applications, or tools, but does not involve the collection of patient data or actual use

In the scoping review section, we focus on research related to deep learning models for FOG. Specifically, we excluded literature reviews, papers, and studies that are purely based on clinical trials or experimental research summarizing FOG deep learning methods. Only journal articles were included, while case reports, reviews, dissertations, proposals, conference abstracts, editorials, and general comments were excluded. In addition, studies that did not use deep learning techniques, such as threshold-based methods or traditional machine learning algorithms, were excluded. There were no restrictions regarding the study location, study design, study outcomes, month of publication, or the country of publication.

**Figure 2:**
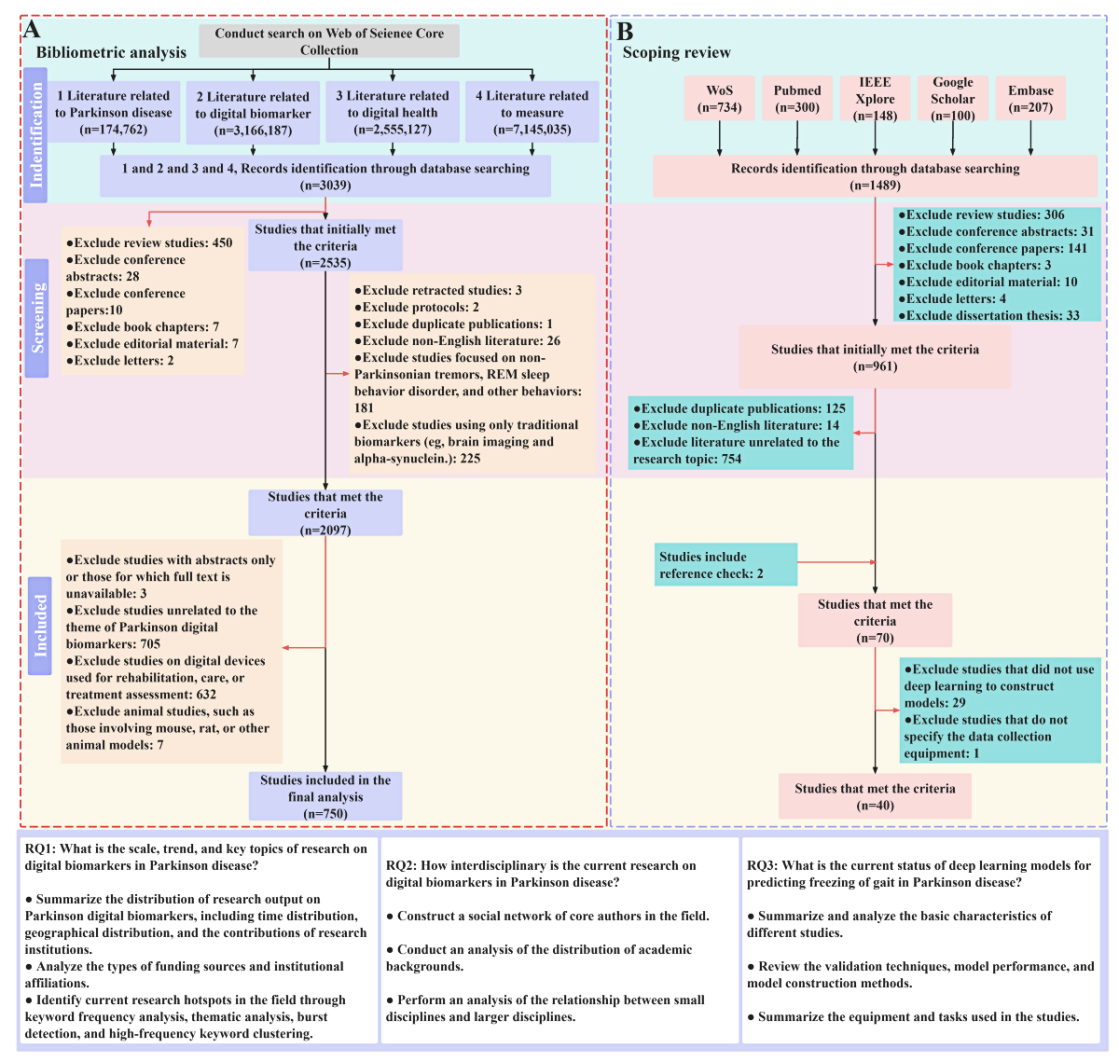
The specific process of literature screening and research content for bibliometric and scoping review studies. REM: rapid eye movement; RQ: research question; WoS: Web of Science.

During the screening process of the bibliometric analysis, 50 studies were assigned to 2 evaluators (WQ and SS) for initial screening according to the aforementioned criteria, resulting in a Cohen κ coefficient of 0.88 [35]. In case of any disagreements during the overall screening process of the 2 sections, SC intervened for decision-making. The bibliometric section of the screening was completed on October 15, 2024, while the scoping review was completed on December 30, 2024. The specific scheduling flowchart is shown in Figure 2.

### Data Extraction and Synthesis for the Scoping Review

To accurately extract data from the included studies, we followed the PRISMA-S guidelines and created a data extraction form using Microsoft Excel, which was independently completed by 2 evaluators. Any discrepancies between the evaluators were resolved through discussion. The checklist for this study is provided in Multimedia Appendix 3. We used a narrative approach to synthesize the data, focusing on summarizing and describing the deep learning methods used in each study, including their objectives (eg, identification and prediction), features, data sources (eg, participants and datasets), and the AI models used (eg, convolutional neural networks [CNNs]). In addition, we described the data acquisition devices and specific data types for the model inputs (eg, wearable devices and cameras), as well as the statistical metrics used (eg, accuracy, specificity, sensitivity, and precision).

### Data Cleaning

Data cleaning is a critical step in bibliometric research. In the keyword section, we created a merged keyword bibliography to combine synonyms, singular, and plural forms, among others, to ensure the accuracy of data analysis. For example, “parkinson disease” was merged under the “parkinson’s disease” category. A list of all merged keywords is provided in Multimedia Appendix 4. We standardized the full names and abbreviations of authors, institutions, and funding sources [32]. For authors affiliated with multiple institutions, we applied the institution cleaning method developed by Nam et al [36], selecting the institution in the first column that is typically considered the primary affiliation.

### Data Analysis

In the bibliometric section, to ensure the accuracy of multidimensional analysis, we adopted a multitool joint analysis strategy, using CiteSpace (Version 6.2.R6 Advance; Drexel University) [37], Vosviewer (Version 1.6.19; Leiden University) [38], Cortext (Gustave Eiffel University) [39], Bibliometrix [40], Gephi (Version 0.10.1; Gephi) [41], gCLUTO (Version 1.0; Kerapis Lab) [42], and Joinpoint [43] for bibliometric analysis. The complementary strategies for the use of each tool are provided in Multimedia Appendix 5.

The Joinpoint software is used to systematically evaluate time trends and test the statistical significance of trends between junction points. The software determines the number of inflection points in the model based on the recommended procedures. To facilitate the display of trend direction and magnitude, this study calculated the changes in trend slopes [44].

CiteSpace is primarily used for keyword burst analysis to identify recent emerging trends. Vosviewer is used for author and institution collaboration network analysis, as well as bibliometric coupling analysis of journals. In author analysis, we applied the law developed by Price [45] to calculate the output of core authors, setting thresholds to construct maps. For institution collaboration analysis, to comprehensively display the institutional cooperation in the field, we included institutions with an output of 2 or more. All analyses were conducted using full counting. Nodes with the same color represent the same cluster, node size reflects output volume, and the thickness of connecting lines indicates collaboration strength. In addition, we categorized all institutions into universities, hospitals, companies, research institutes, and government departments [36], calculated the output of each type of institution, and constructed matrices to visualize the cooperation among different types of institutions.

The Bibliometrix package in R (R Foundation for Statistical Computing) provides higher granularity in general project analysis compared to CiteSpace and Vosviewer. Therefore, we used Bibliometrix to analyze the thematic evolution of high-frequency keywords, using the Walktrap clustering algorithm and conducting thematic analysis based on “keyword plus” to identify the main research topics, their relative importance, interconnections, and developmental trends. In addition, we used gCLUTO to cluster the high-frequency keywords. A hierarchical clustering method with repeated partitioning was applied, and similarity was calculated using the cosine function. The clustering criterion function was set to I^2^, and results with high internal similarity and low external similarity were selected for display. The results were visualized using matrices and hill charts to identify more detailed hot topics [32].

We classified funding sources and funding models into 6 categories: government funding, nonprofit organization funding, corporate funding, university and research institution funding, international organization funding, and individual funding [46]. In addition, using Cortext, we constructed heat matrix diagrams to explore the relationships between funding and disciplines, as well as between funding and countries, to further investigate the funding patterns. The research domains in all WoS product databases are categorized using a shared thematic classification scheme, which includes 21 broad subject groups and 254 specific subdisciplines. The distinction between broad and specific subject categories creates a 2-tier classification system [47]. Broad subject categories are macrolevel classifications based on academic disciplines, encompassing the main research directions and offering a wide thematic division. Specific subdisciplines, in contrast, are more granular classifications under the broad categories, providing a more precise academic segmentation by focusing on particular research directions and fields. Each paper is assigned to at least one thematic category, which maps to a specific research domain [33]. This approach enables the analysis of the distribution of disciplines within the field and the interdisciplinary publication characteristics.

Digital biomarker research is a highly interdisciplinary field. Following the method proposed by Agha-Mir-Salim et al [48], we categorized the academic disciplines of the authors of each study into those related to PD (eg, neurology, geriatrics, psychology, psychiatry, and other medical fields) and those related to digital biomarker technologies (eg, computer and communication engineering, bioinformatics, medical informatics, and other engineering disciplines). Disciplines not falling into the above categories were classified as “other disciplines.” The discipline classification was primarily based on author information from ResearchGate and degree information in the article. In cases of missing information, academic profiles, Open Researcher and Contributor ID, institutional websites, or third-party platforms (eg, LinkedIn) were consulted. If an author’s disciplinary background could not be determined, they were excluded from the analysis. The participation rate of each discipline was calculated by determining whether researchers from that discipline participated in a given study, representing the proportion of studies contributed by that discipline. The strength of interdisciplinary collaboration was measured by constructing a 10×10 matrix, which was visualized using Gephi. This matrix was based on the participation of researchers from different disciplines in each paper.

### Ethical Considerations

Ethics committee approval was not required because this study was a retrospective bibliometric analysis of existing published studies.

## Results

### The Annual Trends of Publications

By conducting a search in the WoSCC database, we obtained a total of 3039 publications. After screening, 750 (24.68%) studies were included in the analysis, with an average of 39.47 publications per year. In 2018, the number of publications exceeded 50 for the first time, reaching 59 (7.87% of the total). The output peaked at 94 publications in 2022.

**Figure 3:**
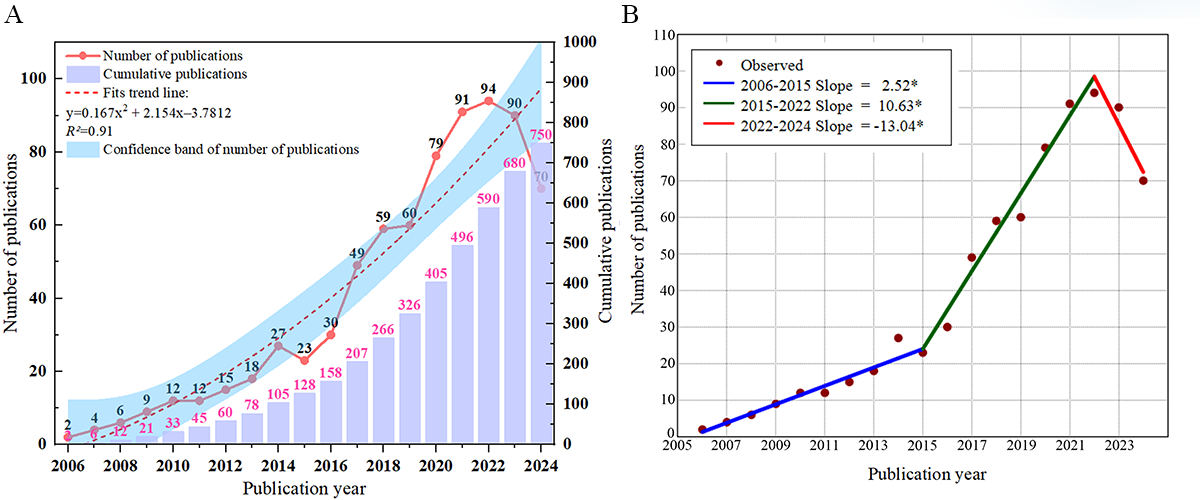
Distribution of Parkinson digital biomarker research output. (A) Annual output distribution and trend graph. (B) Change point year analysis graph.

To visually present the trend in publications, we performed a binomial fit using Origin (Origin 2021; OriginLab Corporation), with the red dashed line representing the fitted trend. Although the search cutoff date was set for October 2024—meaning that not all relevant studies from 2024 were included—the overall trendline shows an upward trajectory (*R*²=0.91, indicating a good model fit that accurately reflects the publication growth trend), as shown in Figure 3A. Through breakpoint regression analysis, we identified 2015 as a potential change point, with the following slope calculations for 3 periods: slope 1 (2006 to 2015)=2.52, slope 2 (2015 to 2022)=10.63, and slope 3 (2022 to 2024)=−13.04, as shown in Figure 3B.

### Author Analysis

**Figure 4:**
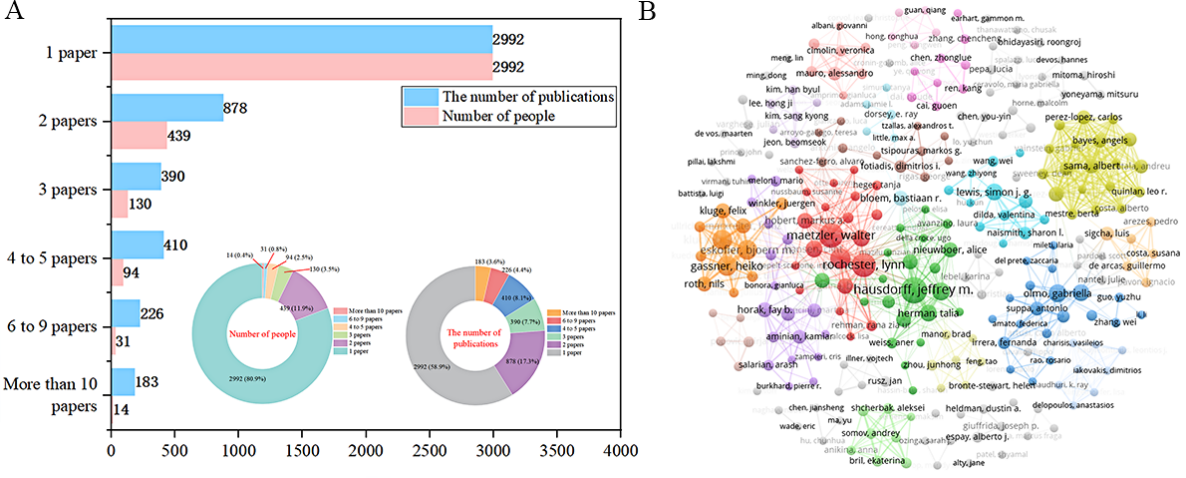
Author analysis overview. (A) Author publication distribution. (B) Core author collaboration network map.

A total of 3700 researchers contributed to the publication of 5079 studies. Among them, 2992 (80.9%) researchers published only one study, contributing 58.9% (2992/5079) of the total publication output. A further 439 (11.9%) researchers published 2 studies, accounting for 17.3% (878/5079) of the total output. According to the law developed by Price [45], the minimum threshold for core authors is 3 published papers. A total of 269 (7.2%) researchers reached this threshold, contributing 1209 (23.8%) articles. However, this still does not meet the core author output threshold (>50%) as stipulated by the law developed by Price, as shown in Figure 4A.

The highest-output researcher was Jeffrey M Hausdorff from Tel Aviv University, with 20 publications. He was followed by Walter Maetzler from Kiel University, with 19 publications, and Lynn Rochester from Newcastle University, with 18 publications. A list of other researchers with more than 10 publications is provided in Table S1 in Multimedia Appendix 6. From the perspective of author bursts, a total of 8 researchers, each with more than 10 publications, ranked among the top 25 in publication bursts strength. Notably, Lynn Rochester exhibited the highest emergence intensity between 2017 and 2020 (strength=3.83), while Clint Hansen and Felix Kluge had the highest bursts strength between 2021 and 2024, both with a score of 3.09. More information on the top 25 researchers by publication bursts strength is provided in Figure S1 in Multimedia Appendix 6.

For core authors, we constructed a collaboration network map. Overall, the cooccurrence network within the core author clusters was more robust, with numerous connections, while the network outside these clusters was relatively independent, exhibiting fewer links. This demonstrates a characteristic of high cohesion and low coupling. The network dominated by the top 10 highest producers was more developed compared to others, and these authors often drove the research within a specific community but collaborated less with other communities. For instance, high-output researchers such as Walter Maetzler, Lynn Rochester, Silvia Del Din, Daniela Berg, and Clint Hansen were clustered in the red group, with members primarily from Kiel University and Newcastle University. Jochen Klucken, Heiko Gassner, and Björn M Eskofier were located in the orange cluster, with 2 members from Friedrich-Alexander-University Erlangen-Nürnberg. Jeffrey M Hausdorff and Talia Herman were positioned in the green cluster, both from Tel Aviv University, as shown in Figure 4B.

### Cross-Disciplinary Collaboration Analysis

There were differences in the participation of researchers from different disciplinary backgrounds in the field of Parkinson digital biomarkers. After surveying and categorizing the disciplinary backgrounds and research experience of 3700 researchers, 3043 (82.2%) researchers were assigned specific disciplinary backgrounds. Among those with a medical background, 1352 (44.4%) specialized in neurology, 94 (3.1%) in psychiatry, 133 (4.4%) in geriatrics, 71 (2.3%) in psychology, and 347 (11.4%) in other medical fields. Among those with an engineering background, 471 (15.5%) specialized in computer or communication engineering, 88 (2.9%) in biomedical or medical engineering, 49 (1.6%) in medical informatics, and 183 (6.0%) in other engineering fields.

**Figure 5:**
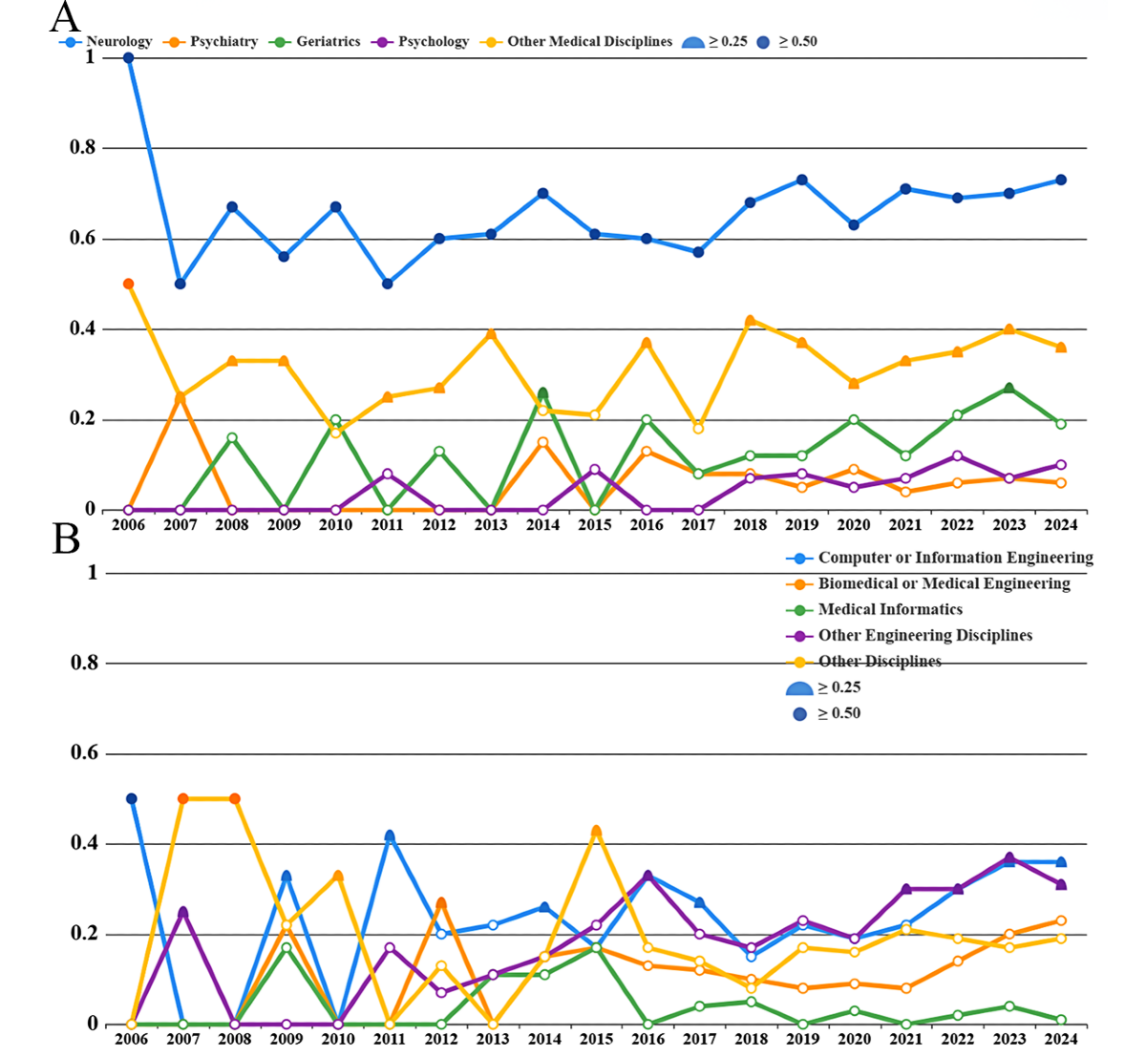
The participation rate of various disciplines and the collaboration network diagram. (A) Annual participation rate trends in medical-related disciplines. (B) Annual participation rate trends in engineering and other disciplines.

There were significant differences in the participation rates of researchers from various disciplinary backgrounds in the field of Parkinson digital biomarkers. First, among medical-related disciplines, neurology had the highest participation rate, consistently exceeding 50% each year, suggesting that more than half of the research published each year involved researchers with a neurology background. The second highest participation rate was observed in other medical disciplines, which remained stable above 25% but did not surpass the 50% threshold, indicating a certain level of attention in this field. In contrast, psychology, geriatrics, and psychiatry had relatively lower participation rates, typically staying below 25% annually. However, with the overall increase in publications in recent years, the fluctuations in these disciplines’ participation rates have become smoother, especially geriatrics, which showed a potential upward trend (Figure 5A). In engineering-related research, no single discipline had a significantly leading participation rate. Notably, starting from 2015, the participation rates in these disciplines had stabilized and showed a slow growth trend. In particular, the participation rates in other engineering disciplines and computer or communication engineering surpassed 25% in recent years, while other disciplines’ participation rates were close to 20% (Figure 5B).

**Figure 6:**
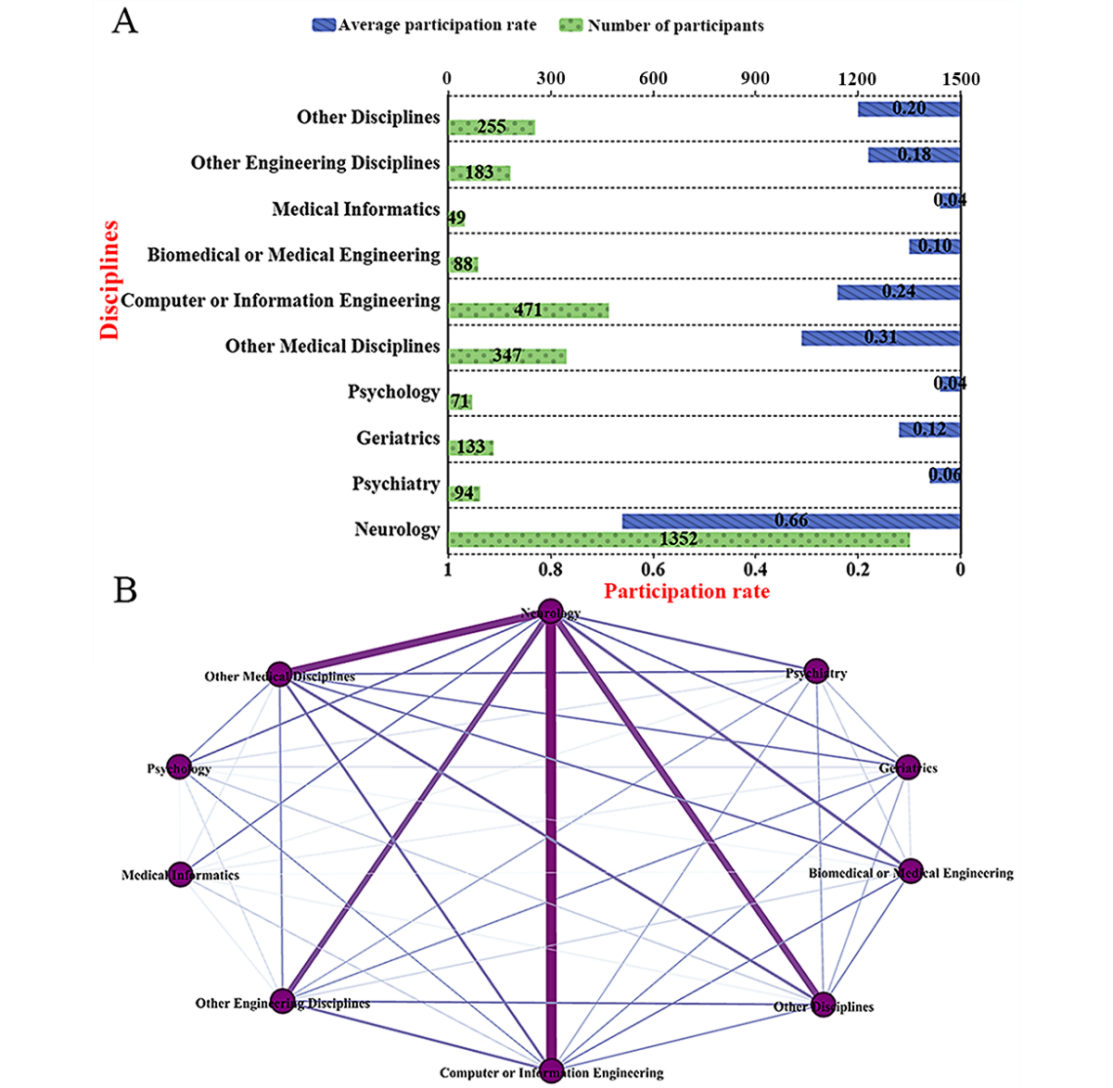
Participation of researchers from different disciplines. (A) Average participation rate by discipline. (B) Collaboration network among disciplines.

We also calculated the average participation rates for each discipline. Neurology had the highest average participation rate at 66%, followed by other medical disciplines (31%) and computer or communication engineering (24%; Figure 6A). Figure 6B illustrates the collaboration network between disciplines, highlighting the cooperative relationships among various fields. Neuroscience showed strong collaboration with computer or communications engineering, other medical disciplines, other engineering fields, and other disciplines. Specifically, 132 (17.6%) studies involved collaboration with computer or communications engineering; 97 (12.9%) studies collaborated with other medical fields; 84 (11.2%) studies involved cooperation with other disciplines; and 64 (8.5%) studies were in collaboration with other engineering fields. Collaboration among other disciplines, whether within medical or engineering fields or across these 2 domains, was relatively weak. This reflects the current situation in the field, where interdisciplinary cooperation is still limited.

### Institutional Analysis

Institutional analysis reveals the organizational structure of the research field. A total of 1384 institutions contributed to the publication of 2448 studies. The distribution was as follows: 769 universities published 1556 (63.6%) studies, averaging approximately 2 studies per university; 333 hospitals published 503 (20.5%) studies, averaging 1.5 studies per hospital; 177 research institutions and government organizations published 254 (10.4%) studies, averaging 1.4 studies per institution; and 105 companies published 135 (5.5%) studies, averaging 1.3 studies per company.

Furthermore, among the institutions with more than 10 publications, there were 22 institutions, including 1 hospital, 1 research institution, and the remaining were universities. These institutions contributed a total of 308 (12.6%) studies. Tel Aviv University in Israel had the highest output, with 27 (1.9%) publications; followed by Newcastle University (n=26, 1.8%) in the United Kingdom and Tel Aviv Sourasky Medical Center in Israel (n=21, 1.4%). Additional details on high-output institutions are provided in Table S2 in Multimedia Appendix 6.

**Figure 7:**
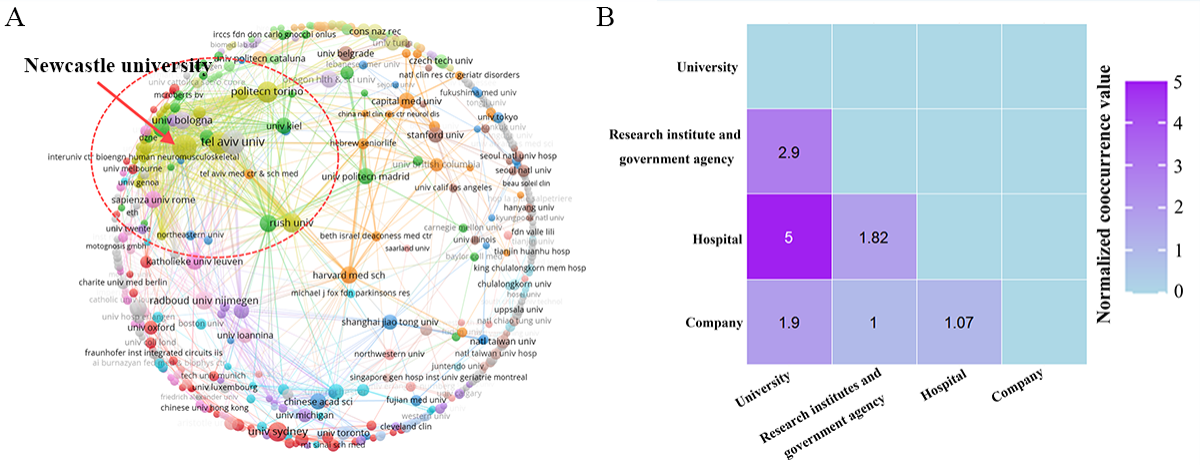
Overview of institution analysis. (A) Institutional collaboration network map. (B) Heatmap of collaboration intensity matrix between different types of institutions.

A total of 381 (27.5%) institutions published 2 or more studies, contributing 1445 (59%) studies in total. Specifically, among them, 260 (68.2%) were universities, which published 1047 (72.5%) studies; 67 (17.6%) were hospitals, with 237 (16.4%) publications; 33 (8.7%) were research institutions or government organizations, publishing 110 (7.6%) studies; and 21 (5.5%) were companies, with 51 (3.5%) publications. The collaboration network showed that the primary collaborative network was centered around the yellow cluster of Newcastle University, which had collaborations with 54 institutions, with a total link strength of 114. Although many other institutions also had collaborations, they did not form as tightly connected networks, as shown in Figure 7A.

Collaboration between different types of institutions was a key form of interinstitutional cooperation, with 39.5% (799/2025) instances of cross-institutional type collaboration. The analysis showed that universities were the most active in collaborative research, particularly in partnerships with hospitals, which occurred 318 (39.8%) times. The second most frequent collaboration was with research institutes or government organizations, with 175 (21.9%) instances. In contrast, collaborations between companies and research institutes or government organizations were the least frequent, with only 41 (5.1%) instances. We used a cooccurrence matrix to visualize the collaboration intensity between different types of institutions and normalized the cooccurrence values to facilitate comparison of the relative differences in collaboration across institution types (Figure 7B).

### Country Analysis

**Figure 8:**
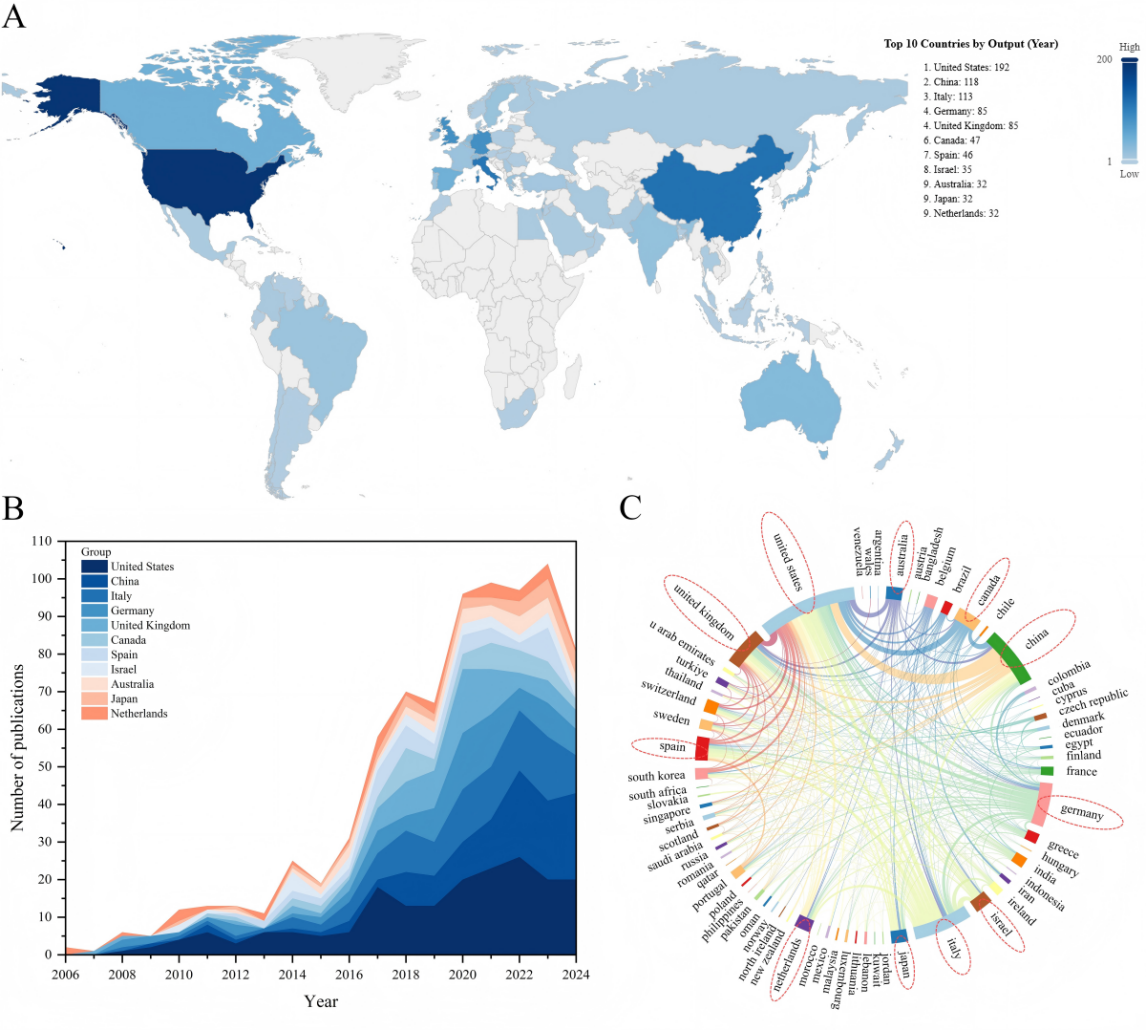
Country analysis. (A) Global distribution map of Parkinson digital biomarker research output by country. (B) Annual output distribution trends of high-producing countries. (C) International collaboration Sankey diagram of Parkinson digital biomarker research by country.

A total of 61 countries contributed to research publications in this field, with an aggregate of 1171 studies. These countries were distributed across various continents, with 26 (43%) countries from Europe and 19 (31%) from Asia. The specific distribution of research output by country is shown in Figure 8A.

The top 10 countries (actually 11 countries) contributed 810 (69.2%) studies. Among them, the United States led with 192 (16.4%) publications, followed by China with 118 (10.1%) and Italy with 113 (9.6%). Notably, 5 of the high-output countries were from Europe (Italy, Germany, the United Kingdom, Spain, and the Netherlands), with a combined output of 354 (30.2%) studies.

There were differences in the sustained attention to Parkinson digital biomarkers research among high-producing countries. Overall, these high-output countries had consistently contributed since 2015, with a rapid growth trend. The United States, Italy, and Germany began focusing on this topic as early as 2008, maintaining a steady research output, indicating their pioneering contributions in this field. In contrast, China, the second-highest contributor, did not stabilize its output until 2014, and in recent years has reached an output close to that of the United States, reflecting China’s growing interest in this topic. The distribution of output and sustained contributions from high-output countries is shown in Figure 8B, with detailed information available in Table S3 in Multimedia Appendix 6.

Among the 61 countries participating in the research, most (58/61, 95%) engaged in international collaboration, with few (3/61, 5%) countries publishing all their research independently. High-output countries generally exhibit a broader willingness to collaborate, especially in tighter cooperation among high-output nations. Specifically, there were 742 instances of cooperation among the 61 countries. The United States had the broadest international collaboration, partnering with 36 (59%) countries, resulting in 193 joint studies. Notably, collaborations with 8 (22%) of these countries led to more than 10 publications each. The United States collaborated most frequently with China (17 studies, 8.8% of total collaborations), followed by the United Kingdom (16/193, 8.3%) and Israel (16/193, 8.3%). The details of international collaboration publications are shown in Figure 8C.

**Figure 9:**
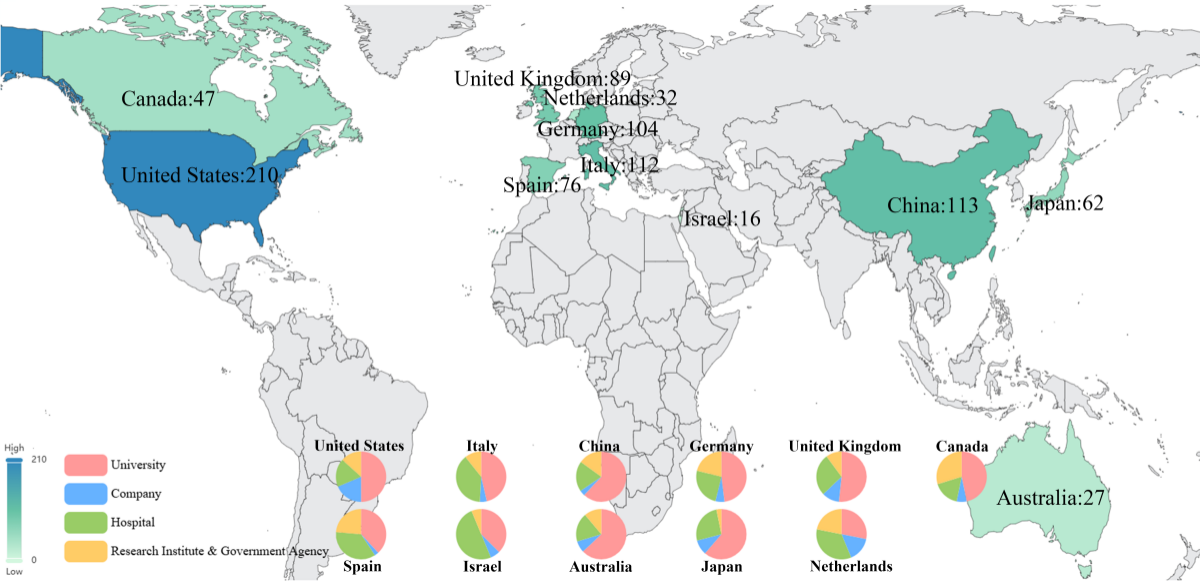
Number of institutions and distribution by type in high-producing countries.

A further analysis of the institutional distribution of high-producing countries helped to understand the output patterns of different nations. Regarding the number of institutions, the United States had the most (210/1384, 15.2%) institutions involved in research, followed by China (113/1384, 8.2%) and Italy (112/1384, 8.1%). Regarding institution type, research in most countries was primarily led by universities, particularly in China, Australia, Japan, the United States, and the United Kingdom, where more than half (276/501, 55.1%) of the institutions were universities. In contrast, hospitals in Israel stood out in the research on digital biomarkers, with half (8/16, 50%) of the research institutions being hospitals. In addition, except for the United States, the participation of companies in research was relatively low in most countries, especially in China. The detailed institutional distribution is shown in Figure 9.

### Funding Analysis

**Figure 10:**
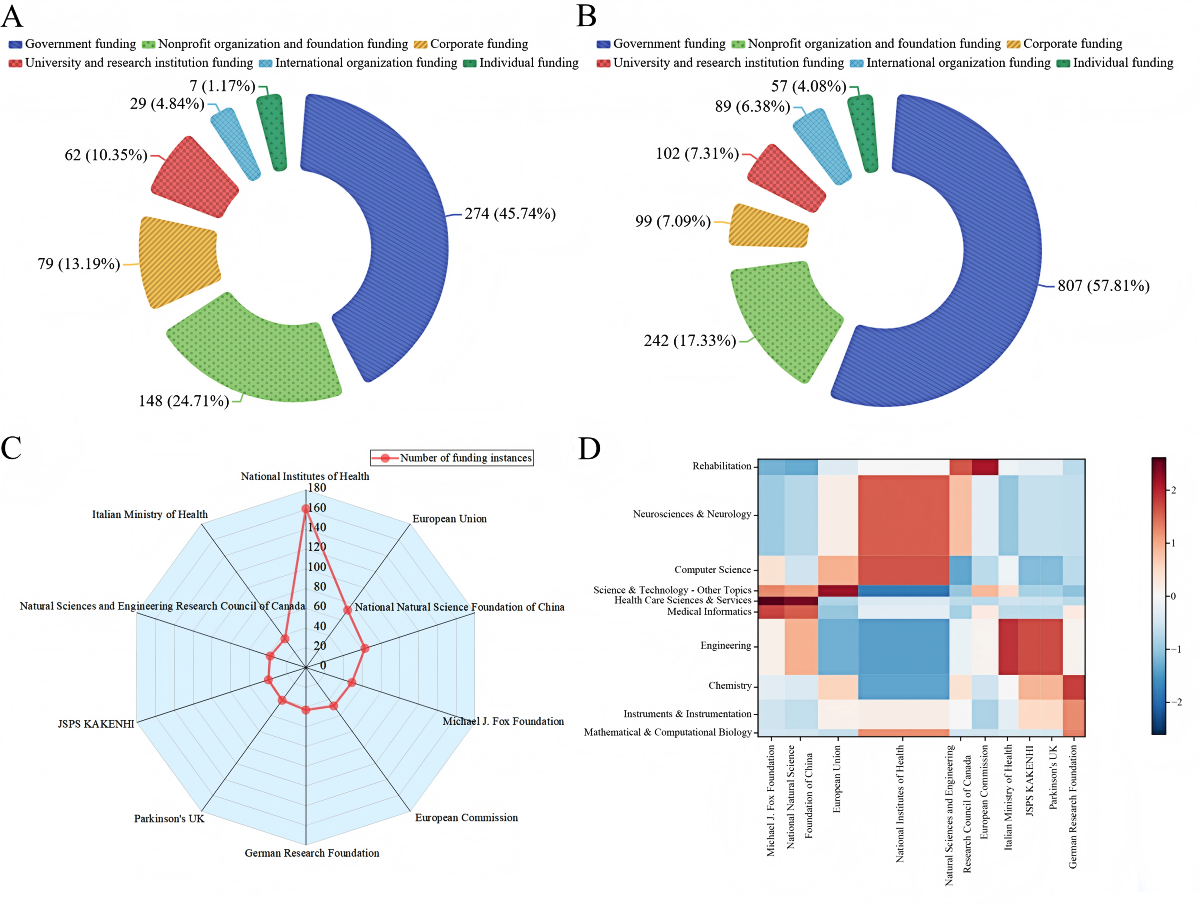
Funding project analysis overview. (A) Distribution of funding numbers by type. (B) Distribution of research output by funding type. (C) Distribution of top 10 funding projects by number of instances. (D) Distribution of disciplines funded by top 10 funding projects. JSPS: Japan Society for the Promotion of Science; UK: United Kingdom.

A total of 599 funding projects supported 1396 studies in this field, with an average of 1.86 instances of funding per study. In terms of funding sources, government agencies provided the most support, with 274 (45.7%) funding projects supporting 807 (57.8%) studies. Nonprofit organizations and foundations provided 148 (24.7%) funding projects, supporting 242 (17.3%) studies. Personal funding projects were the least common, with only 7 (1.2%) projects, but they supported 57 (4.1%) studies. The specific distribution of funding types is shown in Figures 10A and 10B.

Among the top 10 funding projects by number of instances, the National Institutes of Health (NIH) supported 161 (11.5%) studies, followed by the European Union, which funded 72 (5.2%) studies. Detailed information on the top 10 funding projects is provided in Table S4 in Multimedia Appendix 6 and Figure 10C.

Figure 10D shows the funding connections between the top 10 funding projects and disciplines, visualized through a cooccurrence matrix representing the strength of associations between each discipline and funding project. We found that the NIH primarily funded the disciplines of Neurosciences and Neurology and Computer Science, with Neurosciences and Neurology receiving the most funding. The Michael J. Fox Foundation and the National Natural Science Foundation of China mainly funded Medical Informatics and Health care Science & Services, though these 2 disciplines received relatively less funding. The Italian Ministry of Health, Japan Society for the Promotion of Science KAKENHI, and Parkinson's UK were more focused on funding research in the field of Engineering.

### Disciplinary Publication Analysis

**Figure 11:**
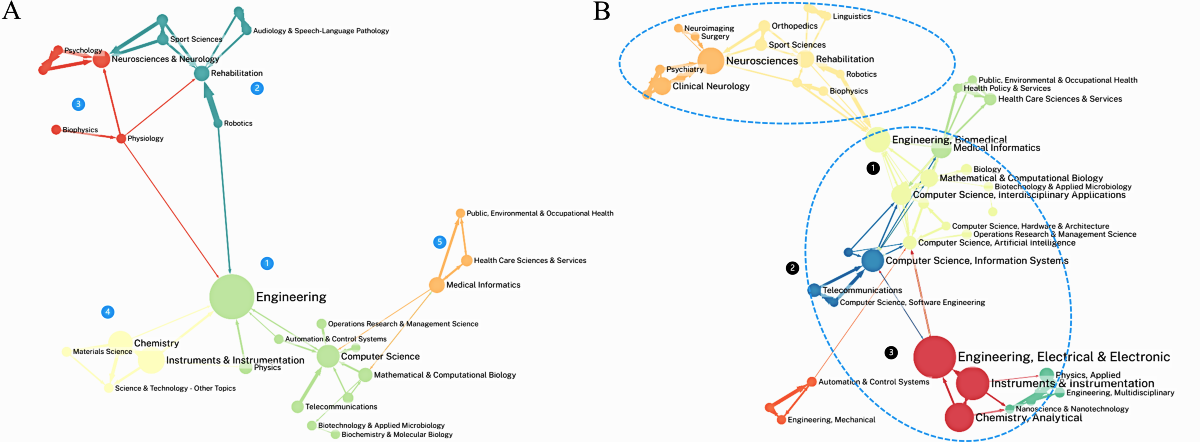
Disciplinary analysis clusters and matrix. (A) Clustering results based on Web of Science major disciplines. (B) Clustering results based on Web of Science subdisciplines.

Disciplinary publication analysis helps to clarify the structure of the knowledge system in Parkinson digital biomarkers research. We identified interdisciplinary connections between 29 major disciplines and 45 subdisciplines in this field. From a major discipline perspective, the 29 disciplines can be clearly divided into 5 clusters, with medical-related disciplines being more distant and less connected to engineering-related disciplines. The largest cluster, cluster 1, centered on engineering and computer science, forming the engineering and technology framework for digital biomarker research. Cluster 2 and cluster 3 were closely related, with neurosciences and neurology, and rehabilitation at their core, together forming the knowledge system for PD and its related symptoms. Cluster 4 focused on instruments and instrumentation, chemistry, and materials science, providing insights into the development and application of research equipment. Cluster 5 represented interdisciplinary fields like medical informatics and health care science and services, focusing on both medical diagnosis and the application of technology. Detailed information is shown in Figure 11A.

After refining the analysis to 45 subdisciplines, a more complex disciplinary structure emerged. The 2 clusters centered on neurosciences and rehabilitation remained closely connected and had further differentiated into multiple fields. The most significant change was that the originally technology-focused cluster, centered on engineering and computer science, had evolved into 3 more refined clusters. Among these, cluster 1 was most closely related to the medical clusters focused on PD and its symptoms. This cluster integrated interdisciplinary applications from bioengineering, computer science, and fields like AI, providing important analytic and technical support for digital biomarkers research. Cluster 2 emphasized software engineering and system applications, focusing more on hardware related to data collection devices. Cluster 3 was primarily composed of branches of electronic engineering, which, although more distanced from the other 2 clusters, played a key supporting role in the fields of data acquisition devices and sensors, especially in instruments and instrumentation. The specifics are shown in Figure 11B.

### Journal and Highly Cited Literature Analysis

Bibliographic coupling is one of the most prominent citation-based coupling methods for measuring similarity between scientific projects. It determines the most influential journals by assessing the number of third-party references shared between 2 related projects in their citation lists [49]. In the journal cocitation analysis, 3 main clusters were observed as follows

**Figure 12:**
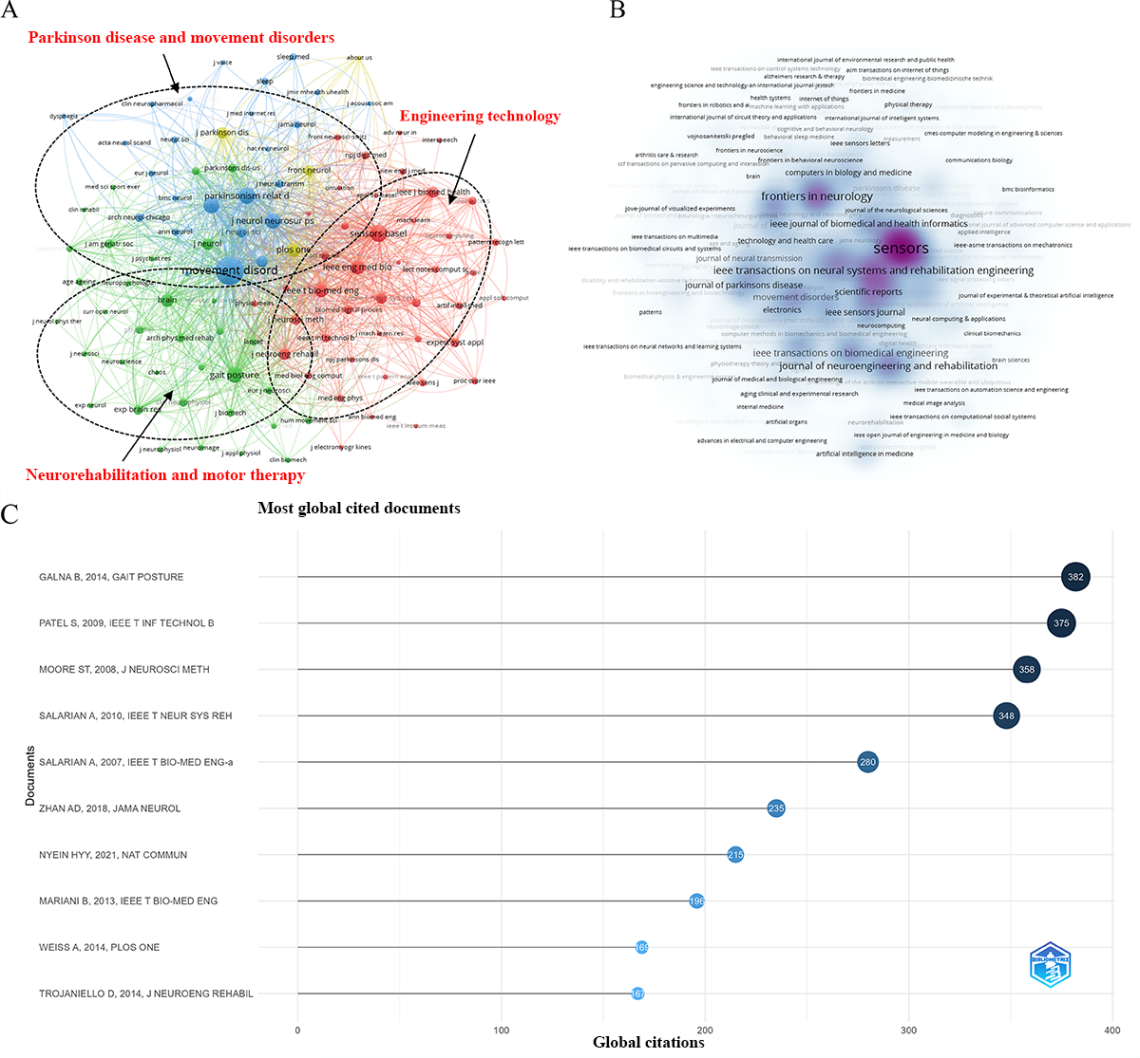
Journal and highly cited literature analysis. (A) Co-cited journal clustering map. (B) High-output journal distribution map. (C) Highly cited literature distribution map.

- Red cluster: Key journals included Sensors-Basel, IEEE Engineering in Medicine and Biology, and IEEE Biomedicine and Health. This cluster exhibited a strong interdisciplinary nature, integrating engineering technology with the medical field. It represented cross-disciplinary research in the areas of sensors, biomedical engineering, and digital health.
- Blue cluster: It is closely linked to research on PD and other neurodegenerative disorders, this cluster represents in-depth research on PD and similar neurological conditions. The main journals in this cluster included Parkinsonism and Related Disorders, Journal of Parkinson’s Disease, and Movement Disorders.
- Green cluster: This cluster focused on kinematics, rehabilitation, and physiology, with an emphasis on motor function recovery in neurological diseases. It is a key research area in neuroscience and motion monitoring. The leading journals in this cluster included Brain, Gait and Posture, Clinical Rehabilitation, and Physical Therapy. Detailed information can be found in Figure 12A and Table S1 in Multimedia Appendix 7 [50-59].

Regarding publication output, the journal Sensors (Multidisciplinary Digital Publishing Institute) had published the most studies on the topic, reaching 100 (13.3%) articles. It was followed by Frontiers in Neurology and IEEE Transactions on Neural Systems and Rehabilitation Engineering, with 32 (4%) and 28 (4%) articles, respectively. Information on high-output journals is presented in Figure 12B and Table S2 in Multimedia Appendix 7.

Regarding globally highly cited literature, the most-cited study was research published in the journal Gait and Posture, which confirmed that the Microsoft Kinect sensor can accurately measure Parkinson motor symptoms [50], with 382 citations. In addition, studies published in Journal of the American Medical Association Neurology and Nature Communications received the highest annual citations. The study on the use of smartphones and machine learning to accurately quantify Parkinson’s severity [51] in Journal of the American Medical Association Neurology achieved an average of 29.38 citations per year, while a study on developing wearable patches to monitor levodopa concentration in patients’ sweat [52] in Nature Communications received 43 citations per year. Information on highly cited studies is shown in Figure 12C [50-54] and Table S3 in Multimedia Appendix 7.

### Keyword Theme Analysis

After data cleaning, 1464 keywords were identified, with a cumulative frequency of 3983 occurrences. A thematic term analysis of these keywords was performed to explore the core topics in PD digital biomarker research. In the “Motor” theme, clusters demonstrated higher centrality and density, indicating that this theme has matured and plays a crucial role in shaping the research framework. Currently, research on disease classification and rapid eye movement (REM) sleep behavior disorder analysis based on deep learning technology falls within this quadrant, highlighting its importance as a central research direction.

**Figure 13:**
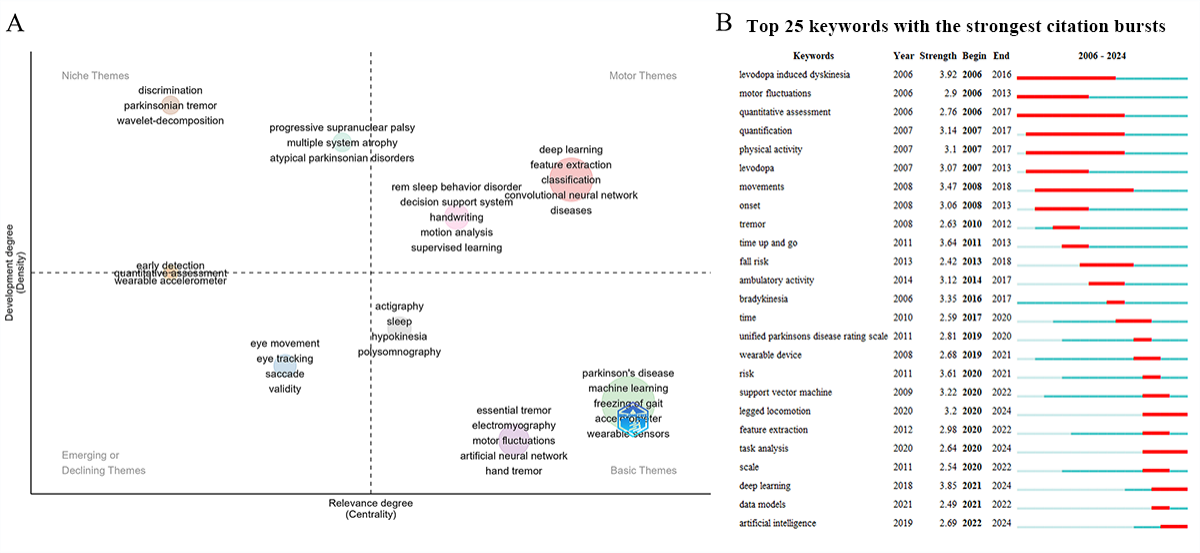
Keyword analysis. (A) Keyword theme trend analysis. (B) Keyword burst distribution.

In contrast, topics in the emerging or declining quadrants showed lower centrality and density, suggesting that these themes are underdeveloped and still in peripheral stages. Therefore, eye-tracking-based research may still be in its preliminary phase. Furthermore, tremor detection based on neural networks, machine learning-driven FOG detection, and multidimensional sleep mapping for sleep monitoring were all situated within foundational themes, underscoring their significance in the field’s development. In the niche theme quadrant (upper-left), 2 primary research directions were evident: tremor signal analysis in PD and neurodegenerative diseases closely resembling Parkinson. Although these topics are relatively specialized, their importance in the identification and diagnostic processes of PD should not be overlooked (Figure 13A).

Further burst analysis of the keywords identified the top 25 keywords with the highest burst intensity. Among these, “legged locomotion,” “task analysis,” “deep learning,” and “artificial intelligence” emerged as recent burst keywords, indicating that AI and limb movement analysis have become key research hot spots in recent years (Figure 13B).

### Keyword Clustering

**Figure 14:**
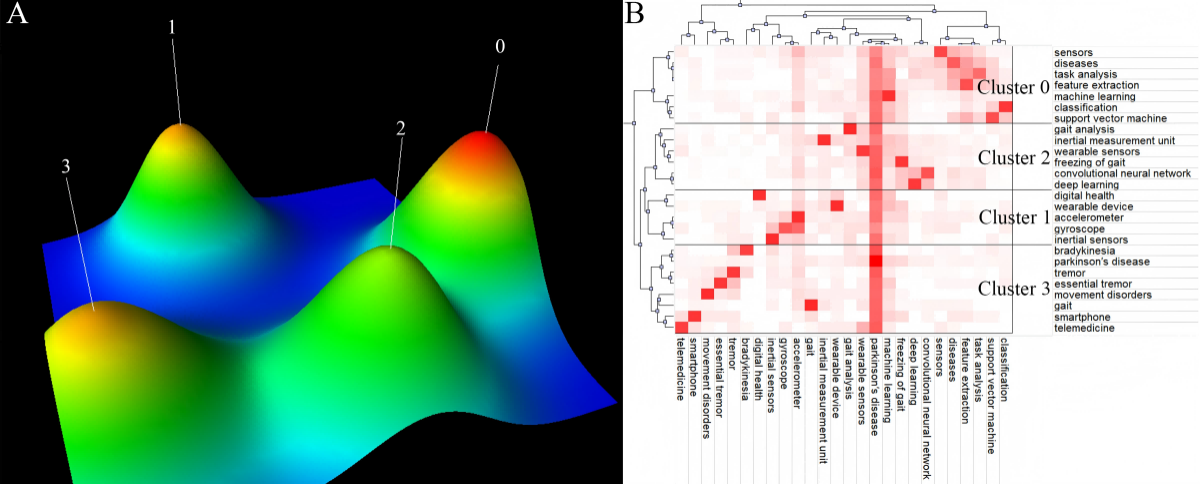
High-frequency keyword clustering. (A) High-frequency keyword cluster hill diagram. (B) High-frequency keyword hierarchical clustering diagram.

To further explore and refine the emerging topics in this field, a clustering analysis was performed on high-frequency keywords. According to the law developed by Price, the minimum frequency for high-frequency keywords was calculated to be 16.6 occurrences. A total of 26 keywords were selected for clustering, with a cumulative frequency of 37.9% (1510/3983) occurrences, as shown in Multimedia Appendix 8. In total, 4 clusters were identified, each represented as a unique “hill,” where the height and volume corresponded to the similarity and quantity of the documents, respectively. The color at the top of each hill indicates different levels of internal SD, with red representing low internal SD and blue representing high internal SD. None of the 4 hilltops displayed blue, indicating a low internal SD (Figure 14A).

The specific clustering results were as follows: cluster 0 included 7 keywords, focusing on the application of traditional algorithms in diagnosis and classification (sensors, diseases, task analysis, feature extraction, machine learning, classification, and support vector machine); cluster 1 included 5 keywords, centered on wearable technologies and digital health (digital health, wearable device, accelerometer, gyroscope, and inertial sensors); cluster 2 included 6 keywords, focusing on deep learning–driven gait monitoring and freezing analysis (gait analysis, inertial measurement unit [IMU], wearable sensors, FOG, CNN, and deep learning); cluster 3, the largest cluster, included 8 keywords, focused on remote diagnosis and intelligent monitoring of movement disorders (bradykinesia, PD, tremor, essential tremor, movement disorders, gait, smartphone, and telemedicine; Figure 14B).

### Keyword Progress Analysis

The keyword theme progression reveals a logical advancement in PD research, spanning across technology, symptom understanding, and device development. This development's deepening and diversification became particularly evident after 2015. The research techniques have evolved from basic signal processing to advanced machine learning models. Initially, the focus was on extracting and cleaning signals from sensor data, emphasizing how to effectively separate useful data. Subsequently, statistical and dimensional reduction methods, such as principal component analysis, were used to explore the most representative features in high-dimensional data, reducing computational complexity and enhancing analytic efficiency. More recently, supervised and unsupervised learning, especially deep learning (eg, CNN), has been used to automatically extract complex data patterns, significantly improving the ability to recognize and predict the diverse symptoms of PD.

**Figure 15:**
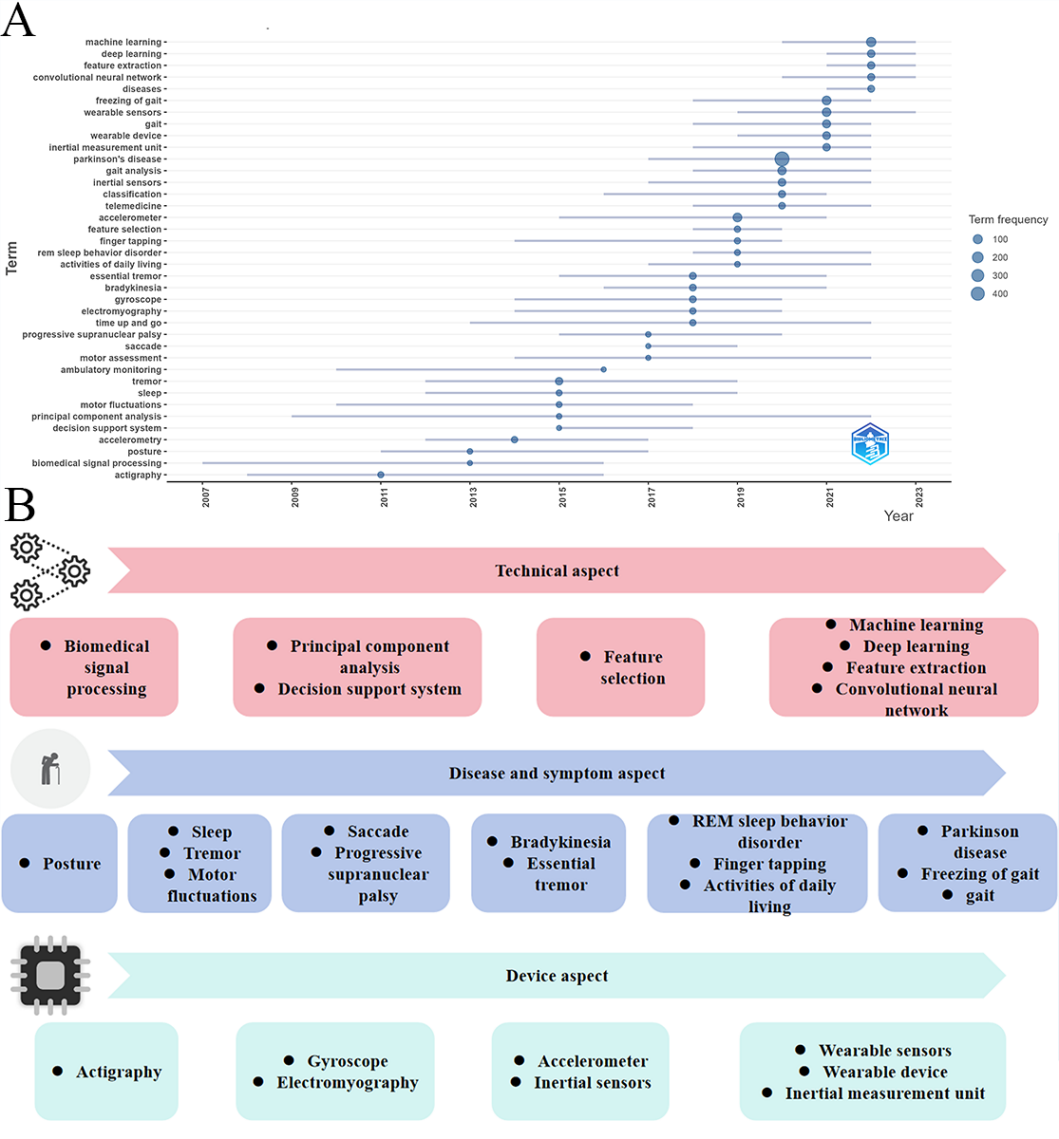
Theme trend changes. (A) Annual theme trend changes. (B) Development of Parkinson disease digital biomarkers in technology, symptoms, and devices. REM: rapid eye movement.

Initial research on PD focused on basic motor functions, such as posture, motor fluctuations, and tremor. These symptoms are often early manifestations of the disease and can be easily detected through surface observation and simple devices. As research progressed, more complex motor abnormalities, such as saccades, and rarer disease features, like progressive supranuclear palsy, began to be explored. Eventually, the research expanded to include additional indicators related to quality of life and disease progression, such as REM sleep behavior disorder (RBD) and FOG, emphasizing comprehensive monitoring and intervention for patients’ overall well-being. Data collection has evolved from simple recording tools to the combination of various sensors, enabling more precise and multidimensional data capture. Early devices, such as activity monitors (actigraphy), were among the first tools used to monitor patients’ movements and activities, recording sleep and activity states. As the demand for high-quality data increased, inertial sensors (eg, accelerometers and gyroscopes) were integrated into these devices, allowing for more detailed tracking of movement trajectories, particularly in applications involving complex gait abnormalities. Finally, integrated devices, such as wearable devices and IMUs were developed, capable of simultaneously capturing multimodal data, enabling real-time monitoring and complex analyses, and providing data support for personalized interventions. This provides valuable data support for personalized interventions (Figures 15A and 15B).

### Review of Hot Topics

**Figure 16:**
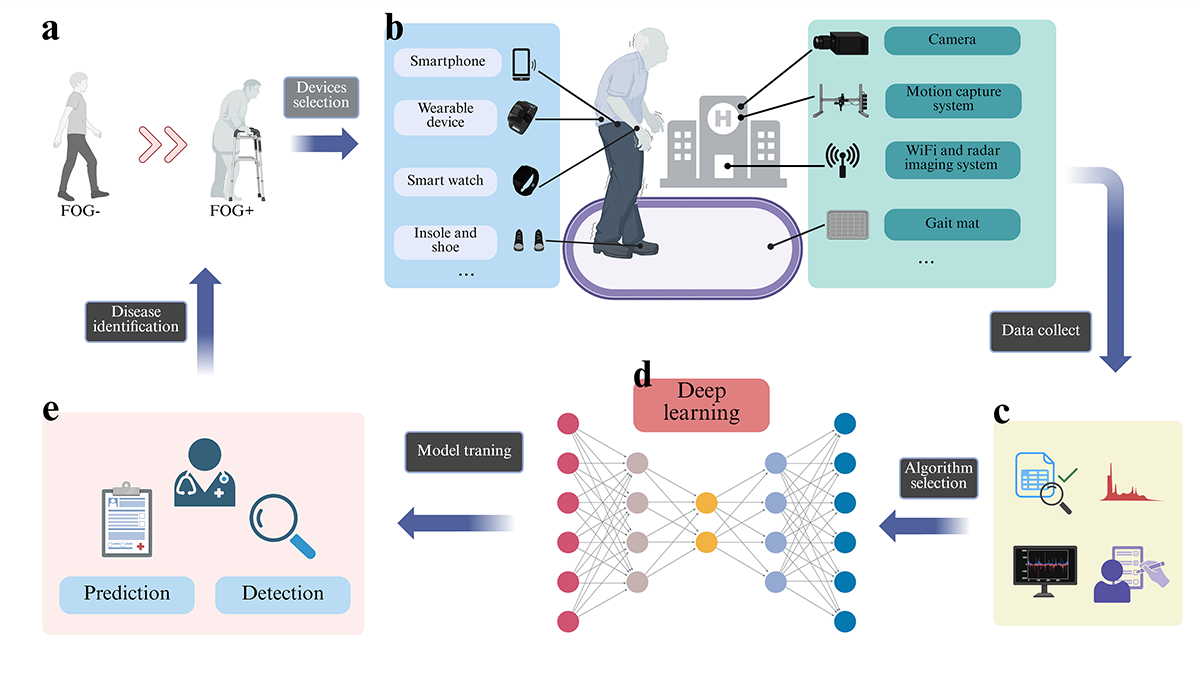
The entire process of constructing a deep learning model for Parkinson freezing of gait (FOG): (A) the occurrence of FOG; (B) selection of digital devices; (C) data collection and analysis from digital devices; (D) application of deep learning and neural networks; (E) training of the model and output of results.

In the aforementioned bibliometric analysis, through topic analysis, keyword emergence, and high-frequency keyword clustering, we identified that deep learning has emerged as a prominent trend in the diagnostic application of digital biomarkers for PD in recent years. Consequently, we further focused on the application of deep learning in constructing Parkinson FOG prediction models and presented a comprehensive review. Figure 16 illustrates the entire process of building a deep learning model for Parkinson FOG.

### Main Characteristics of the Included Articles

**Figure 17:**
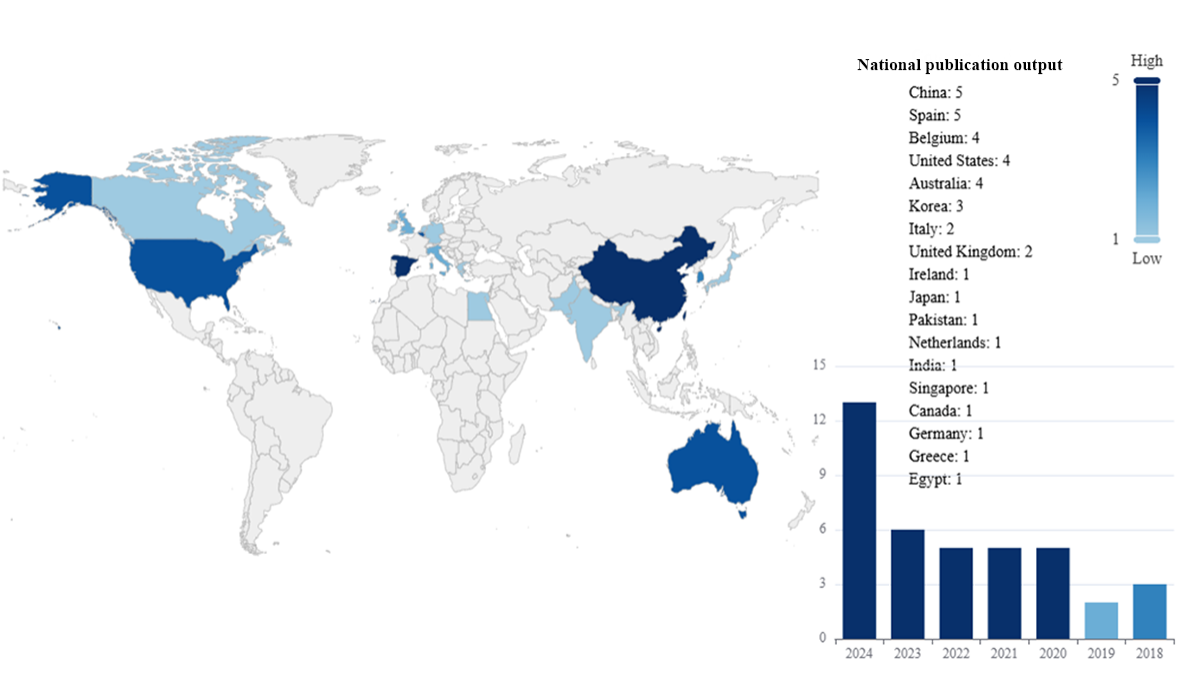
Geographic and time-zone distribution of the output of deep learning models for Parkinson freezing of gait.

A total of 40 studies were included for analysis, with publications spanning from 2018 to 2022. Nearly half (19/40, 48%) of these studies were published in 2023 or later. This trend indicates the growing attention on deep learning in the diagnostic detection of Parkinson digital biomarkers in recent years. The included studies were conducted in 19 different countries, with notable contributions from China, publishing 5 (13%) studies, while 10 countries published only 1 (3%) study each (Figure 17).

Among these studies, the number of participants ranged from 4 to 139, with only 8 (20%) studies involving more than 50 participants. In total, 32 studies reported the number or proportion of FOG events collected or used, covering a range of 56 to 1558 events, with only 5 (13%) studies explicitly stating the use of more than 1000 FOG events. Moreover, some studies (18/40, 45%) used public datasets for analysis, with 3 (8%) studies using 3 different datasets [60-62]. In studies that independently recruited participants, the largest sample size was 120, with one study [63] enrolling 80 patients with PD and collecting 1450 FOG events. Regarding the nature of the model tasks, 32 (80%) studies focused on FOG detection, 5 (13%) studies on FOG prediction [64-67], and 3 (8%) studies on both FOG prediction and detection [61,68,69]. The general information for these studies is presented in Table 1, with specific details in Multimedia Appendix 9 [60-99].

**Table 1:** General information on deep learning models for freezing of gait (FOG).

Item			Articles, n		References
**Research purpose**					
	Detection	32		[60,62,63,70-98]	
	Prediction	5		[64-67,99]	
	Detection and prediction	3		[61,68,69]	
**Participants, n**					
	≤10	13		[64,65,67,68,73,82,84,86,90,92,94,95,98]	
	11-99	24		[60,62,63,66,69-72,74-76,78-81,83,85,87,88,91,93,96,97,99]	
	≥100	3		[61,77,89]	
**Number of FOG events**					
	≤99	1		[85]	
	100-999	13		[64-66,68-70,81-83,87,90,94,95]	
	≥1000	5		[60,61,63,80,93]	
	Not specifically reported	13		[62,71,77-79,84,86,88,91,92,96-98]	
	Not reported at all	8		[67,72-76,89,99]	
**Database**					
	Self-collected	22		[63,66-71,73-79,81,82,84,89,91-93,96]	
	Daphnet	9		[60,62,64,65,86,90,94,95,98]	
	Rempark	5		[60-62,80,88]	
	Oday	1		[60]	
	Kaggle	2		[72,99]	
	ADL^a^	2		[61,62]	
	6MWT^b^	1		[61]	
	Mocap	1		[83]	
	Dataset constructed	1		[85,100]	
	Cupid-IMU^c^	1		[87]	
	MASPARK^d^	1		[97]	

^a^ADL: activity of daily living.

^B^6MWT:6-minute walking test.

^c^IMU: internal measurement unit.

^d^MASPARK: project Improving Quality of Life with an Automatic Control System.

### Data Collection Characteristics

Among the 22 studies that involved self-collected data, a variety of data collection devices were used. Specifically, 5 (23%) studies used pressure-sensing insoles or systems [69,73,78,79,84], 2 of which were nonwearable devices [78,79]. In total, 2 (9%) studies used motion capture systems [63,74], with one using a nonwearable optical capture system [74]. Moreover, 3 (14%) studies used WiFi devices [76,89,93], 3 (14%) studies involved cameras [75,78,91], 1 (4%) study used a smartphone [96], 1 (4%) study incorporated electroencephalogram devices [67], and 13 (59%) studies relied on wearable devices for data collection. Among the 18 studies that used public datasets, 1 (5%) study used data from wearable IMU and smartphones [62], 1 (5%) study used commercial smartwatch data [87], 2 (11%) studies used motion capture system data [83,85], and most (14/18, 78%) of the remaining studies relied solely on data from wearable devices. Details on the placement of these devices and the specific tasks involved in data collection are provided in Table 2 and Multimedia Appendix 9.

Both gait data from datasets and those collected independently required further segmentation for formal analysis. Most studies used either fixed or sliding window segmentation methods. Specifically, 20 (50%) studies used sliding window segmentation, 13 (33%) used fixed window segmentation, 1 (3%) used frame-by-frame segmentation (where the gait data were temporally segmented using frame-by-frame annotation) [83], 1 (3%) study used adaptive window segmentation (with window lengths ranging from 0.3 to 3 s) [77], and 5 studies (13%) applied event-based segmentation methods [69,85,92,93,99]. Overall, the time window lengths across these studies ranged from 0.128 seconds to 10 seconds. A summary of the gait data segmentation methods and their main contributions in each study is provided in Table 2 and Multimedia Appendix 10 [60-99].

In addition, no studies have yet reported the use of privacy protection strategies, such as differential privacy, homomorphic encryption, or federated learning optimization. In total, 6 (15%) studies compared their results with those of medical professionals and provided specific intraclass correlation coefficients (ICCs), demonstrating high consistency with expert annotations [70,71,75,77,82,83]. For instance, one study using a multistage temporal convolutional network achieved ICCs of 0.92 for percentage of freezing time and 0.95 for the number of freezing events [70]. Another study using a deep CNN model reported ICCs of 0.93 for percentage of freezing time and 0.95 for the number of freezing events [82].

**Table 2:** Device data collection and analysis for deep learning models of freezing of gait in Parkinson disease.

Item		Articles, n	References
**Device for data collection**			
	Wearable devices	29	[60-73,77,80-82,84,86-88,90,92,94,95,97-99]
	Nonwearable pressure sensing systems	2	[78,79]
	Nonwearable motion capture systems	3	[74,83,85]
	Cameras	3	[75,78,91]
	WiFi and radar systems	3	[76,89,93]
	Smartphones	2	[62,96]
**Gait segmentation method**			
	Sliding window	20	[60-63,65,66,68,70-72,81,82,84,87,88,90,94-96,98]
	Fixed window	13	[64,67,73-76,78-80,86,89,91,97]
	Event-based segmentation	5	[69,85,92,93,99]
	Other	2	[77,83]
**Gait segmentation duration**			
	≤1 s	9	[64,66,67,75,78,79,84,86,91]
	1-5 s	19	[60-63,68,70-72,76,80-82,87,88,90,94-98]
	≥5 s	1	[74]
	Multiple time divisions	4	[65,70,77,89]
	Undefined duration	7	[69,73,83,85,92,93,99]

### Data Types and Multimodal Data Fusion

In the research on digital biomarkers, different types of data are often integrated through information fusion strategies to enhance model accuracy and robustness. Multimodal fusion strategies are typically categorized into 4 types: early fusion, midlevel fusion, late fusion, and hybrid fusion [101]. However, only a few (3/40, 8%) studies have explored the fusion of different types of data. Among them, 3 (8%) studies used different fusion approaches: one utilized early fusion of radar and wireless signal data [89], another adopted intermediate fusion of video and pressure sensor data [78], and the third implemented late fusion of neuro-sensor and inertial sensor data [67]. Moreover, research focusing on unimodal data was more common. For instance, 26 (70%) studies used inertial sensor data, indicating the widespread use of this sensor in motion analysis and action recognition. Other sensor data were used less frequently, with 4 (11%) studies based on pressure sensor data, 3 (8%) studies using optical motion capture sensor data, and 2 (5%) studies each using video and wireless signal data. Detailed information is provided in Table 3. The performance range for different types of models is provided in Multimedia Appendix 11 [60-99].

**Table 3:** Modal data and fusion methods in deep learning models for Parkinson freezing of gait.

Modal data and fusion methods			Articles, n		References
**Unimodal research**					
	Inertial sensor data	26		[60-66,68,70-72,77,80-82,86-88,90,92,94-99]	
	Pressure sensor data	4		[69,73,79,84]	
	Optical motion capture sensor data	3		[74,83,85]	
	Video data	2		[75,91]	
	Wireless signal data	2		[76,93]	
**Multimodal research**					
	Video data and pressure sensor data	1		[78]	
	Neural sensor data and inertial sensor data	1		[67]	
	Radar sensor data and wireless signal data	1		[89]	
**Modality fusion**					
	Early fusion	1		[89]	
	Midlevel fusion	1		[78]	
	Late fusion	1		[67]	

### Characteristics of Deep Learning Model Techniques

Among the studies included, 31 (78%) studies identified the optimal model as a CNN or CNN-based architecture, 1 (3%) study used graph fusion network [78], 1 (3%) study used recurrent neural network (RNN) [76], and 3 (8%) studies used a subtype of RNN, long short-term memory (LSTM) [69,86,92]. Due to differences in data processing capabilities and model complexity across algorithms, selecting an appropriate classifier is crucial for the reliability of research findings. To address this, 29 (73%) studies constructed multiple models for comparison to identify the best deep learning model. After comparison, 23 (58%) studies selected CNN-based architectures as the best model. In total 2 (5%) studies combined CNN and RNN to leverage both spatial and temporal patterns, where the CNN layers were used to extract feature maps, and the RNN layers were used to model the temporal dependencies of these features [88,90]. In addition, 2 (5%) studies used RNN [76,91], and 1 study chose LSTM [92].

### Performance of Deep Learning Models in Digital Biomarker Studies

In the 17 studies comparing nondeep learning algorithm models, one study [99] found that the best model, decision tree, outperformed deep learning models, achieving an accuracy of 91%. Support vector machine and random forest were the most commonly used traditional algorithm models. Among these, the accuracy of the 9 random forest models ranged from 0.80 to 0.90, sensitivity ranged from 0.71 to 0.95, specificity ranged from 0.63 to 0.89, and the area under the curve (AUC) ranged from 0.80 to 0.96. The accuracy of the 8 support vector machine models ranged from 0.79 to 0.86, sensitivity ranged from 0.62 to 0.86, specificity ranged from 0.76 to 0.90, and only one study reported an AUC of 0.88.

**Figure 18:**
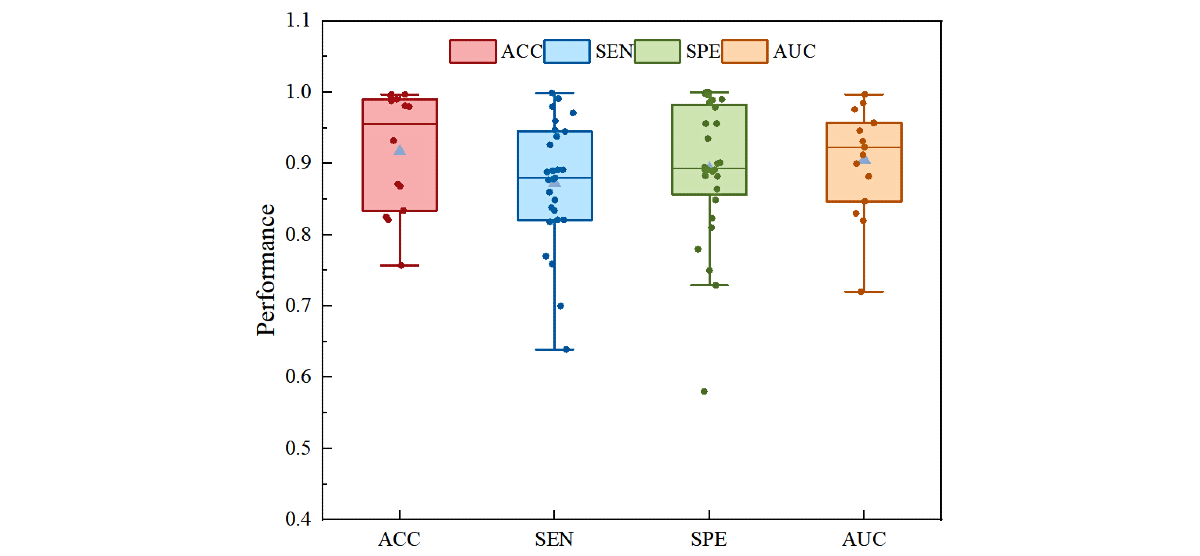
Performance distribution of deep learning models for freezing of gait in Parkinson disease. ACC: accuracy; AUC: area under the curve; SEN: sensitivity; SPE: specificity.

Due to the objective differences in research methods and feature types, we did not directly compare the performance of the studies but instead presented and described the distribution of deep learning model performance in digital biomarker research. Specifically, 20 (50%) studies did not report accuracy, 11 (28%) studies did not provide sensitivity data, 10 (25%) studies did not report specificity, and 25 (63%) studies did not provide AUC values. In detection models, all models had an accuracy >0.75, with an average of 0.92. For sensitivity, only 4 models had a performance below 0.8, with an average of 0.88. For specificity, only 4 models had a performance below 0.8, with an average of 0.90. Regarding AUC, only one study had a value lower than 0.8, with an average of 0.91. The specific performance of each detection model is shown in Figure 18.

For prediction models, 5 (63%) studies reported the lead time for predictions. In addition. 2 (25%) studies [64,65] predicted FOG events approximately 2 seconds ahead. One multihead CNN model was able to predict 52.3% of FOG events ahead of time, with an average lead time of 3.1 seconds, and achieved 87.7% sensitivity and 88.3% specificity [49]. In contrast, another CNN model predicted only 21.9% of events ahead of time but was able to detect FOG events within 2 seconds of onset, reducing the detection delay to 0.1 seconds [68]. The earliest prediction model successfully predicted FOG events approximately 7.17 seconds ahead of time, with a 77.27% success rate for predictions made at least 3 seconds before FOG onset and achieved 75.33% accuracy in sample-level classification [66]. In addition, the integrated model of 2 neural networks, EEGFoGNet and IMUFoGNet, combines electroencephalogram and IMU data, achieving a prediction accuracy of 92.1% with a 1-second prediction window [67]. These models provide crucial support for clinical and personal interventions. The specific performance of all models is provided in Multimedia Appendix 12 [60-99].

### Feature Selection

In the feature selection process, the lack of a unified standard and the variety of feature types generated during measurements remain major challenges in digital biomarker research for FOG in PD. In addition, differences in digital biomarker types, measurement devices, and experimental paradigms lead to inconsistencies in data collection, presenting additional difficulties for researchers. All studies reported the features used, with nearly all of them relying on the autonomous feature learning capabilities of neural networks for feature input. In total, 3 (8%) studies used a hybrid approach that combined manual feature engineering with neural network-based feature learning [65,80,94]. Regarding the transparency of autonomously learned features, only one study used the Shapley Additive Explanations method to improve model interpretability [60]. In addition, one study used expert prior knowledge for feature selection and reported feature importance scores calculated in MATLAB, though no further details were provided [77].

### Sample Imbalance and Missing Data Handling

During model training, sample imbalance can prevent the model from effectively learning the features of each group. Among the studies included, 12 (30%) reported and used sample balancing techniques. Specifically, 3 (%) studies used synthetic minority oversampling technique [62,65,99], 5 (8%) studies used data augmentation methods [68,73,86,90,97], 2 (5%) studies adjusted class weights [63,82], 1 (3%) study used both data augmentation and undersampling [72], and 1 (3%) study reported using oversampling but did not provide further details on the method used [74]. Due to various challenges in the data collection process of digital devices, researchers often face the issue of missing data in datasets. Using appropriate handling methods can improve the accuracy of the results. Despite this, only 2 (5%) studies reported the methods used for handling missing data, using zero imputation [74] and listwise deletion [99]. Detailed information on sample balancing and missing data handling methods in each study is provided in Multimedia Appendix 13 [60-99].

### AI Model Validation

The discriminative ability of the model can be assessed through internal or external validation [102]. However, in the study of Parkinson digital biomarkers for FOG, only 4 (8%) studies performed both internal and external validation, using different datasets for each validation [60-63]. Regarding internal validation, most studies used methods suitable for small sample sizes. Specifically, 17 (33%) studies used k-fold cross-validation, 15 (29%) studies chose leave one out cross-validation, 1 (2%) study used a more stringent nested cross-validation, and 12 (24%) studies used hold out validation. Model calibration is crucial for evaluating predictive performance, but no studies used calibration plots or the Hosmer-Lemeshow test to assess model calibration. Detailed information on the validation methods used in each study is provided in Table 4 and Multimedia Appendix 13.

**Table 4:** Validation methods for deep learning models of freezing of gait in Parkinson disease.

Validation approach			Articles, n		References
**Internal validation**					
	Leave one out cross-validation	16		[65,67-71,80,82,83,85,87,88,90,94,95]	
	K-fold cross-validation	17		[61,63,64,66,67,72,75,77,81,87,89-91,94-97]	
	Hold out method	12		[60,62,72,73,76,78,84,86,92,93,98,99]	
	Random repeated sampling validation	1		[74]	
	Nested cross-validation	1		[79]	
**External validation**					
	Cross-dataset validation	4		[60-63]	

### Reproducibility and Reporting Standards

In studies involving AI prediction models, the TRIPOD (Transparent Reporting of a Multivariable Prediction Model for Individual Prognosis or Diagnosis) guidelines should be followed, and code should be made publicly available to enhance reproducibility. However, among the 39 studies, none explicitly acknowledged compliance with the TRIPOD guidelines, and only 5 (13%) studies made their research code or coding pathways publicly available [60,70,82,83,97]. This undoubtedly impacts the reliability and applicability of these predictive models in the medical field. Further details are provided in Multimedia Appendix 13.

## Discussion

### Overview

This study combined bibliometric analysis and scoping review methods to provide a comprehensive overview of the multifaceted landscape of digital biomarkers in PD research. Specifically, we revealed the growth trends in research output, reflecting the increasing prevalence of digital biomarkers in recent years. Furthermore, we identified challenges in interdisciplinary collaboration, as well as weak cooperation between countries and institutions, and examined the geographic and institutional distribution of funding, offering recommendations for future development in the field. Finally, we summarized several key research hot spots and conducted a scoping review of deep learning models for Parkinson FOG, reporting on the algorithms’ architectures and performance. This review highlights the potential of digital devices and deep learning models in detecting and predicting Parkinson FOG. In the following sections, we will discuss these findings in detail.

### Main Findings From the Bibliometric Analysis

#### Continuous Increase in Output

Since 2015, the research trend on digital biomarkers for PD has shifted. This trend is closely linked to the accumulation of previous high-value studies, which have laid a solid foundation for subsequent work. In our analysis, 3 highly cited studies from 2014 provided timely references for future research. Among them, Microsoft’s Kinect sensor was validated as an effective tool for remotely monitoring motor symptoms in patients with Parkinson, prompting subsequent studies to shift focus toward the application of portable inertial sensors [50]. In addition, a gait spatiotemporal parameter estimation method based on bilateral ankle magneto-IMUs was successfully validated for its effectiveness in detecting gait events and estimating gait parameters in patients with Parkinson [53]. Furthermore, continuous sensor wear successfully predicted the time of the first fall in patients with Parkinson who had not previously fallen [54]. These studies provided important insights and laid the groundwork for the development of digital biomarkers, especially in the areas of motor symptom monitoring and fall prediction. Moreover, we observed that the number of multidisciplinary researchers involved in this field has remained stable, with the research focus gradually diversifying, indicating that an increasing number of researchers are now focusing on the exploration of digital biomarkers for PD.

Currently, research on digital biomarkers in Parkinson diagnosis and assessment is steadily growing, a shift likely driven by technological advancements and changes in conceptual frameworks. The widespread application of big data and cloud computing technologies has enabled researchers to process and store large-scale datasets, significantly accelerating the broader application and deeper investigation of digital biomarkers. Ongoing advancements in digital devices, such as wearable devices and sensors, provide higher-quality and more granular data sources [103], enabling a more accurate portrayal of behaviors of interest in both clinical and research settings [104]. Progress in machine learning and AI has made the processing of high-dimensional data more efficient and feasible [105]. Furthermore, cost-effective and noninvasive methods are gaining widespread attention compared to traditional diagnostic approaches, further driving the growth of digital biomarker research [106]. The development of these methods has enabled the data collected to be used for predicting disease progression, assessing drug efficacy, and even adjusting treatment plans. It is anticipated that this field will yield even more research outcomes in the future.

#### Insufficient Interdisciplinary Collaboration

As the field gradually matures, researchers from different disciplines have started to contribute to the area. Currently, researchers with a background in neurology are the primary contributors, but an increasing number of engineers, particularly those from computer science and communications engineering, are starting to focus on this topic. However, it is important to note that participants from multiple disciplinary backgrounds remain relatively few, and interdisciplinary collaboration is still limited. The collaboration network among core authors often exhibits internal biases. This may be related to the challenges of knowledge transfer across disciplines. To strengthen interdisciplinary collaboration, the FDA is establishing a network of digital health experts, focusing on applications of physiological sensors, wearable devices, the medical internet of things, and digital biomarkers [107]. In addition to efforts by government agencies, team structures should be adjusted according to specific research objectives. For example, the Delphi method could be used to determine evaluation criteria and prioritize measurement indicators [108]. Neuroscience, as the primary recruiting discipline, can offer more opportunities for communication through cross-disciplinary academic conferences, webinars, and other platforms. Engineering typically plays an outward-facing role by introducing wearable technologies and AI to drive innovation in health care. Particularly in digital biomarker research, the integration of technological innovation and scientific theory will significantly enhance both the depth and applicability of research.

#### Lack of National and Institutional Collaboration

A total of 61 countries have participated in research, reflecting broad international interest. The United States has long been a dominant player, with numerous European countries also showing interest in this field. In recent years, China has demonstrated a strong interest in this topic, with research output increasing significantly in a short period, highlighting the regional differences in attention to this area. The collaborative network in the research also shows significant geographic disparities. The United States has established connections with 36 different countries, while other high-output countries still have ample room to expand and strengthen multinational exchanges and cooperation. The establishment of open data-sharing platforms could facilitate this collaboration, such as creating digital device databases similar to the FDA’s Medical Device Database [109] or multicenter datasets like the Parkinson’s Progression Markers Initiative [110].

In the analysis of institutional composition in high-output countries, universities are generally the most common institutions involved, while companies are the least represented and have weaker collaborations with other types of institutions. This indirectly reflects the relatively weak or still emerging connection between research and commercialization. In the practical application of digital biomarkers, companies are not only responsible for the development and production of hardware devices, such as portable sensors and wearable devices, but also drive the implementation of clinical practices by offering innovative algorithm optimization solutions and data analysis tools. Meanwhile, close collaboration between research institutions and companies is crucial. Basic research and algorithm innovations in academia provide technical support for companies, while companies, with their extensive market experience and resources, play a vital role in accelerating the commercialization and productization of technologies. It is estimated that the digital biomarker market will reach US $35.8 billion by 2035 [111]. As the FDA advances its software as a medical device program [112], the role of companies in supporting, commercializing, and promoting digital biomarker technologies will be further amplified, emphasizing the need for enhanced collaboration between companies and academic institutions, particularly those aiming to deploy these digital devices for large-scale data collection.

#### Global Funding Models and Priorities

Funding models reflect the current hot topics and priorities in scientific research. In the past, the challenges in traditional drug development in neurology led to lower expected returns, which indirectly contributed to digital health and precision medicine becoming significant investment directions within the field of neurology [113]. However, different regions have distinct strategic focuses and resource allocations in Parkinson digital biomarker research. In the United States, funding agencies, such as the NIH and the Michael J Fox Foundation, cover multidisciplinary research directions, especially the integration of digital technologies, medicine, and health care services. The Fox Wearable Companion app developed by the Michael J Fox Foundation has demonstrated high adherence in monitoring Parkinson progression [114], and it has also funded various data collection initiatives for digital tools [115]. In contrast, European funding tends to be more focused on engineering and development. The European Union funds more research in the fields of science and technology, as well as computer science, while the Italian Ministry of Health and Parkinson’s UK have a particular emphasis on engineering, having developed several innovative tools for gait, tremor, and mental health monitoring [116]. This multifaceted funding approach helps the development of digital biomarkers from research to deployment. Furthermore, this information provides strategic guidance for researchers seeking funding, offering insight into key areas of focus and application.

#### Diversification of Disciplines in Publications

The published research on Parkinson digital biomarkers exhibits a diverse nature. Although most researchers in this field come from a neurology background, the variety of disciplines in which these studies are published, as well as the clustering of cocited journals, reveal a broad range of findings, which is a positive development. This reflects the interdisciplinary collaboration and innovation potential in the field, indicating that researchers from various backgrounds are approaching the problem from different perspectives, thus advancing the field. Moreover, we encourage publishing Parkinson digital biomarker research not only in neuroscience-exclusive journals but also in journals from diverse fields. Such practices will foster interdisciplinary knowledge transfer and potential collaborations, bringing new ideas and solutions to a wider range of domains.

#### Hot Spots and Trends in Parkinson Digital Biomarker Research

Through keyword clustering, burst analysis, and evolution-based combined analysis, we identified several research hot spots and trends as follows: (1) diagnostic classification using AI (primarily based on traditional machine learning algorithms), (2) remote diagnosis and monitoring of motor disorder digital biomarkers, (3) comprehensive analysis of RBDs, and (4) deep learning models for FOG.

These 4 hot spots cover key aspects of PD, including the technological dimension (machine learning and deep learning), the motor symptom dimension (remote diagnosis and monitoring of motor disorder digital biomarkers), and the nonmotor symptom dimension (comprehensive analysis of RBDs).

#### Application of Traditional Machine Learning Algorithms

Although our keyword burst analysis indicates that deep learning has emerged as a prominent term in recent years, traditional algorithms remain a major research focus in the field. This is mainly because, compared to neural networks, traditional algorithms often provide more mechanistic insights and interpretability. For example, in decision trees, each branch and node have clear physical significance [117]; random forests can quantify the contribution of each feature to the overall model’s prediction, thus assessing feature importance [118]; and the kernel function choice in support vector machines directly influences the decision boundary and classification performance [119]. Furthermore, nonneural network methods generally require fewer training data, which is crucial for applications where the sample size is small or data are noisy, as in digital device data collection scenarios, and they also incur lower computational costs [120].

In contrast, traditional methods (such as threshold-based detection methods) have limitations due to the limited generalizability of manually designed features. As the number of sensors and parameters increases, it becomes increasingly difficult to use manually set thresholds for FOG detection [121]. Therefore, using machine learning algorithms to handle larger and more complex data has become key to improving analysis performance and accuracy. For example, Rios-Urrego et al [122] used the k-nearest neighbors algorithm in conjunction with kinematic, geometric, and nonlinear dynamic features captured by a digitizer, achieving an 83.3% classification accuracy between patients with Parkinson and healthy individuals. In another study using a noncontact gesture and hand-tracking device to capture the 3D coordinates of the palm center and fingertips, the bagged tree algorithm was used to classify tremor severity in PD, achieving an accuracy of 98% [123]. In addition, models built using sensor data from gait, accelerometers, gyroscopes, and magnetometers have also performed well [124-127]. Therefore, continuing to use traditional algorithms in various digital devices remains an effective and promising approach.

#### Remote Diagnosis and Monitoring of Movement Disorders

Remote monitoring and diagnosis represent key application areas for digital biomarkers, particularly as the lessons learned from the COVID-19 pandemic have underscored the importance of remote health management in global health care systems [128]. This shift marks a significant transition from active data collection to passive data collection. However, under such conditions, widespread monitoring of certain nonmotor symptoms remains challenging. For instance, monitoring constipation relies on technologies such as smart pills [129], abdominal skin electrical activity [130], or smart toilets [131]. These methods are far more complex and difficult to deploy remotely compared to wearable devices, necessitating the development of more advanced technologies. In addition, commercially available heart rate variability sensors still exhibit significant accuracy discrepancies and error rates compared to the gold standard, electrocardiography [7], which affects the reliability of their results. Monitoring nonmotor symptoms, such as mood and autonomic dysfunction, is also not ideally suited for detection through home-based embedded sensors or wearable devices.

In contrast, Parkinson motor symptoms have distinct advantages in both their diversity and suitability for remote collection. Symptoms such as gait abnormalities, FOG, tremors, and manual tasks are core features assessed by the Movement Disorder Society-Unified Parkinson’s Disease Rating Scale and have demonstrated high accuracy and sensitivity in data collection using various wearable devices and sensors [20,132]. These technologies are also portable and cost-effective. The application of advanced motion analysis techniques has further minimized the impact of laboratory effects. For example, in smart home environments, embedded sensors can continuously capture motion in natural settings, with accuracy and ecologic validity often surpassing that of laboratory conditions [133,134]. The use of gait shoes enhances the objectivity and reliability of motion quantification [135], providing richer pressure data [84]. Moreover, with the widespread use of sensors in devices, such as smartphones, not only is it easy to acquire motion data from accelerometers and gyroscopes, but cameras can also be used for visual analysis, further improving diagnostic accuracy. The collection of data through applications and keystroke functions is also straightforward. When combined with cloud platform analytics, these technologies can automatically record and process data with minimal user intrusion, contributing to high user acceptance [136,137]. Therefore, remote diagnosis and monitoring of movement disorders not only attracts significant attention but also holds considerable promise for the future.

#### Analysis of RBD

RBD is among the most promising symptoms for developing digital biomarkers, as sleep disturbances are one of the most common nonmotor symptoms in PD [138]. In addition, abnormal physical movements during the REM sleep stage significantly impact patients’ quality of life [139]. Traditionally, the diagnosis of RBD has relied on polysomnography (PSG) as the gold standard. However, the high cost and inconvenience of PSG have driven the development of wearable and portable devices, which may explain why multidimensional sleep monitoring is a fundamental theme in topic analysis. During sleep, the integration of sensors, such as smartwatches with machine learning algorithms can effectively differentiate the sleep stages of patients with PD and detect abnormal REM behaviors [140]. Furthermore, devices like electroencephalogram headbands [141], actigraphy monitors [142], and electromyography sensors [143] have also been shown to effectively identify RBD. Sleep features based on accelerometers, such as reduced sleep efficiency and turning speed, have been validated in multiple PD cohorts and are highly correlated with PSG [7]. Nevertheless, these devices still face accuracy and stability issues due to potential data loss during sleep, which need to be addressed. Notably, in predictive studies of RBD, tests conducted during nonsleep states also provide feasible approaches for the early identification of RBD. For instance, wearing inertial sensors can capture gait asymmetry [144] and prolonged preturn step time [145], which are warning signals of progression in patients with idiopathic RBD; tapping rhythm tests on tablets can reveal deficits in rhythm perception [146]; and changes in vocal features during microphone and telephone conversations are also considered potential biomarkers for identification [147,148]. These technologies not only help improve the sleep quality and overall quality of life for patients with PD but also play a crucial role in monitoring the progression of disease prodromes, such as idiopathic RBD, particularly by providing valuable data support in disease-modifying trials.

#### Gait Freezing Deep Learning Models

In the field of digital biomarkers for PD, deep learning has undoubtedly become a research hot spot in recent years and is regarded as a key trend for future development. However, we observe that current applications of deep learning are primarily focused on the issue of FOG. During walking, patients frequently experience brief episodes of “freezing,” which cause a temporary halt in their gait and may lead to safety risks, such as falls. Therefore, this paper not only provides a simple description of this emerging trend but also further explores the application and limitations of existing deep learning models in the detection of FOG.

### Main Findings of the Scoping Review

#### Model Selection

Recent studies on deep learning–based FOG detection and prediction can primarily be categorized into 2 main approaches: CNN and RNN.

A noteworthy observation is that only a few studies have integrated data from different sensor types for multimodal analysis. Most existing research relies on CNNs to process data from motion sensors, mainly because CNNs excel at handling spatially structured data. The information provided by motion sensors typically exhibits clear spatial features, such as gait and movement patterns. CNNs, through their local perception ability and multilayer convolution operations, can effectively extract these spatial features. In addition, CNNs can capture dynamic changes in time-series data, enabling effective recognition of the spatiotemporal evolution of gait changes, especially when dealing with gait-related time dependencies [149]. However, when integrating data from multiple sensors, more complex model architectures may be needed to fuse multimodal information, thus enhancing the overall predictive capability and diagnostic accuracy of the model.

RNN-based models, particularly LSTM networks, are well-suited for learning time-series dependencies, offering an advantage in causal prediction modeling [26]. LSTM networks excel at extracting temporal gait patterns and predicting the occurrence of FOG events [64]. Furthermore, studies based on transformer networks and attention mechanisms have demonstrated potential in FOG detection. The self-attention mechanism enhances the representation power of features, especially during the fusion and optimization of multimodal data [64], and by analyzing sequential windows of accelerometer data [80], it identifies the crucial data segments for FOG event detection, thereby improving the model’s ability to predict FOG events.

However, research on digital biomarkers generally lacks effective validation, particularly concerning the key identification features of FOG, which remain undefined. In addition, the automatic feature learning approach of deep learning partially hinders the advancement of this validation process. Although some studies have used Shapley Additive Explanations for local feature interpretation, without clearly defining the input features and their importance, it is challenging to effectively compare and validate digital biomarkers against the gold standard. Furthermore, feature selection is influenced by variations in devices or data collection tasks [150]. Therefore, future research should place greater emphasis on feature interpretation to enhance the reliability and clinical applicability of digital biomarkers.

#### Data Collection Methods and Device Diversity

In 22 studies, autonomous data collection methods were used, using a variety of digital device types. Wearable devices remain the mainstream choice; however, the introduction of pressure insoles, motion capture systems, and WiFi devices has enabled the capture of more detailed physiological and behavioral features, thereby enhancing the diversity and precision of digital biomarker research. Specifically, the use of pressure insoles and motion capture systems provides not only acceleration signals but also pressure data, joint positions, and movement trajectories [84,85]. WiFi devices offer the possibility of passive monitoring through noncontact methods. These approaches allow for more effective monitoring of patients’ motor states and gait variations, providing new perspectives for early diagnosis and treatment of the disease. However, the diversity of devices, while increasing the flexibility of data collection, also introduces challenges related to data standardization due to differences in resolution and sampling frequency. Future research should focus on normalization techniques and the establishment of unified data collection protocols to further enhance the adaptability and generalizability of models across different device data.

#### Limitations of the Dataset

When using publicly available datasets for research, other researchers can access the same datasets, allowing them to validate the experimental results. Currently, the most commonly used public datasets are the Daphnet dataset [151] and the Rempark dataset [152], which have relatively small sample sizes, containing 10 and 21 patients with PD, respectively. Deep learning models inherently rely on large sample sizes for training, so the limited number of samples may affect the model’s generalization ability and accuracy, potentially hindering the development of an effective model for detecting FOG in real-life scenarios. While some studies have addressed data insufficiency and sample imbalance through techniques such as data augmentation, these approaches do not resolve the fundamental issue of limited data. There have been attempts to diversify data sources by merging multiple datasets to mitigate the impact of limitations in individual datasets. However, this strategy may be hindered by discrepancies in data collection protocols, leading to lower data consistency. The field would greatly benefit from the creation of larger and more diverse public datasets that capture a broader range of FOG episodes and patient characteristics, ultimately enhancing research transparency and reproducibility.

#### Performance of Models

Our analysis indicates that the average accuracy of all detection models is 0.92, sensitivity is 0.88, specificity is 0.90, and the AUC is 0.91, demonstrating relatively excellent performance. However, identifying the optimal model remains challenging because the types and combinations of features significantly impact model performance, and there are still issues with the standardized reporting of existing model metrics. Future studies should systematically report these evaluation metrics, including specific values for AUC, accuracy, sensitivity, and specificity, to provide a more comprehensive perspective for comparing and improving model performance. Similarly, in predictive models, it is necessary to report the specific time windows for predictions.

#### Challenges in External Validation and Model Calibration

The lack of external validation and insufficient model calibration are 2 critical challenges in the development of AI models for Parkinson gait freezing. These issues not only raise concerns regarding the model’s generalizability across different environments but also limit its clinical applicability [153]. Currently, only 4 studies have conducted external validation, and most models fail to ensure calibration that aligns predicted probabilities with actual outcomes. This undermines the trust of clinicians and patients in these models for decision-making. Therefore, future research should focus not only on external validation of AI models but also on the development and reporting of model calibration methods to ensure their reliability and accuracy in clinical settings.

#### Issues With Code Transparency and Reporting Standards

In research on AI models for Parkinson gait freezing, the lack of code transparency and failure to adhere to reporting standards are 2 key issues that undermine the reproducibility of studies. Actively reporting adherence to established guidelines, such as the TRIPOD statement, and publicly sharing model code and data will facilitate the ability of researchers to verify and replicate the models. Particularly in the context of increasing diversity in digital biomarker research, where a greater variety of data will be generated, the portability of methods to process and acquire such data will become increasingly important. Therefore, future work should place greater emphasis on making model code and data publicly available to enhance validation and real-world applicability [154].

#### Challenges in Clinical Application

Although deep learning models demonstrate significant potential in the prediction and diagnosis of FOG, there are several challenges to successfully deploying these models in clinical practice. First, the accessibility and accuracy of data collection devices may vary across different clinical settings, potentially affecting the training and performance of the models [155]. Second, certain gait features critical for diagnosing FOG may require specialized sensors or equipment to capture. Furthermore, the high computational cost of deep learning model training may limit their application in resource-constrained clinical environments, although optimization techniques, such as quantization and compression, can partially alleviate this issue. Finally, successful clinical deployment not only requires robust technical support but also necessitates multistakeholder collaboration to address challenges, such as data privacy, ethical concerns, and improving model interpretability.

### Future Development Directions

AI, particularly deep learning, has demonstrated significant potential in the field of FOG. However, in digital biomarker research, clearly defining data collection and feature importance is crucial for clinical interpretation and practice. As data become increasingly complex and high-dimensional, explainable AI [156] and the effective identification of clinically relevant features emerge as urgent challenges. In addition, external validation of models remains essential and should not be overlooked.

Digital biomarkers are not a singular concept. The introduction of various digital devices has laid the groundwork for multimodal, multitype, and multisource sensor research. This combination of multimodal digital biomarkers not only offers personalized options but also enhances the precision of biomarkers. Furthermore, the integration with clinical omics data and other information is vital [157], further increasing their relevance in clinical applications.

Current research hot spots primarily focus on motor symptom biomarkers, especially FOG. With advancements in device technology, the dynamic monitoring of other motor symptoms, such as tremor, motor fluctuations, and eye movements, as well as the exploration of digital biomarkers from nonmotor symptoms, are becoming increasingly important directions.

**Figure 19:**
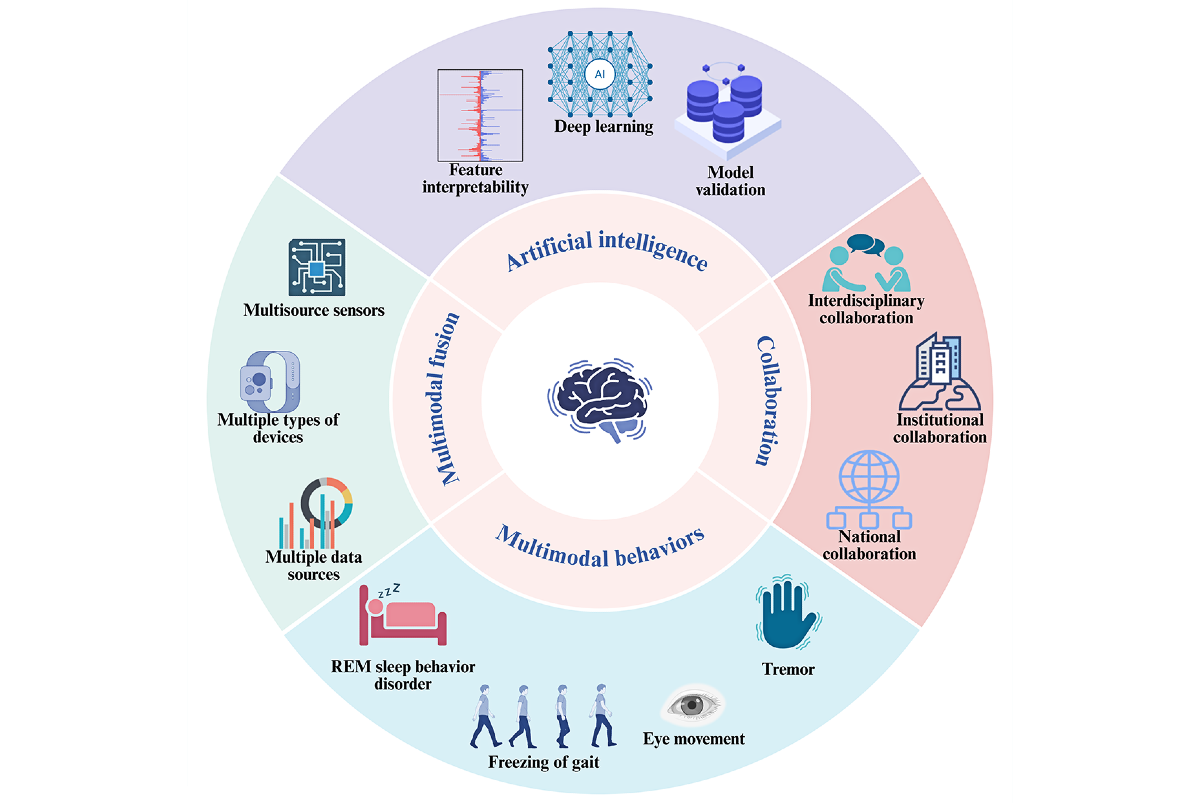
Future development directions in Parkinson digital biomarker research. REM: rapid eye movement.

Finally, the issue of insufficient collaboration in this field urgently needs to be addressed. Facilitating interdisciplinary, international, and interinstitutional collaboration to leverage strengths from multiple domains is critical for promoting the clinical translation and widespread application of innovative findings. Enhancing collaborative efforts has become a key focus to ensure the advancement and practical implementation of digital biomarker research. The 4 directions for future research are shown in Figure 19.

### Strengths and Limitations

To our knowledge, this study is the first to use bibliometric methods for a multidimensional comprehensive analysis of Parkinson digital biomarkers. Compared to many existing studies, we have enhanced the depth and breadth of analysis across various sections and conducted a detailed scoping review of emerging research hot spots. In addition, this is the first scoping review focused on deep learning models for Parkinson digital biomarkers. We have summarized the entire process of model development, covering various aspects from data collection to the public release of model code, providing a reference for the FDA’s open competition to identify and predict AI and machine learning models for detecting FOG events associated with PD.

However, this study has several limitations. Converting data formats to merge documents from multiple databases may have affected the accuracy of the results, as each database may possess different characteristics. Consequently, only one database was included, which might have led to the omission of a few related studies. Nonetheless, the WoSCC is considered the most representative, covering the most renowned and significant academic journals globally. Furthermore, our survey was limited to studies published in English, potentially overlooking high-quality research in other languages. Although this scoping review included 5 comprehensive databases, it did not incorporate conference proceedings, which may reduce the overall comprehensiveness of our study.

### Conclusions

In this study, we conducted a comprehensive analysis of Parkinson digital biomarker research using the WoSCC. Our findings revealed insights into publication output, countries, collaborations, funding, and other relevant aspects within this field. The United States played a pivotal role in advancing this area of research. We observed that disciplinary collaborations are primarily centered on neurology, yet there remains a need to strengthen interdisciplinary connections in the future. In addition, collaborations between industry and other institutions need to be enhanced.

Moreover, we summarized the deep learning models and algorithms used for detecting and predicting Parkinson FOG. Deep learning offers significant potential in this domain; however, future research must adhere to standardized protocols, enhance model interpretability, and perform external validations to ensure reliability and clinical applicability.

## Appendix

Multimedia Appendix 1 Bibliometric research search strategy.

Multimedia Appendix 2 Search strategy for the scoping review on freezing of gait.

Multimedia Appendix 3 PRISMA-ScR checklist for the scoping review on freezing of gait.

Multimedia Appendix 4 Bibliographic table for the cleaning of keywords related to Parkinson digital biomarkers.

Multimedia Appendix 5 The selection strategies and use methods of each tool.

Multimedia Appendix 6 High-output countries, authors, institutions, and funding sources table.

Multimedia Appendix 7 Highly cited journals, studies, and high-output journals information table.

Multimedia Appendix 8 Distribution of high-frequency keywords.

Multimedia Appendix 9 Basic information on Parkinson freezing of gait deep learning model research.

Multimedia Appendix 10 Data processing and key findings of Parkinson freezing of gait deep learning models.

Multimedia Appendix 11 Data types and multimodal data fusion.

Multimedia Appendix 12 Performance of Parkinson freezing of gait deep learning models.

Multimedia Appendix 13 Construction details of Parkinson freezing of gait deep learning models.

## Abbreviations

CNN: convolutional neural network
FOG: freezing of gait
ICC: intraclass correlation coefficient
IMU: inertial measurement unit
NIH: National Institutes of Health
PD: Parkinson disease
RBD: random eye movement sleep behavior disorder
REM: random eye movement
WoS: Web of Science
WoSCC: Web of Science Core Collection

## Data Availability

The datasets generated and analyzed during this study, as well as the code required for software operations, are available from the corresponding author upon reasonable request.
