# Supplementary material for: Digital Biomarkers for Parkinson Disease: Bibliometric Analysis and a Scoping Review of Deep Learning for Freezing of Gait
Source: J Med Internet Res. 2025 May 20;27:e71560. doi: 10.2196/71560 (PMC12134701; doi:10.2196/71560)
Supplement: Multimedia Appendix 1 [file jmir_v27i1e71560_app1.docx]

**Appendix 1. Bibliometric search query.**

| **NO** | **Item** | **Search query** | **Result** |
| --- | --- | --- | --- |
| **#1** | **Disease** | "Parkinson disease" OR "Parkinsonian disorders" OR parkinson* OR parkinsionism OR "parkinson's disease" | 174,762 |
| **#2** | **Digital biomarker** | "digital biomarker*" OR "digital marker*" OR "Diagnostic marker*" OR "Serial reaction time" OR "Sleep" OR "Sleep disturbances" OR "rapid eye movement sleep behavior disorder" OR "vocal biomarker*" OR "Speech biomarker* " OR "Conversation biomarker*" OR "acoustic features" OR "Facial movement" OR"facial movement*" OR "facial muscle activity" OR "Facial expression" OR "facial emotion"* OR "emotion*" OR "mood*" OR "apathy" OR "depression" OR "anxious*" OR "eye movement*" OR "ocular* movement*"OR "visual tracking" OR "saccade*" OR "fixation*" OR "pursuit movement*" OR "eye tracking*" OR "ocular motor control" OR "blink*" OR "pupil*"OR "eye blink*" OR "spontaneous blink*" OR "hand movement*" OR "hand motion*"OR "manual movement*" OR "finger tapping" OR "finger tracking" OR "fine motor skill*" OR "finger movement*" OR "finger motion*" OR "upper limb movement*" OR "upper limb motion*" OR "upper extremity movement" OR "arm movement*" OR gait* OR "gait analysis" OR "gait pattern*" OR walk* OR balance* OR "postural balance" OR "postural control" OR "postural stability" OR postur* OR "stability control" OR "body stability" OR "Heart Rate Variability" OR "heart rate" OR "heart-rate regulation" OR "freez* of gait" OR "gait freezing" OR tremor* OR trembl* OR "orthostatic hypotension" OR "hallucinat*" OR "delusion*" OR "olfactory dysfunction" OR "skin temperature*" OR "skin conductance" OR constipat* OR Constipation OR "olfaction" OR "sense of smell" OR "cognitive impairment*" OR "Cognitive Dysfunction" OR "Color Vision" OR pain OR "voice analysis" | 3,166,187 |
| **#3** | **Digital health** | "Digital Health Technolog*" OR "Digital Health*" OR "eHealth*" OR "mobile health" OR "mHealth" OR "mobile device*"OR "portable device*" OR "ingestible" OR " smart technolog*" OR "Haptic device*" OR "smartphone" OR "computer" OR "tablet" OR "smart watch" OR "smartwatch*" OR "Band" OR "ICT devices" OR "mobile phone*" OR "digital measure" OR "Sleep Monitoring" OR "Naturalistic driving" OR "Global positioning system" OR "wearable device*" OR "Driving behavior" OR "Sensor*" OR "inertial sensor*" OR "Inertia Measurement Unit" OR "inertial measurement unit" OR "tracker*" OR "keyboard" OR "Camera" OR "Touchscreen" OR "mobile applications" OR "smart home" OR "eye tracker" OR "eye-tracking device" OR "mobile EEG" OR "wireless EEG" OR "portable EEG" OR "wearable EEG" OR "VR" OR "Virtual Reality" OR "Augmented Reality" OR "AR" OR "smart pill*" OR "intelligent pill*" OR "digital pill*" OR "smart capsule*" OR "intelligent capsule*" OR "actigraphy device*" OR "actigraph*" OR "accelerometer*" OR "activity tracker*" OR "fitness tracker*" OR "smart mattress*" OR "intelligent mattress*" OR "smart shoe*" OR "intelligent shoe*" OR "smart footwear*" OR "electromyography" OR "EMG sensor*" OR "EMG recording*" OR "photoplethysmography" OR "PPG sensor*" OR "PPG monitoring*" OR "temperature sensor*" OR "PPG signal*" OR "thermal sensor*" OR "smart pill box*" OR "intelligent pill box*" OR "smart medication box*" OR "portable ECG" OR "portable electrocardiogram" OR "mobile ECG" OR "wearable ECG" OR "wearable electrocardiogram" OR "electrodermal activity" OR "psychophysiological signal*" OR "microphone*" OR "smart glasses" OR "remote monitoring device*" OR "voice assistant*" OR "digital assistant*" OR "biosensor*" OR "digital neurocognitive testing" OR "digital neurocognitive assessment" | 2,555,127 |
| **#4** | **Measure** | "detection" OR "screening" OR "screening test*" OR "computerised screening test*" OR "diagnosis" OR "evaluation" OR "assessment" OR "prediction" OR "clinical diagnosis" OR "early detection" OR "early diagnosis" OR "clinical application" OR "clinical utility" OR "diagnostic accuracy" | 7,145,035 |
| #1 and #2 and #3 and #4 | | | 3,039 |
