# Supplementary material for: Digital Biomarkers for Parkinson Disease: Bibliometric Analysis and a Scoping Review of Deep Learning for Freezing of Gait
Source: J Med Internet Res. 2025 May 20;27:e71560. doi: 10.2196/71560 (PMC12134701; doi:10.2196/71560)
Supplement: Multimedia Appendix 2 [file jmir_v27i1e71560_app2.docx]

**Appendix 2. Search query for the scoping review.**

| Web of science | TS=("Parkinson's disease" OR "Parkinson disease" OR "PD" OR "Parkinson*") AND TS=("freezing of gait" OR "FOG" OR "gait freezing" OR "freez*") AND TS=("deep learning" OR "machine learning" OR "neural network*" OR "artificial neural network*" OR "deep neural network*" OR "convolutional neural network*" OR "recurrent neural network*" OR "transformer*" OR "attention*" OR "self-attention*") | 734 |
| --- | --- | --- |
| Pubmed | ("Parkinson Disease"[Mesh] OR "Parkinson disease" OR "PD" OR "Parkinson*") AND ("freezing of gait" OR "FOG" OR "gait freezing" OR "freez*") AND ("Deep Learning"[Mesh] OR "machine learning" OR "neural network*" OR "artificial neural network*" OR "deep neural network*" OR "convolutional neural network*" OR "recurrent neural network*" OR "transformer*" OR "attention*" OR "self-attention*") | 300 |
| IEEE Explore | ((((All Metadata:"deep learning") OR (All Metadata:"machine learning") OR (All Metadata:"neural network*") OR (All Metadata:"artificial neural network*") OR (All Metadata:"deep neural network*" ) OR (All Metadata:"convolutional neural network*") OR (All Metadata:"recurrent neural network*"))) AND ((All Metadata:"freezing of gait" ) OR (All Metadata:"FOG") OR (All Metadata:"gait freezing") OR (All Metadata:"freez*"))) AND ((All Metadata:"Parkinson's disease" ) OR (All Metadata:"Parkinson disease") OR (All Metadata:PD) OR (All Metadata:"Parkinson*")  OR (All Metadata:"transformer*") OR (All Metadata:"attention*") OR (All Metadata: "self-attention*")) | 148 |
| Google Scholar | ("Parkinson Disease" OR "Parkinson disease" ) AND ("freezing of gait" OR "gait freezing" ) AND ("Deep Learning" OR "neural network*" OR "artificial neural network*" OR "deep neural network*" OR "convolutional neural network*" OR "recurrent neural network*" OR "transformer*" OR "attention*" OR "self-attention*") | 100 |
| Embase | #1  'deep learning'/exp OR 'deep learning' OR 'machine learning':ti,ab,kw OR 'artificial neural network':ti,ab,kw OR 'deep neural network':ti,ab,kw OR 'convolutional neural network':ti,ab,kw OR 'recurrent neural network':ti,ab,kw OR 'neural network':ti,ab,kw  #2  'freezing of gait'/exp OR 'freezing of gait' OR ' gait freezing':ti,ab,kw  #3  'parkinson disease'/exp OR 'parkinson disease'  #1 and #2 and #3 | 204 |
