# Supplementary material for: Digital Biomarkers for Parkinson Disease: Bibliometric Analysis and a Scoping Review of Deep Learning for Freezing of Gait
Source: J Med Internet Res. 2025 May 20;27:e71560. doi: 10.2196/71560 (PMC12134701; doi:10.2196/71560)
Supplement: Multimedia Appendix 4 [file jmir_v27i1e71560_app4.doc]

**Appendix 4. Keyword merging.**

| **No** | **Keywords** | **Merged Keywords** |
| --- | --- | --- |
| 1 | parkinson's disease | parkinson's disease (pd) |
|  |  | parkinson disease |
|  |  | parkinson's |
|  |  | pd |
|  |  | Parkinson 's disease |
|  |  | parkinson-s disease |
|  |  | parkinson ?s disease |
|  |  | parkinson |
|  |  | Parkinson&apos; s disease |
|  |  | parkinsons disease (pd) |
|  |  | parkinsons's disease |
|  |  | parkinsons |
|  |  | patients with parkinson's disease |
|  |  | person with parkinson (pwp) |
| 2 | machine learning | machine learning (ml) |
|  |  | machine learning algorithms |
|  |  | machine-learning |
|  |  | interpretable machine learning model |
|  |  | interpretable machine learning |
| 3 | freezing of gait | freezing of gait (fog) |
|  |  | freezing gait (fog) |
|  |  | freeze of gait |
|  |  | fog |
| 4 | accelerometer | accelerometers |
|  |  | acceleration sensor |
|  |  | accelerometer sensor |
|  |  | triaxial accelerometer |
|  |  | tri-axial accelerometers |
| 5 | wearable device | wearable devices |
|  |  | wearable electronic device |
|  |  | wearable electronic devices |
|  |  | wearables |
|  |  | smart wearables |
| 6 | deep learning | deep-learning |
|  |  | deep learning(dl) |
| 7 | inertial measurement unit | inertial measurement units |
|  |  | inertial measurement unit (imu) |
|  |  | inertial measurement units (imu) sensor |
|  |  | inertial measurement units (imu) |
|  |  | inertial measurement unit sensors |
| 8 | time up and go | (tug) |
|  |  | time up and go test |
|  |  | timed up-and-go |
|  |  | timed up-and-go task |
|  |  | timed up-and-go test |
|  |  | timed-up-and-go test |
| 9 | convolutional neural network | convolutional neural network (cnn) |
|  |  | convolutional neural networks |
|  |  | Deep convolutional neural networks (DCNN) |
|  |  | cnn |
|  |  | cnns |
| 10 | acoustic analysis | acoustic analyses |
|  |  | acoustics analysis |
| 11 | activities of daily living | activities of daily life |
|  |  | adl |
| 12 | algorithm | algorithms |
| 13 | alzheimer's disease | alzheimer disease |
| 14 | ambulatory system | ambulatory systems |
| 15 | anticipatory postural adjustments | anticipatory postural adjustments (apas) |
| 16 | artificial neural network | artificial neural networks |
| 17 | atypical parkinsonian disorders | atypical parkinsonism |
|  |  | atypical parkinsonisms |
| 18 | automatic assessment | automated assessment |
| 19 | biomarker | biomarkers |
| 20 | body-fixed sensor | body-fixed sensors |
| 21 | capacitive sensor | capacitive sensors |
| 22 | cerebrospinal fluid marker | cerebrospinal fluid markers |
| 23 | classification | classifica-tion |
| 24 | classification algorithm | classification algorithms |
| 25 | computer-aided diagnosis | computer-aided diagnosis (cad) |
| 26 | continuous wavelet transform | continuous wavelet transforms |
| 27 | decision support system | decision support systems |
| 28 | decision making | decision-making |
| 29 | deep neural network | deep neural networks |
|  |  | deep neural networks (dnns) |
| 30 | digital biomarker | digital biomarkers |
| 31 | discrete wavelet transform | discrete wavelet transforms |
| 32 | disease | diseases |
| 33 | dual task | dual tasking |
|  |  | dual-task |
| 34 | dynamic time warping | dynamic time warping dtw |
| 35 | dyskinesia | dyskinesias |
| 36 | early parkinson's disease | early -stage parkinson's disease |
| 37 | ehealth | e-health |
| 38 | electrocardiography | electrocardiography (ecg) |
| 39 | electroencephalography | electroencephalogram (eeg) |
|  |  | eeg |
| 40 | electromyography | electromyography (emg) |
|  |  | emg |
| 41 | electrooculography | electrooculography (eog) |
| 42 | ensemble learning | ensemble classifier |
| 43 | executive function | executive functions |
| 44 | explainable artificial intelligence (xai) | explainable machine learning |
|  |  | explainable artificial intelligence (xai) |
| 45 | extreme learning machine | extreme learning machine (elm) |
| 46 | eye movement | eye movements |
|  |  | eye-movements |
| 47 | eye tracking | eye-tracking |
| 48 | fall | falls |
| 49 | feature extraction | features extraction |
| 50 | feature selection | features selection |
|  |  | feature-selection |
| 51 | finger tapping | finger tapping (ft) movement |
|  |  | finger tapping movements |
|  |  | finger taps |
|  |  | fingertapping |
|  |  | finger-tapping |
| 52 | finger tapping test | finger-tapping test |
|  |  | finger-tapping tests |
| 53 | foot pressure | foot pressure |
| 54 | freezing index | freeze index (fi) |
| 55 | Frequency domain analysis | frequency-domain analysis |
| 56 | gait analysis | gait analyses |
| 57 | gait disorder | gait disorders |
|  |  | gait motor disorder |
| 58 | gait measurement | gait measures |
| 59 | gait phases | gait phase |
| 60 | genetic algorithm | genetic algorithms |
| 61 | graph convolution neural network | graph convolutional network |
| 62 | ground reaction forces | ground reaction forces (grf) |
| 63 | gyroscope | gyroscopes |
| 64 | ground reaction force | ground reaction forces |
| 65 | graph convolutional neural network | graph convolutional network |
| 66 | hand tremor | hand tremors |
|  |  | hand-tremor |
| 67 | hidden markov model | hidden markov models |
|  |  | hidden markov models (hmms) |
|  |  | hmm |
| 68 | hoehn yahr | hoehn and yahr |
| 69 | home monitoring | home based monitoring |
|  |  | home health monitoring |
|  |  | home-monitoring |
| 70 | human pose estimation | human pose-estimation |
| 71 | imu sensor | imu sensors |
| 72 | inertial measurement unit | inertial measurement unit (imu) |
|  |  | imu |
| 73 | inertial signal | inertial signals |
| 74 | insole | insoles |
| 75 | internet of things | internet of things (iot) |
|  |  | iot |
| 76 | inertial sensors | inertial sensor |
|  |  | inertial sensor array |
|  |  | inertia sensor |
| 77 | k-nearest neighbors | k-nearest neighbor (knn) |
| 78 | levodopa-induced dyskinesia | levodopa-induced dyskinesia (lid) |
| 79 | limit of stability | limits of stability |
| 80 | long short term memory | long short term memory deep learning model |
|  |  | long short-term memory |
|  |  | long short-term memory (lstm) |
|  |  | lstm |
| 81 | magnetic sensor | magnetic sensors |
| 82 | mathematical model | mathematical models |
| 83 | mds-updrs | Mds updrs |
| 84 | metaheuristics | meta-heuristics |
| 85 | mhealth | M-health |
|  |  | mobile-health |
|  |  | mobile health |
| 86 | microsoft kinect | microsoft kinect v2 |
| 87 | mobile application | mobile app |
|  |  | mobile applications |
|  |  | mobile apps |
| 88 | mel frequency cepstral coefficients | Mfcc |
| 89 | mobile eye tracking | mobile eye-tracking |
| 90 | motion capture | motion capture (mocap) |
| 91 | motor disorder | motor disorders |
|  |  | motion disorders |
| 92 | motor fluctuations | motor fluctuation |
| 93 | movement disorders | movement disorder |
| 94 | multimodal sensor | multimodal sensors |
| 95 | near falls | Near-falls |
| 96 | neural network | neural networks |
| 97 | neurodegenerative disorders | neurodegenerative diseases |
| 98 | neurological diseases | neurological disorder |
|  |  | neurological disorders |
| 99 | neuromuscular diseases | neuromuscular disorders |
| 100 | non-motor symptoms | non-motor pd symptoms |
| 101 | objective measurement | objective measures |
| 102 | phenotype | phenotypes |
| 103 | physiologic tremor | physiological tremor |
| 104 | plantar pressure | Plantar-pressure |
| 105 | postural instability gait difficulty | postural instability and gait difficulty score (pigd) |
|  |  | postural instability gait difficulty (pigd) |
| 106 | pressure sensor | pressure sensors |
| 107 | principal component analysis | principal component analysis (pca) |
| 108 | progressive supranuclear palsy | progressive supranuclear pasly |
| 109 | quantitative motor assessment | quantitative movement assessment |
| 110 | random forest | random forest classifier |
| 111 | recurrent neural network | recurrent neural networks |
| 112 | rem sleep behavior disorder | rem sleep behaviour disorder |
| 113 | rest-activity rhythm | rest-activity rhythms |
| 114 | saccades | saccade |
| 115 | smart gloves | smart glove |
| 116 | smartphone | smartphones |
| 117 | smartwatch | smartwatches |
| 118 | speech disorder | speech disorders |
| 119 | spatiotemporal gait parameter | spatiotemporal gait parameters |
|  |  | spatiotemporal gait metrics |
|  |  | spatial-temporal gait parameters |
| 120 | speech disorder | speech disorders |
| 121 | step count | step counting |
| 122 | stepping in place | stepping-in-place |
| 123 | stride time variability | stride time (variability) |
| 124 | support vector machine | support vector machine (svm) |
|  |  | support vector machine classifier |
|  |  | support vector machines |
|  |  | support vector machines (svms) |
| 125 | surface electromyography | surface electromyography (emg) |
|  |  | surface emg |
| 126 | transformer | transformers |
| 127 | tremor assessment | tremor estimation |
|  |  | tremor evaluation |
| 128 | tremor severity | tremor severity level |
| 129 | turn | turns |
| 130 | unified parkinson's disease rating scale | unified parkinson's disease rating scale (updrs) |
|  |  | updrs |
|  |  | updrs scores |
|  |  | updrs tasks |
| 131 | upper limb | upper limbs |
| 132 | vertical ground reaction force | vertical ground reaction force (vgrf) |
|  |  | vertical ground reaction forces (vgrfs) |
| 133 | video | videos |
| 134 | walking bout | walking bouts |
| 135 | wavelet transform | wavelet transformation |
| 136 | wearable electronic device | wearable electronic devices |
| 137 | wearable inertial sensor | wearable inertial sensors |
| 138 | wearable technology | wearable technologies |
| 139 | wireless sensor networks | wireless sensor network (wsn) |
|  |  | wireless sensor network |
| 140 | walking with turning | walking with turning (wwt) |
| 141 | simple walking | simple walking (SW) |
| 142 | virtual reality | vr |
|  |  | virtual reality (vr) |
| 143 | spatiotemporal features | spatial-temporal characteristics |
| 144 | rgb-depth sensor | rgb-d sensors |
| 145 | quantitative assessment | quantitative assessments |
| 146 | prodromal | prodrome |
| 147 | parkinson's diagnosis | parkinson's disease (pd) diagnosis |
| 148 | freezing of gait assessment | fog assessment |
| 149 | freezing of gait detection | freezing of gait (fog) detection |
|  |  | fog detection |
| 150 | freezing of gait prediction | fog prediction |
