# Supplementary material for: Digital Biomarkers for Parkinson Disease: Bibliometric Analysis and a Scoping Review of Deep Learning for Freezing of Gait
Source: J Med Internet Res. 2025 May 20;27:e71560. doi: 10.2196/71560 (PMC12134701; doi:10.2196/71560)
Supplement: Multimedia Appendix 5 [file jmir_v27i1e71560_app5.docx]

**Appendix 5.The Selection Strategies and Usage Methods of Each Tool.**

**Strategy for using tools**

Current tools have certain limitations, making it difficult to analyze multiple dimensions of content using a single tool. Additionally, considering potential biases in bibliometric analysis, such as selection bias or inherent preferences in the tool's settings, we adopted a standardization approach in data processing and cross-validated results from multiple tools to ensure the accuracy and reliability of the analysis.

Specifically, mainstream bibliometric tools like Citespace, VOSviewer, and Bibliometrix lack high-resolution analysis across various components. Citespace utilizes burst detection to present content analysis in a time series, VOSviewer is more suited for social network analysis, and Bibliometrix offers a broad and deep analytical scope. However, it sometimes produces inaccurate data, especially during country output analysis. Therefore, we base our bibliometric analysis on these three tools and cross-validate the robustness of the results.

For more advanced analysis, additional tools are necessary. For identifying turning points in output over time, we rely on breakpoint regression analysis using Joinpoint. For fund analysis, Cortext provides techniques for joint analysis across various fields, making it the tool of choice for fund heatmap analysis. Gephi is used to visualize the strength of collaborations between authors from different academic backgrounds by importing matrices. For clustering analysis of high-frequency keywords, gCLUTO is a reliable tool that constructs a matrix of high-frequency keywords using Bicomb, resulting in more concentrated clusters. Echart, a Java-based charting tool, helps enrich the presentation of graphical data. The specific tool usage strategy is shown in Table 1.

**Table S1. Bibliometric tool integration strategy.**

| **Tools**  **Functions** | **Citespace** | **VOSviewer** | **Bibliometrix** | **Gephi** | **Cortext** | **Joinpoint** | **gCLUTO** | **R-studio** | **Echarts** |
| --- | --- | --- | --- | --- | --- | --- | --- | --- | --- |
| **Output** | **√** |  |  |  |  | **√** |  |  |  |
| **Author** |  | **√** |  | **√** |  |  |  |  | **√** |
| **Organization** |  | **√** |  |  |  |  |  | **√** | **√** |
| **Country** |  | **√** |  |  |  |  |  |  | **√** |
| **Keyword Theme** |  |  | **√** |  |  |  |  |  |  |
| **Keyword Burst** | **√** |  |  |  |  |  |  |  |  |
| **Keyword Progress** |  |  | **√** |  |  |  |  |  |  |
| **Keyword Cluster** |  |  |  |  |  |  | **√** |  |  |
| **Grant** |  |  |  |  | **√** |  |  |  |  |
| **Disciplinary** |  |  |  |  | **√** |  |  |  |  |
| **Highly Cited Research** |  |  | **√** |  |  |  |  |  |  |
| **Journal** |  | **√** |  |  |  |  |  |  |  |
