# Supplementary material for: Digital Biomarkers for Parkinson Disease: Bibliometric Analysis and a Scoping Review of Deep Learning for Freezing of Gait
Source: J Med Internet Res. 2025 May 20;27:e71560. doi: 10.2196/71560 (PMC12134701; doi:10.2196/71560)
Supplement: Multimedia Appendix 6 [file jmir_v27i1e71560_app6.docx]

**Appendix 6. High-output Countries, Authors, Institutions, and Funding Sources Table.**

**Table S1. The Top 10 Most Frequently Published Authors (Source from WoSCC)**

| **Author (Gender)** | **Output** | **Citations** | **Average Citations** | **H-index** | **Institution** | **Country** |
| --- | --- | --- | --- | --- | --- | --- |
| hausdorff, jeffrey m. (m) | 21 | 1142 | 54.4 | 106 | Tel Aviv University | Israel |
| maetzler, walter (m) | 19 | 566 | 29.8 | 58 | Kiel University | Germany |
| rochester, lynn (f) | 18 | 1024 | 56.9 | 71 | Newcastle University | United Kingdom |
| del din, silvia (f) | 14 | 508 | 36.3 | 32 | Newcastle University | United Kingdom |
| eskofier, bjoern m. (m) | 13 | 449 | 34.5 | 35 | Friedrich-Alexander University Erlangen-Nürnberg | Germany |
| gassner, heiko (m) | 13 | 448 | 34.5 | 22 | Friedrich-Alexander University Erlangen-Nürnberg | Germany |
| klucken, jochen (m) | 13 | 440 | 33.8 | 58 | University of Luxembourg | Luxembourg |
| giladi, nir (m) | 11 | 686 | 62.4 | 100 | Tel Aviv University | Israel |
| olmo, gabriella (f) | 11 | 156 | 14.2 | 22 | Polytechnic University of Turin | Italy |
| berg, daniela (f) | 10 | 495 | 49.5 | 89 | Kiel University | Germany |
| borzi, luigi (m) | 10 | 369 | 36.9 | 10 | Polytechnic University of Turin | Italy |
| hansen, clint (m) | 10 | 357 | 35.7 | 21 | Kiel University | Germany |
| herman, talia (f) | 10 | 156 | 15.6 | 39 | Tel Aviv University | Israel |
| lewis, simon j. g. (m) | 10 | 448 | 44.8 | 73 | Macquarie University | Australia |

**Figure S1. Top25 Author with the Strongest Citation Bursts**


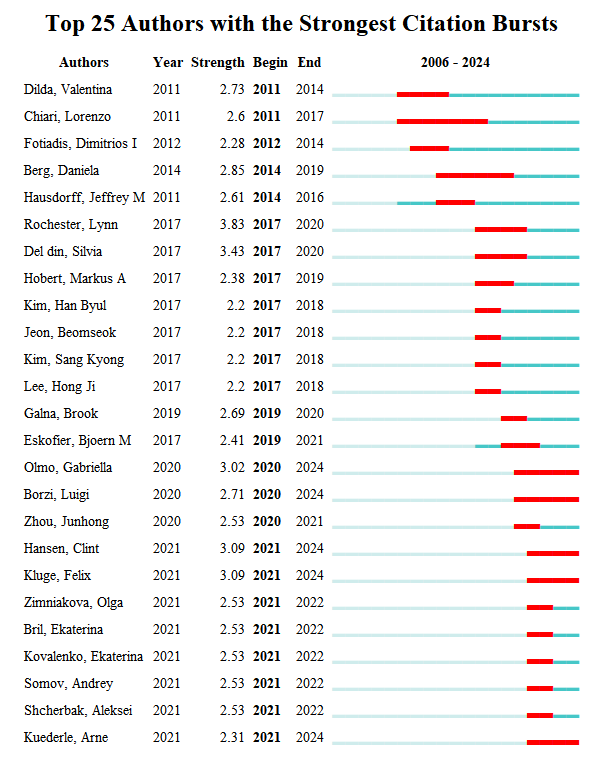

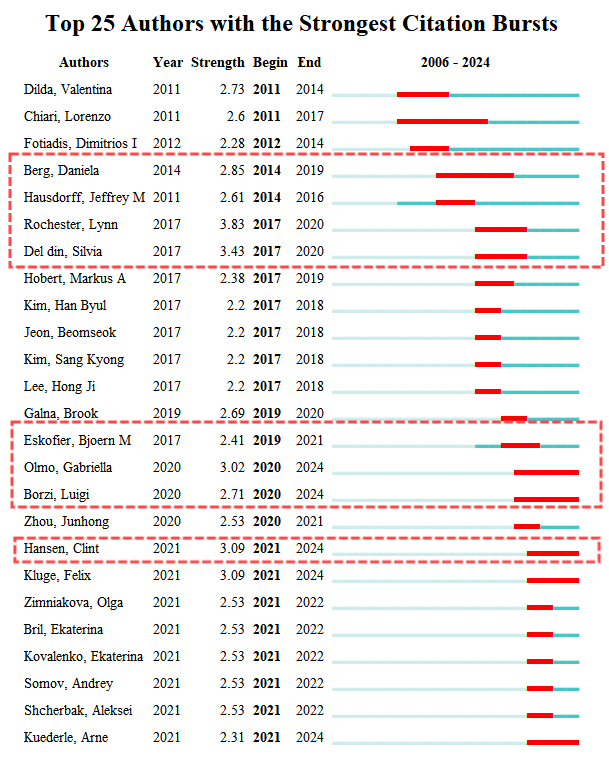


**Table S2. The Top 10 Institutions with The Highest Publication Output**

| **Organization** | **Output** | **Citations** | **PPC^a^** | **Country** | **Partner Organization** | **Total link strength** |
| --- | --- | --- | --- | --- | --- | --- |
| Tel Aviv University | 27 | 1381 | 51.15 | Israel | 46 | 140 |
| Newcastle University | 26 | 1125 | 43.27 | United Kingdom | 54 | 153 |
| Tel Aviv Sourasky Medical Center | 21 | 1113 | 53.00 | Israel | 42 | 129 |
| Politecnico di Torino | 19 | 388 | 20.42 | Italy | 43 | 105 |
| University of Sydney | 16 | 956 | 59.75 | Australia | 9 | 29 |
| Rush University | 15 | 420 | 28.00 | United States | 42 | 116 |
| Radboud University Nijmegen | 14 | 561 | 40.07 | Netherlands | 41 | 97 |
| University of Bologna | 13 | 580 | 44.62 | Italy | 33 | 75 |
| Friedrich-Alexander University Erlangen-Nürnberg | 12 | 304 | 25.33 | Germany | 8 | 26 |
| Harvard Medical School | 12 | 150 | 12.50 | United States | 31 | 69 |
| Katholieke Universiteit Leuven | 12 | 300 | 25.00 | Belgium | 25 | 61 |
| Massachusetts Institute of Technology | 12 | 683 | 56.92 | United States | 35 | 68 |
| Oregon Health & Science University | 12 | 962 | 80.17 | United States | 14 | 39 |
| Sapienza University of Rome | 12 | 375 | 31.25 | Italy | 9 | 29 |
| Capital Medical University | 11 | 91 | 8.27 | China | 16 | 39 |
| Chinese Academy of Sciences | 11 | 245 | 22.27 | China | 7 | 23 |
| University of Kiel | 11 | 283 | 25.73 | Germany | 34 | 52 |
| Universidad Politécnica de Madrid | 11 | 389 | 35.36 | Spain | 10 | 32 |
| University of Tübingen | 11 | 332 | 30.18 | Germany | 18 | 45 |
| Harvard University | 10 | 1089 | 108.90 | United States | 29 | 56 |
| Stanford University | 10 | 247 | 24.70 | United States | 12 | 34 |
| University of Rochester | 10 | 452 | 45.20 | Israel | 30 | 64 |

^a^PPC: per-paper citations.

**Table S3. The Top 10 Countries with The Highest Publication Output**

| **Country** | **Output** | **Citations** | **PPC** | **Partner Country** | **Total link strength** |
| --- | --- | --- | --- | --- | --- |
| united states | 192 | 6707 | 34.93 | 36 | 193 |
| china | 118 | 1657 | 14.54 | 22 | 59 |
| italy | 113 | 3230 | 28.58 | 32 | 125 |
| germany | 85 | 2118 | 24.92 | 30 | 110 |
| united kingdom | 78 | 2781 | 35.65 | 29 | 128 |
| canada | 47 | 1434 | 30.51 | 17 | 40 |
| spain | 46 | 1814 | 39.43 | 18 | 75 |
| israel | 35 | 1757 | 50.20 | 15 | 74 |
| australia | 32 | 1398 | 43.69 | 19 | 46 |
| japan | 32 | 476 | 14.88 | 11 | 20 |
| the netherlands | 32 | 1209 | 37.78 | 20 | 72 |

**Table S4. The Top 10 Funded Grant Programs**

| **Funders** | **Studies** | **Country** |
| --- | --- | --- |
| National Institutes of Health | 161 | United States |
| European Union | 72 | European Union |
| National Natural Science Foundation of China | 63 | China |
| Michael J. Fox Foundation | 49 | United States |
| European Commission | 48 | European Union |
| German Research Foundation | 43 | Germany |
| Parkinson's UK | 41 | United Kingdom |
| JSPS KAKENHI | 40 | Japan |
| Natural Sciences and Engineering Research Council of Canada | 38 | Canada |
| Italian Ministry of Health | 36 | Italy |
