# Supplementary material for: Digital Biomarkers for Parkinson Disease: Bibliometric Analysis and a Scoping Review of Deep Learning for Freezing of Gait
Source: J Med Internet Res. 2025 May 20;27:e71560. doi: 10.2196/71560 (PMC12134701; doi:10.2196/71560)
Supplement: Multimedia Appendix 7 [file jmir_v27i1e71560_app7.docx]

**Appendix 7. Highly Cited Journals, Studies, and High-output Journals Information Table.**

**Table S1. Basic Information of the Top 10 Most Cited Journals**

| **Co-citation journal** | **Co-citation** | **IF** | **JCR** |
| --- | --- | --- | --- |
| Movement Disorders | 1358 | 7.4 | Q1 |
| Sensors | 400 | 3.4 | Q2 |
| Parkinsonism & Related Disorders | 391 | 3.1 | Q2 |
| Neurology | 360 | 8.4 | Q1 |
| Gait & Posture | 354 | 2.2 | Q3 |
| Journal of Neurology, Neurosurgery, and Psychiatry | 315 | 8.8 | Q1 |
| Plos one | 256 | 2.9 | Q1 |
| IEEE Engineering in Medicine and Biology Magazine | 238 | 2.727 | Q1 |
| IEEE Transactions on Biomedical Engineering | 237 | 4.4 | Q2 |
| Brain | 201 | 11.9 | Q1 |

**Table S2. Basic Information of the Top 10 High-Output Journals**

| **Journal** | **output** | **IF** | **JCR** |
| --- | --- | --- | --- |
| Sensors | 100 | 3.4 | Q2 |
| Frontiers in neurology | 32 | 2.7 | Q2 |
| Ieee transactions on neural systems and rehabilitation engineering | 28 | 4.8 | Q2 |
| Journal of neuroengineering and rehabilitation | 22 | 5.2 | Q1 |
| Plos one | 19 | 2.9 | Q1 |
| Ieee transactions on biomedical engineering | 18 | 4.4 | Q2 |
| Parkinsonism & related disorders | 18 | 3.1 | Q2 |
| Scientific reports | 17 | 3.8 | Q1 |
| Biomedical signal processing and control | 16 | 4.9 | Q1 |
| IEEE access | 16 | 3.4 | Q2 |

**Table S3. Top 10 Most Cited Global Studies on Digital Biomarkers for Parkinson's Disease**

| **Paper** | **DOI** | **Title** | **TC** | **^a^TC per Year** | **Normalized TC** | **Key Findings** |
| --- | --- | --- | --- | --- | --- | --- |
| GALNA B, 2014, GAIT POSTURE[1] | [10.1016/j.gaitpost.2014.01.008](https://doi.org/10.1016/j.gaitpost.2014.01.008) | [Accuracy of the Microsoft Kinect sensor for measuring movement in people with Parkinson's disease](https://www.sciencedirect.com/science/article/pii/S0966636214000241) | 382 | 31.83 | 5.68 | The Microsoft Kinect sensor can accurately measure temporal features of movement and the spatial features of larger motions in Parkinson's disease patients. However, its spatial accuracy is lower for smaller movements, such as hand clenching or toe tapping. Despite this limitation, Kinect serves as a low-cost and convenient tool for remote monitoring of motor symptoms in Parkinson's disease patients. |
| PATEL S, 2009, IEEE T INF TECHNOL B[2] | [10.1109/TITB.2009.2033471](https://doi.org/10.1109/TITB.2009.2033471) | Monitoring motor fluctuations in patients with Parkinson's disease using wearable sensors | 375 | 22.06 | 4.44 | Data collected through wearable sensors, combined with the support vector machine algorithm, can reliably estimate the severity of tremor, bradykinesia, and dyskinesia in Parkinson's disease patients. |
| MOORE ST, 2008, J NEUROSCI METH[3] | [10.1016/j.jneumeth.2007.08.023](https://doi.org/10.1016/j.jneumeth.2007.08.023) | Ambulatory monitoring of freezing of gait in Parkinson's disease | 358 | 19.89 | 3.97 | Real-time detection of freezing of gait in Parkinson's disease patients can be achieved by monitoring the vertical acceleration of the legs. Frequency analysis of the acceleration data reveals that during FOG episodes, the legs exhibit distinct high-frequency vibrations (in the 3-8 Hz range), which differ from the leg movements observed during normal standing. |
| SALARIAN A, 2010, IEEE T NEUR SYS REH[4] | [10.1109/TNSRE.2010.2047606](https://doi.org/10.1109/TNSRE.2010.2047606) | [iTUG, a sensitive and reliable measure of mobility](https://ieeexplore.ieee.org/abstract/document/5446357/) | 348 | 21.75 | 4.51 | The iTUG (instrumented Timed Up and Go) test, utilizing portable inertial sensors, offers a more precise assessment of motor function in early Parkinson's disease patients, surpassing the traditional time-based measurement approach of the TUG test. |
| SALARIAN A, 2007, IEEE T BIO-MED ENG[5] | [10.1109/TBME.2006.886670](https://doi.org/10.1109/TBME.2006.886670) | Quantification of tremor and bradykinesia in Parkinson's disease using a novel ambulatory monitoring system | 280 | 14.74 | 2.34 | A portable monitoring system based on a miniature gyroscope has been developed to quantitatively assess tremor and bradykinesia in Parkinson's disease patients in real-time. The system demonstrated high sensitivity (99.5%) and specificity (94.2%) during daily activities, and significant correlations were found between tremor amplitude, bradykinesia parameters, and the Unified Parkinson's Disease Rating Scale. |
| ZHAN AD, 2018, JAMA NEUROL[6] | [10.1001/jamaneurol.2018.0809](https://doi.org/10.1001/jamaneurol.2018.0809) | Using smartphones and machine learning to quantify Parkinson disease severity: the mobile Parkinson disease score | 235 | 29.38 | 5.09 | The mobile Parkinson's Disease Score, developed using smartphones and machine learning technology, can objectively quantify the severity of motor symptoms in Parkinson's disease patients. It allows for frequent and objective assessments in real-life environments. |
| NYEIN HYY, 2021, NAT COMMUN[7] | [10.1038/s41467-021-22109-z](https://doi.org/10.1038/s41467-021-22109-z) | A wearable patch for continuous analysis of thermoregulatory sweat at rest | 215 | 43 | 12.38 | This study developed a wearable microfluidic sweat monitoring device that can analyze the levodopa concentration in sweat in real-time during rest, providing a non-invasive monitoring method for medication management in Parkinson's disease. |
| MARIANI B, 2013, IEEE T BIO-MED ENG[8] | [10.1109/TBME.2012.2227317](https://doi.org/10.1109/TBME.2012.2227317) | On-shoe wearable sensors for gait and turning assessment of patients with Parkinson's disease | 196 | 15.08 | 2.95 | A wearable sensor-based evaluation method, using shoe-mounted sensors and specialized algorithms, has been proposed. By extracting 3D gait parameters, it successfully and objectively quantifies the motor performance of Parkinson's disease patients during the "Timed Up and Go" test and long-distance walking. |
| WEISS A, 2014, PLOS ONE[9] | [10.1371/journal.pone.0096675](https://doi.org/10.1371/journal.pone.0096675) | Objective assessment of fall risk in Parkinson's disease using a body-fixed sensor worn for 3 days | 169 | 14.08 | 2.51 | Wearing a body sensor for three consecutive days effectively assesses the fall risk in Parkinson's disease patients. Notably, in patients who have not experienced falls in the past year, the sensor data successfully predicted the timing of their first fall. |
| TROJANIELLO D, 2014, J NEUROENG REHABIL[10] | [10.1186/1743-0003-11-152](https://doi.org/10.1186/1743-0003-11-152) | Estimation of step-by-step spatio-temporal parameters of normal and impaired gait using shank-mounted magneto-inertial sensors: application to elderly, hemiparetic, parkinsonian and choreic gait | 167 | 13.92 | 2.48 | This study proposes a gait spatiotemporal parameter estimation method based on bilateral ankle magnetic inertial measurement units . The method was validated in Parkinson's disease patients and demonstrated accurate detection of gait events and estimation of gait parameters. |

^a^TC: Total Citations

**References**

1. Galna B, Barry G, Jackson D, Mhiripiri D, Olivier P, Rochester L. Accuracy of the Microsoft Kinect sensor for measuring movement in people with Parkinson's disease. Gait & posture. 2014 Apr;39(4):1062-8. PMID: 24560691. doi: 10.1016/j.gaitpost.2014.01.008.

2. Patel S, Lorincz K, Hughes R, Huggins N, Growdon J, Standaert D, et al. Monitoring motor fluctuations in patients with Parkinson's disease using wearable sensors. IEEE transactions on information technology in biomedicine : a publication of the IEEE Engineering in Medicine and Biology Society. 2009 Nov;13(6):864-73. PMID: 19846382. doi: 10.1109/titb.2009.2033471.

3. Moore ST, MacDougall HG, Ondo WG. Ambulatory monitoring of freezing of gait in Parkinson's disease. Journal of neuroscience methods. 2008 Jan 30;167(2):340-8. PMID: 17928063. doi: 10.1016/j.jneumeth.2007.08.023.

4. Salarian A, Horak FB, Zampieri C, Carlson-Kuhta P, Nutt JG, Aminian K. iTUG, a sensitive and reliable measure of mobility. IEEE transactions on neural systems and rehabilitation engineering : a publication of the IEEE Engineering in Medicine and Biology Society. 2010 Jun;18(3):303-10. PMID: 20388604. doi: 10.1109/tnsre.2010.2047606.

5. Salarian A, Russmann H, Wider C, Burkhard PR, Vingerhoets FJ, Aminian K. Quantification of tremor and bradykinesia in Parkinson's disease using a novel ambulatory monitoring system. IEEE transactions on bio-medical engineering. 2007 Feb;54(2):313-22. PMID: 17278588. doi: 10.1109/tbme.2006.886670.

6. Zhan A, Mohan S, Tarolli C, Schneider RB, Adams JL, Sharma S, et al. Using Smartphones and Machine Learning to Quantify Parkinson Disease Severity: The Mobile Parkinson Disease Score. JAMA neurology. 2018 Jul 1;75(7):876-80. PMID: 29582075. doi: 10.1001/jamaneurol.2018.0809.

7. Nyein HYY, Bariya M, Tran B, Ahn CH, Brown BJ, Ji W, et al. A wearable patch for continuous analysis of thermoregulatory sweat at rest. Nature communications. 2021 Mar 23;12(1):1823. PMID: 33758197. doi: 10.1038/s41467-021-22109-z.

8. Mariani B, Jiménez MC, Vingerhoets FJ, Aminian K. On-shoe wearable sensors for gait and turning assessment of patients with Parkinson's disease. IEEE transactions on bio-medical engineering. 2013 Jan;60(1):155-8. PMID: 23268531. doi: 10.1109/tbme.2012.2227317.

9. Weiss A, Herman T, Giladi N, Hausdorff JM. Objective assessment of fall risk in Parkinson's disease using a body-fixed sensor worn for 3 days. PloS one. 2014;9(5):e96675. PMID: 24801889. doi: 10.1371/journal.pone.0096675.

10. Trojaniello D, Cereatti A, Pelosin E, Avanzino L, Mirelman A, Hausdorff JM, et al. Estimation of step-by-step spatio-temporal parameters of normal and impaired gait using shank-mounted magneto-inertial sensors: application to elderly, hemiparetic, parkinsonian and choreic gait. Journal of neuroengineering and rehabilitation. 2014 Nov 11;11:152. PMID: 25388296. doi: 10.1186/1743-0003-11-152.
