# Supplementary material for: Digital Biomarkers for Parkinson Disease: Bibliometric Analysis and a Scoping Review of Deep Learning for Freezing of Gait
Source: J Med Internet Res. 2025 May 20;27:e71560. doi: 10.2196/71560 (PMC12134701; doi:10.2196/71560)
Supplement: Multimedia Appendix 8 [file jmir_v27i1e71560_app8.docx]

**Appendix 8. Distribution of High-frequency Keywords.**

The formula for this calculation is based on *Nmax*, where Nmax represents the frequency of the highest-frequency keyword, and *N* is the threshold for meeting the high-frequency keyword standard.

$$N=0.749\times\sqrt{N_{\max}}$$

**High-Frequency Keyword Frequency Distribution Table**

| **Rank** | **Keyword** | **Occurrences** |
| --- | --- | --- |
| 1 | parkinson's disease | 493 |
| 2 | machine learning | 110 |
| 3 | freezing of gait | 87 |
| 4 | accelerometer | 83 |
| 5 | wearable sensors | 76 |
| 6 | gait analysis | 68 |
| 7 | gait | 58 |
| 8 | wearable device | 45 |
| 9 | inertial sensors | 44 |
| 10 | deep learning | 39 |
| 11 | inertial measurement unit | 39 |
| 12 | tremor | 38 |
| 13 | feature extraction | 36 |
| 14 | classification | 30 |
| 15 | convolutional neural network | 29 |
| 16 | diseases | 25 |
| 17 | essential tremor | 25 |
| 18 | movement disorders | 25 |
| 19 | bradykinesia | 23 |
| 20 | sensors | 22 |
| 21 | support vector machine | 22 |
| 22 | gyroscope | 21 |
| 23 | telemedicine | 19 |
| 24 | digital health | 18 |
| 25 | smartphone | 18 |
| 26 | task analysis | 17 |
