# Supplementary material for: Digital Biomarkers for Parkinson Disease: Bibliometric Analysis and a Scoping Review of Deep Learning for Freezing of Gait
Source: J Med Internet Res. 2025 May 20;27:e71560. doi: 10.2196/71560 (PMC12134701; doi:10.2196/71560)
Supplement: Multimedia Appendix 9 [file jmir_v27i1e71560_app9.docx]

**Appendix 9. Basic Information on Parkinson's Freezing of Gait Deep Learning Model Research.**

| **Author, Year** | **Country** | **Detection/Prediction** | **Sample Size** | **Number of Events** | **Device Used** | **Device Placement Location** |
| --- | --- | --- | --- | --- | --- | --- |
| Po-Kai Yang (2024)[1] | Belgium | Detection | 12PD | 133 FOG Events in 346 IMU Trials | Shimmer3 IMU | Pelvis, bilateral shins, and talus |
| Po-Kai Yang (2024)[2] | Belgium | Detection | 18PD | 545 trials, totaling 275.18 minutes of recordings. Normal gait accounted for 75.49%, Trembling for 12.96%, Motor impairment for 3.85%, freezing episodes for 4.02%, and sitting for 3.67%. | Shimmer3 IMU | Pelvis, bilateral shins, and talus |
| Boyan Wang(2024)[3] | China | Prediction | 10PD (Daphnet FOG Dataset) | 237 FOG Events | Wearable Sensor (IMU) | Ankle, Thigh, and Trunk |
| Hua Sun(2024)[4] | China | Prediction | 10PD (Daphnet FOG Dataset) | 237 FOG Events | Wearable Sensor (IMU) | Ankle, Thigh, and Trunk |
| Luis Sigcha(2024)[5] | Ireland | Detection | 38 PD (Rempark, Daphnet, and Oday Datasets) | 1558 FOG events | Wearable Sensor (IMU) | **Rempark**:Worn on the waist;  **Daphnet**:Worn on the shins, thighs, and waist; **Oday**:Worn on the feet, shins, waist, and chest |
| Mohamed Shaban(2024)[6] | United States | Detection | 37 PD (Kaggle Dataset) | / | Wearable three-axis acceleromete | Lower back |
| [Jae-Min Park](https://pubmed.ncbi.nlm.nih.gov/?term="Park JM"[Author])(2024)[7] | Korea | Detection | 4PD | / | Pedar insole pressure sensor system (Wearable device) | Embedded in insoles and covering the entire foot, including the toes, forefoot, and heel regions |
| [Hwayoung Park](https://pubmed.ncbi.nlm.nih.gov/?term="Park H"[Author])(2024)[8] | Korea | Detection | 30 Freezers vs. 30 Non-Freezers vs. 30 HC | / | 3D motion capture system (Vicon MX-T1) (Non-wearable device） | 39 reflective markers placed on the head, upper limbs, lower limbs, and trunk |
| [Yuki Kondo](https://ieeexplore.ieee.org/author/683667730844449)(2024)[9] | Japan | Detection | 9 PD, 7 Progressive Supranuclear Palsy | / | Panasonic HC-V720M-T monocular RGB camera | / |
| Debin Huang(2024)[10] | China | Prediction | 12PD | 334 FOG Events | TDK MPU6050 six-degree-of-freedom inertial sensor | Lateral sides of the left and right calves, and the fifth lumbar vertebra (L5) on the waist |
| Zeeshan Habib(2024)[11] | Pakistan | Detection | 15PD | / | WiFi transmitter | The transmitter (Tx) and receiver (Rx) were placed within a 10-meter line-of-sight range |
| [Lloyd L.Y. Chan](https://ieeexplore.ieee.org/author/830140785463385)(2024)[12] | Australia | Detection | 107PD | 50,962 data windows representing 37, 809 seconds from 97 participants with at least one FOG event | Waist-worn device (Dynaport Hybrid, McRoberts) | Waist |
| [Emilie Charlotte Klaver](https://pubmed.ncbi.nlm.nih.gov/?term="Klaver EC"[Author])(2023)[13] | Netherlands | Detection | 80PD | 1435 FOG Events | MVN Awinda motion capture system (Wearable device) | Waist, upper leg, lower leg, and both feet |
| [Kun Hu](https://ieeexplore.ieee.org/author/37087103935)  (2023)[14] | Australia | Detection | 20PD | 340 Experiments, 3,793 Seconds of FOG Segments, Representing 22.5% of the Total Time | Zeno Gait Mat pressure insole, video camera (Non-wearable device） | **Video camera**: Front-facing view, focusing on the patient's lower limbs  **Pressure insole**: Pressure applied while the patient walks |
| [Kun Hu](https://ieeexplore.ieee.org/author/37087103935)  (2023)[15] | Australia | Detection | 21PD | 393 Clinical Assessments, FOG Event Duration:3,816 Seconds (22.8% of Total Data) | Zeno Gait Mat pressure insole (Non-wearable device） | Fixed on the pressure mat |
| Luigi Borzì(2023)[16] | Italy | Detection+Prediction | 118 PD, 21 HC (ADL Dataset, 6MWT Dataset, REMPARK Dataset) | Over 17 hours of valid data, recording 1,110 FOG events | Wearable IMU sensor | Left waist or lower back |
| Luigi Borzì(2023)[17] | Italy | Detection | 81PD(ADL Dataset, REMPARK Dataset, Daphnet Dataset) | 14 hours of data, including 121.9 minutes with FOG events | Wearable IMU sensor and smartphone | Lower leg, upper leg, lower back, and left waist |
| [Rishabh Bajpai](https://ieeexplore.ieee.org/author/37088665187)(2023)[18] | India | Prediction | 8PD | / | Electroencephalogram system, Wearable IMU sensor | Head, left lower leg, left forearm |
| Luis Sigcha(2022)[19] | Spain | Detection | 21PD (REMPARK-FOG Dataset) | 1058 FOG Events | Single three-axis accelerometer | Left side of the waist |
| [Bohan Shi](https://ieeexplore.ieee.org/author/37088479017)(2022)[20] | Singapore | Detection | 63PD | 486 FOG Events | Wearable IMU sensor | Ankle joint, 7th cervical vertebra |
| [Johanna O’Day](https://pubmed.ncbi.nlm.nih.gov/?term="O%E2%80%99Day J"[Author])(2022)[21] | United States | Detection | 7PD | 211 FOG Events | IMU (APDM Inc.) | **Setup**: Chest, lumbar spine, tops of both feet, outer sides of both ankles  **Extended Positions**: Head, wrists, outer thighs |
| [Nader Naghavi](https://ieeexplore.ieee.org/author/37086834486)(2022)[22] | United States | Detection+Prediction | 7PD | 154 FOG Events | Opal Sensor (APDM Inc.) | Both ankles |
| [Benjamin Filtjens](https://pubmed.ncbi.nlm.nih.gov/?term="Filtjens B"[Author])(2022)[23] | Belgium | Detection | 14HC, 31 FOG+, 17 FOG- (MoCap dataset) | 190 FOG Events | Vicon 3D motion capture system (Non-wearable device） | 9 optical markers placed on key anatomical locations of the lower limbs and pelvis |
| [Gaurav Shalin](https://pubmed.ncbi.nlm.nih.gov/?term="Shalin G"[Author])(2021)[24] | Canada | Detection+Prediction | 11PD | 362 FOG Events | FScan Pressure Sensor Insole (Wearable device) | Inside the insole |
| [Antonio Prado](https://ieeexplore.ieee.org/author/37086063498)(2021)[25] | United States | Detection | 10PD | 108 gait trials with FoG events | DeepSole System (Wearable device) | Inside the insole |
| [Benjamin Filtjens](https://pubmed.ncbi.nlm.nih.gov/?term="Filtjens B"[Author])(2021)[26] | Belgium | Detection | 14 HC, 14 FOG-, 14 FOG+ (Collected by KU Leuven Research Team) | 56 FOG events | Vicon 3D Motion Analysis System (Non-wearable device） | 34 reflective markers placed on the hip, knee, and ankle joints |
| [Ali Haddadi Esfahani](https://ieeexplore.ieee.org/author/37088989939)(2021)[27] | Germany | Detection | 10PD (Daphnet FOG Dataset) | **FOG data proportion**:9.7%  **Normal gait data proportion**: 90.3% | Wearable Sensor (IMU) | Ankle, Thigh, and Trunk |
| [Thomas Bikias](https://pubmed.ncbi.nlm.nih.gov/?term="Bikias T"[Author])(2021)[28] | Greece | Detection | 11 PD (CuPiD IMU dataset) | 184 FOG events | Commercial smart watch | Single wrist |
| [Luis Sigcha](https://pubmed.ncbi.nlm.nih.gov/?term="Sigcha L"[Author])(2020)[29] | Spain | Detection | 21PD (Rodríguez-Martín et al. dataset) | FOG events account for 10.5% of the dataset | Wearable sensor (IMU) | Waist |
| [Syed Aziz Shah](https://ieeexplore.ieee.org/author/37086149871)(2020)[30] | United Kingdom | Detection | 120 participants | / | Wi-Fi and radar imaging system | / |
| Bochen Li(2020)[31] | China | Detection | 10PD (Daphnet FOG Dataset) | 237 FOG Events | Wearable Sensor (IMU) | Ankle, Thigh, and Trunk |
| [Kun Hu](https://ieeexplore.ieee.org/author/37087103935)(2020)[32] | Australia | Detection | 45PD | 167 segments, with a total duration of 25.5 hours, and 8.7% of the segments containing FoG events | Camera | / |
| Amira S. Ashour(2020)[33] | Egypt | Detection | 10PD | 8 patients with FoG events (no further details provided) | Tri-axial accelerometer | Knee, hip, and ankle |
| Ahsen Tahir(2019)[34] | United Kingdom | Detection | 15PD | 1350 FOG Events | Wi-Fi and radar imaging system | / |
| Rubén San-Segundo(2019)[35] | Spain | Detection | 10PD (Daphnet FOG Dataset) | 237 FOG Events | Wearable Sensor (IMU) | Ankle, Thigh, and Trunk |
| Yi Xia(2018)[36] | China | Detection | 10PD (Daphnet FOG Dataset) | 237 FOG Events | Wearable Sensor (IMU) | Ankle, Thigh, and Trunk |
| Han Byul Kim(2018)[37] | Korea | Detection | 32PD | 5839 data windows (each 2.5 seconds), 41.7% of the data related to freezing of gait (approximately 2436 FOG segments) | Google Nexus 5 smartphone | Pocket |
| Julià Camps(2018)[38] | Spain | Detection | 21PD (MASPARK Project) | **Training Set**: 2,230,800 samples (16.27% FOG)  **Validation Set**: 818,400 samples (18.04% FOG)  **Test Set**: 844,800 samples (13.04% FOG) | Wearable IMU | Left side of the waist |
| Mohd Sadiq(2022)[39] | India | Detection | 10PD (Daphnet FOG Dataset) | 35,531data windows (each 4 seconds) | Wearable Sensor (IMU) | Ankle, Thigh, and Trunk |
| [Abdullah H Al-Nefaie](https://pubmed.ncbi.nlm.nih.gov/?size=20&term=Al-Nefaie+AH&cauthor_id=38966531)(2024)[40] | Saudi Arabia | Prediction | 20 FOG-, 45 FOG+ (Kaggle Dataset) | / | Tri-axial accelerometer | Lower back |

1. Yang PK, Filtjens B, Ginis P, Goris M, Nieuwboer A, Gilat M, et al. Freezing of gait assessment with inertial measurement units and deep learning: effect of tasks, medication states, and stops. Journal of neuroengineering and rehabilitation. 2024 Feb 13;21(1):24. PMID: 38350964. doi: 10.1186/s12984-024-01320-1.

2. Yang PK, Filtjens B, Ginis P, Goris M, Nieuwboer A, Gilat M, et al. Automatic Detection and Assessment of Freezing of Gait Manifestations. IEEE transactions on neural systems and rehabilitation engineering : a publication of the IEEE Engineering in Medicine and Biology Society. 2024;32:2699-708. PMID: 39028610. doi: 10.1109/tnsre.2024.3431208.

3. Wang B, Hu X, Ge R, Xu C, Zhang J, Gao Z, et al. Prediction of Freezing of Gait in Parkinson's disease based on multi-channel time-series neural network. Artificial intelligence in medicine. 2024 Aug;154:102932. PMID: 39004005. doi: 10.1016/j.artmed.2024.102932.

4. Sun H, Ye Q, Xia Y. Predicting freezing of gait in patients with Parkinson's disease by combination of Manually-Selected and deep learning features. Biomedical Signal Processing and Control. 2024 Feb;88. PMID: WOS:001092892400001. doi: 10.1016/j.bspc.2023.105639.

5. Sigcha L, Borzi L, Olmo G. Deep learning algorithms for detecting freezing of gait in Parkinson's disease: A cross-dataset study. Expert Systems with Applications. 2024 Dec 1;255. PMID: WOS:001261265000001. doi: 10.1016/j.eswa.2024.124522.

6. Shaban M. A novel variational mode decomposition based convolutional neural network for the identification of freezing of gait intervals for patients with Parkinson's disease. Machine Learning with Applications. 2024 Jun;16. PMID: WOS:001237314800001. doi: 10.1016/j.mlwa.2024.100553.

7. Park JM, Moon CW, Lee BC, Oh E, Lee J, Jang WJ, et al. Detection of freezing of gait in Parkinson's disease from foot-pressure sensing insoles using a temporal convolutional neural network. Frontiers in aging neuroscience. 2024;16:1437707. PMID: 39092074. doi: 10.3389/fnagi.2024.1437707.

8. Park H, Shin S, Youm C, Cheon SM. Deep learning-based detection of affected body parts in Parkinson's disease and freezing of gait using time-series imaging. Scientific reports. 2024 Oct 10;14(1):23732. PMID: 39390087. doi: 10.1038/s41598-024-75445-7.

9. Kondo Y, Bando K, Suzuki I, Miyazaki Y, Nishida D, Hara T, et al. Video-Based Detection of Freezing of Gait in Daily Clinical Practice in Patients With Parkinsonism. IEEE transactions on neural systems and rehabilitation engineering : a publication of the IEEE Engineering in Medicine and Biology Society. 2024;32:2250-60. PMID: 38865235. doi: 10.1109/tnsre.2024.3413055.

10. Huang D, Wu C, Wang Y, Zhang Z, Chen C, Li L, et al. Episode-level prediction of freezing of gait based on wearable inertial signals using a deep neural network model. Biomedical Signal Processing and Control. 2024 Feb;88. PMID: WOS:001102804300001. doi: 10.1016/j.bspc.2023.105613.

11. Habib Z, Mughal MA, Khan MA, Shabaz M. WiFOG: Integrating deep learning and hybrid feature selection for accurate freezing of gait detection. Alexandria Engineering Journal. 2024 Jan;86:481-93. PMID: WOS:001134999900001. doi: 10.1016/j.aej.2023.11.075.

12. Chan LLY, Yang S, Aswani M, Kark L, Henderson E, Lord SR, et al. Development, Validation, and Limits of Freezing of Gait Detection Using a Single Waist-Worn Device. IEEE transactions on bio-medical engineering. 2024 Oct;71(10):3024-31. PMID: 38814761. doi: 10.1109/tbme.2024.3407059.

13. Klaver EC, Heijink IB, Silvestri G, van Vugt JPP, Janssen S, Nonnekes J, et al. Comparison of state-of-the-art deep learning architectures for detection of freezing of gait in Parkinson's disease. Frontiers in neurology. 2023;14:1306129. PMID: 38178885. doi: 10.3389/fneur.2023.1306129.

14. Hu K, Wang Z, Martens KAE, Hagenbuchner M, Bennamoun M, Tsoi AC, et al. Graph Fusion Network-Based Multimodal Learning for Freezing of Gait Detection. IEEE transactions on neural networks and learning systems. 2023 Mar;34(3):1588-600. PMID: 34464270. doi: 10.1109/tnnls.2021.3105602.

15. Hu K, Mei S, Wang W, Martens KAE, Wang L, Lewis SJG, et al. Multi-Level Adversarial Spatio-Temporal Learning for Footstep Pressure Based FoG Detection. IEEE journal of biomedical and health informatics. 2023 Aug;27(8):4166-77. PMID: 37227913. doi: 10.1109/jbhi.2023.3272902.

16. Borzì L, Sigcha L, Rodríguez-Martín D, Olmo G. Real-time detection of freezing of gait in Parkinson's disease using multi-head convolutional neural networks and a single inertial sensor. Artificial intelligence in medicine. 2023 Jan;135:102459. PMID: 36628783. doi: 10.1016/j.artmed.2022.102459.

17. Borzì L, Sigcha L, Olmo G. Context Recognition Algorithms for Energy-Efficient Freezing-of-Gait Detection in Parkinson's Disease. Sensors (Basel, Switzerland). 2023 Apr 30;23(9). PMID: 37177629. doi: 10.3390/s23094426.

18. Bajpai R, Khare S, Joshi D. A Multimodal Model-Fusion Approach for Improved Prediction of Freezing of Gait in Parkinson's Disease. Ieee Sensors Journal. 2023 Jul 15;23(14):16168-75. PMID: WOS:001030784400092. doi: 10.1109/jsen.2023.3284656.

19. Sigcha L, Borzi L, Pavon I, Costa N, Costa S, Arezes P, et al. Improvement of Performance in Freezing of Gait detection in Parkinson's Disease using Transformer networks and a single waist-worn triaxial accelerometer. Engineering Applications of Artificial Intelligence. 2022 Nov;116. PMID: WOS:000869747400005. doi: 10.1016/j.engappai.2022.105482.

20. Shi B, Tay A, Au WL, Tan DML, Chia NSY, Yen SC. Detection of Freezing of Gait Using Convolutional Neural Networks and Data From Lower Limb Motion Sensors. IEEE transactions on bio-medical engineering. 2022 Jul;69(7):2256-67. PMID: 34986092. doi: 10.1109/tbme.2022.3140258.

21. O'Day J, Lee M, Seagers K, Hoffman S, Jih-Schiff A, Kidziński Ł, et al. Assessing inertial measurement unit locations for freezing of gait detection and patient preference. Journal of neuroengineering and rehabilitation. 2022 Feb 13;19(1):20. PMID: 35152881. doi: 10.1186/s12984-022-00992-x.

22. Naghavi N, Wade E. Towards Real-Time Prediction of Freezing of Gait in Patients With Parkinson's Disease: A Novel Deep One-Class Classifier. IEEE journal of biomedical and health informatics. 2022 Apr;26(4):1726-36. PMID: 34375292. doi: 10.1109/jbhi.2021.3103071.

23. Filtjens B, Ginis P, Nieuwboer A, Slaets P, Vanrumste B. Automated freezing of gait assessment with marker-based motion capture and multi-stage spatial-temporal graph convolutional neural networks. Journal of neuroengineering and rehabilitation. 2022 May 21;19(1):48. PMID: 35597950. doi: 10.1186/s12984-022-01025-3.

24. Shalin G, Pardoel S, Lemaire ED, Nantel J, Kofman J. Prediction and detection of freezing of gait in Parkinson's disease from plantar pressure data using long short-term memory neural-networks. Journal of neuroengineering and rehabilitation. 2021 Nov 27;18(1):167. PMID: 34838066. doi: 10.1186/s12984-021-00958-5.

25. Prado A, Kwei SK, Vanegas-Arroyave N, Agrawal SK. Continuous Identification of Freezing of Gait in Parkinson's Patients Using Artificial Neural Networks and Instrumented Shoes. Ieee Transactions on Medical Robotics and Bionics. 2021 Aug;3(3):554-62. PMID: WOS:000896668000003. doi: 10.1109/tmrb.2021.3091526.

26. Filtjens B, Ginis P, Nieuwboer A, Afzal MR, Spildooren J, Vanrumste B, et al. Modelling and identification of characteristic kinematic features preceding freezing of gait with convolutional neural networks and layer-wise relevance propagation. BMC medical informatics and decision making. 2021 Dec 7;21(1):341. PMID: 34876110. doi: 10.1186/s12911-021-01699-0.

27. Esfahani AH, Dyka Z, Ortmann S, Langendoerfer P. Impact of Data Preparation in Freezing of Gait Detection Using Feature-Less Recurrent Neural Network. Ieee Access. 2021 2021;9:138120-31. PMID: WOS:000706816400001. doi: 10.1109/access.2021.3117543.

28. Bikias T, Iakovakis D, Hadjidimitriou S, Charisis V, Hadjileontiadis LJ. DeepFoG: An IMU-Based Detection of Freezing of Gait Episodes in Parkinson's Disease Patients via Deep Learning. Frontiers in robotics and AI. 2021;8:537384. PMID: 34113654. doi: 10.3389/frobt.2021.537384.

29. Sigcha L, Costa N, Pavón I, Costa S, Arezes P, López JM, et al. Deep Learning Approaches for Detecting Freezing of Gait in Parkinson's Disease Patients through On-Body Acceleration Sensors. Sensors (Basel, Switzerland). 2020 Mar 29;20(7). PMID: 32235373. doi: 10.3390/s20071895.

30. Shah SA, Tahir A, Ahmad J, Zahid A, Pervaiz H, Shah SY, et al. Sensor Fusion for Identification of Freezing of Gait Episodes Using Wi-Fi and Radar Imaging. Ieee Sensors Journal. 2020 Dec 1;20(23):14410-22. PMID: WOS:000589257300062. doi: 10.1109/jsen.2020.3004767.

31. Li B, Yao Z, Wang J, Wang S, Yang X, Sun Y. Improved Deep Learning Technique to Detect Freezing of Gait in Parkinson's Disease Based on Wearable Sensors. Electronics. 2020 Nov;9(11). PMID: WOS:000593599400001. doi: 10.3390/electronics9111919.

32. Hu K, Wang Z, Wang W, Martens KAE, Wang L, Tan T, et al. Graph Sequence Recurrent Neural Network for Vision-based Freezing of Gait Detection. IEEE transactions on image processing : a publication of the IEEE Signal Processing Society. 2019 Oct 15. PMID: 31634131. doi: 10.1109/tip.2019.2946469.

33. Ashour AS, El-Attar A, Dey N, Abd El-Kader H, Abd El-Naby MM. Long short term memory based patient-dependent model for FOG detection in Parkinson's disease. Pattern Recognition Letters. 2020 Mar;131:23-9. PMID: WOS:000521971700004. doi: 10.1016/j.patrec.2019.11.036.

34. Tahir A, Ahmad J, Shah SA, Morison G, Skelton DA, Larijani H, et al. WiFreeze: Multiresolution Scalograms for Freezing of Gait Detection in Parkinson's Leveraging 5G Spectrum with Deep Learning. Electronics. 2019 Dec;8(12). PMID: WOS:000506678200060. doi: 10.3390/electronics8121433.

35. San-Segundo R, Navarro-Hellin H, Torres-Sanchez R, Hodgins J, De la Torre F. Increasing Robustness in the Detection of Freezing of Gait in Parkinson's Disease. Electronics. 2019 Feb;8(2). PMID: WOS:000460746500004. doi: 10.3390/electronics8020119.

36. Xia Y, Zhang J, Ye Q, Cheng N, Lu Y, Zhang D. Evaluation of deep convolutional neural networks for detection of freezing of gait in Parkinson's disease patients. Biomedical Signal Processing and Control. 2018 Sep;46:221-30. PMID: WOS:000447109800024. doi: 10.1016/j.bspc.2018.07.015.

37. Kim HB, Lee HJ, Lee WW, Kim SK, Jeon HS, Park HY, et al. Validation of Freezing-of-Gait Monitoring Using Smartphone. Telemedicine and E-Health. 2018 Nov;24(11):899-907. PMID: WOS:000431103100001. doi: 10.1089/tmj.2017.0215.

38. Camps J, Sama A, Martin M, Rodriguez-Martin D, Perez-Lopez C, Moreno Arostegui JM, et al. Deep learning for freezing of gait detection in Parkinson's disease patients in their homes using a waist-worn inertial measurement unit. Knowledge-Based Systems. 2018 Jan 1;139:119-31. PMID: WOS:000417773400011. doi: 10.1016/j.knosys.2017.10.017.

39. Sadiq M, Khan MT, Masood SJC, Materials, Continua. Attention-Based Deep Learning Model for Early Detection of Parkinson’s Disease. 2022;71(3).

40. Al-Nefaie AH, Aldhyani THH, Farhah N, Koundal D. Intelligent diagnosis system based on artificial intelligence models for predicting freezing of gait in Parkinson's disease. Frontiers in medicine. 2024;11:1418684. PMID: 38966531. doi: 10.3389/fmed.2024.1418684.
