# Supplementary material for: Digital Biomarkers for Parkinson Disease: Bibliometric Analysis and a Scoping Review of Deep Learning for Freezing of Gait
Source: J Med Internet Res. 2025 May 20;27:e71560. doi: 10.2196/71560 (PMC12134701; doi:10.2196/71560)
Supplement: Multimedia Appendix 10 [file jmir_v27i1e71560_app10.docx]

**Appendix 10. Data Processing and Key Findings of Parkinson's Freezing of Gait Deep Learning Models.**

| **Author, Year** | **Gait Task** | **Gait Data Segmentation Method (Time)** | **Comparison with Doctor (ICC)** | **Key Findings** |
| --- | --- | --- | --- | --- |
| Po-Kai Yang (2024)[1] | TUG (Timed Up and Go), 360-Degree Turn Task | Sliding Window (1s, 2s, 4s) | %TF(ICC = 0.92), #FOG(ICC = 0.95) | The model exhibited high agreement with expert assessments during freezing of gait (FOG)-inducing tasks and under medicated states, even when movement was halted. |
| Po-Kai Yang (2024)[2] | TUG, 360-Degree Turn Task | Sliding Window (4s) | %TF(ICC =0.92), %TF-T(ICC =0.86), %TF-A(ICC =0.78) | The deep learning (DL) model effectively detected FOG in Parkinson’s disease (PD) patients, including episodes of Trembling and Akinesia. |
| Boyan Wang(2024)[3] | Straight Corridor Walk, Random Walk, and Daily Activity Walking | Fixed window approach (0.5s) | / | An efficient gait data processing framework was proposed by integrating multi-channel sensor data with techniques such as causal dilated convolutions. This framework effectively captures long-term dependencies and demonstrates strong robustness across various gait behaviors |
| Hua Sun(2024)[4] | Straight Corridor Walk, Random Walk, and Daily Activity Walking | Sliding Window (1s, 3s, 5s) | / | By combining expert-manually extracted features with deep learning-based automatically extracted features, the accuracy and reliability of FOG prediction were significantly improved. The model provides an average prediction duration of approximately 2.02 seconds, offering a better approach for gait management in Parkinson's disease patients. |
| Luis Sigcha(2024)[5] | A series of daily life activities performed in a home environment  , Straight corridor walking  , Random walking  , Daily activity walking  , Simulated daily activities in an experimental environment, including walking, turning, standing, and other common movements. | Sliding window (2s) | / | The study highlighted the limitations of deep learning in terms of cross-dataset generalization and emphasized the importance of standardized data collection and personalized models. |
| Mohamed Shaban(2024)[6] | Start hesitation  Turning  Normal walking | Sliding window (2s) | / | This study proposes a method based on Variational Mode Decomposition and CNN, achieving high temporal resolution (7.8 ms) for FOG detection in Parkinson’s disease patients, with an accuracy of 98.8%. The model demonstrated excellent performance on large-scale datasets. |
| [Jae-Min Park](https://pubmed.ncbi.nlm.nih.gov/?term="Park JM"[Author])(2024)[7] | Normal gait  Walking through a narrow passage  Gait under special conditions  360-degree turn | Fixed window (time segments of 8 frames and 6 frames) | / | The time convolutional neural network (TCNN) model based on the insole pressure sensor efficiently detects FOG in Parkinson's disease patients, demonstrating exceptional accuracy and robustness across multiple task scenarios. |
| [Hwayoung Park](https://pubmed.ncbi.nlm.nih.gov/?term="Park H"[Author])(2024)[8] | 360-degree turn task | Fixed window (9s) | / | By combining time-series images with a CNN model, the study successfully identified key body landmark points closely associated with Parkinson's disease and FOG. The study also introduced a novel, objective disease classification and assessment method. |
| [Yuki Kondo](https://ieeexplore.ieee.org/author/683667730844449)(2024)[9] | TUG | Fixed window (1s) | FOG duration(ICC = 0.94), %TF (ICC = 0.84). | A video-based, non-contact FOG detection system was developed, integrating posture estimation with deep learning to achieve high-precision FOG detection. The system demonstrated strong performance with FOG duration (ICC = 0.94) and %TF (ICC = 0.84). |
| Debin Huang(2024)[10] | Simple U-turn task and complex walking task | Sliding window (0.5s) | / | Compared to other methods, such as SEC-ALSTM and DeepFoG, FoG-Net maintains continuity in gait state predictions, avoiding frequent high-frequency prediction switches. This approach aligns with the actual patterns of gait change. FoG-Net is capable of predicting FOG events up to 7.17 seconds in advance of their occurrence. |
| Zeeshan Habib(2024)[11] | Sitting, slow walking, fast walking, active stopping | Fixed window (3s) | / | A deep learning-based, non-invasive system called **WiFOG** was proposed, which utilizes WiFi Channel State Information to detect FOG in PD patients. |
| [Lloyd L.Y. Chan](https://ieeexplore.ieee.org/author/830140785463385)(2024)[12] | Complex gait task | Adaptive window segmentation (0.3 to 3s) | Total FOG duration(ICC = 0.83) | The study demonstrated high-performance, low-power FOG detection using a single waist-worn device, providing a non-invasive and accurate monitoring method for PD patients. |
| [Emilie Charlotte Klaver](https://pubmed.ncbi.nlm.nih.gov/?term="Klaver EC"[Author])(2023)[13] | Walking straight forward  Walking through a narrow passage  Turning in place (360-degree turn)  Walking gait trajectory (including 360-degree turn)  Voluntary stopping | Sliding window (2s) | / | In detecting FOG in PD patients, traditional CNN outperformed more complex models (InceptionTime and MiniRocket). |
| [Kun Hu](https://ieeexplore.ieee.org/author/37087103935)  (2023)[14] | TUG, Walking over a virtual obstacle | Fixed window (1s) | / | This study is one of the first to apply multimodal learning with graph convolutional networks to FOG detection. It demonstrated the potential of multimodal data fusion and graph-based modeling methods to improve detection accuracy, handle missing modalities, and reduce data redundancy. |
| [Kun Hu](https://ieeexplore.ieee.org/author/37087103935)  (2023)[15] | TUG in a virtual reality environment | Fixed window (1s) | / | This study is the first to use pressure mat data for FOG detection. An end-to-end deep learning architecture based on multi-level adversarial spatiotemporal learning was proposed, enabling subject-independent FOG feature extraction. |
| Luigi Borzì(2023)[16] | Various walking tasks, ADL-like tasks, 6MWT, and UPDRS-related tests | Sliding window (2s) | / | This study proposed a real-time FOG detection algorithm based on a single inertial sensor and multi-head CNN. The detection time per instance was 43 milliseconds, and 52.3% of FOG events were detected up to 3.1 seconds in advance. |
| Luigi Borzì(2023)[17] | Free walking, outdoor walking, and ADL tasks (a series of walking tasks) | Sliding window (2s) | / | This study developed a context-aware FOG detection framework that combines lightweight gait screening and efficient FOG detection algorithms, significantly reducing computational load. |
| [Rishabh Bajpai](https://ieeexplore.ieee.org/author/37088665187)(2023)[18] | Straight walking, turning, gait initiation and termination, and obstacle avoidance | Fixed window (0.128s) | / | The study compared the performance of unimodal models (using only EEG or IMU) with a multimodal model. At a prediction horizon of 1 second, the fused model achieved an accuracy of 92.1% and an F1 score of 0.85, demonstrating the effectiveness of multimodal fusion in FOG prediction tasks. |
| Luis Sigcha(2022)[19] | A series of daily living activities in a home environment | Fixed window (3.2s) | / | This study proposed a model combining Transformer and CNN (FOG-Transformer) that processes time-series data using a self-attention mechanism. Compared to traditional methods (such as CNN-LSTM), this approach not only improved FOG detection performance but also reduced computational complexity. |
| [Bohan Shi](https://ieeexplore.ieee.org/author/37088479017)(2022)[20] | 7mTUG | Sliding window (4s) | / | The study applied Continuous Wavelet Transform (CWT) to convert lower limb IMU sensor data into time-frequency domain representations (2D CWT coefficient maps). This method effectively captures both time-domain and frequency-domain features, overcoming the challenges of FOG signal complexity and heterogeneity. |
| [Johanna O’Day](https://pubmed.ncbi.nlm.nih.gov/?term="O%E2%80%99Day J"[Author])(2022)[21] | Obstacle turning task | Sliding window (2s) | %TF:ICC = 0.93, #FOG: ICC = 0.95 | The study found that using more sensors does not necessarily improve FOG detection performance. The optimal technical performance was achieved with three IMUs placed on the waist and ankles. |
| [Nader Naghavi](https://ieeexplore.ieee.org/author/37086834486)(2022)[22] | Turning task and stopping task | Sliding window (2s) | / | The study developed a real-time system for predicting FoG in PD patients. A novel method based on a deep one-class classifier was introduced, specifically designed for detecting and predicting FoG in new patients without FoG training data. The system predicted FoG events 1 second before their occurrence in 21.9% of cases, and detected 87.4% of events within 2 seconds after they occurred. |
| [Benjamin Filtjens](https://pubmed.ncbi.nlm.nih.gov/?term="Filtjens B"[Author])(2022)[23] | Straight walking, 180-degree turn, 360-degree turn | Frame-by-frame segmentation (time segmentation of gait data using a frame-by-frame annotation method) | %TF:r = 0.93, #FOG:r = 0.75 | This study introduced a multi-stage spatiotemporal graph convolutional network (MS-GCN) for automatic evaluation of FoG. The method demonstrates high accuracy and robustness, offering an objective alternative to traditional manual annotation for detecting and evaluating FoG in PD patients. |
| [Gaurav Shalin](https://pubmed.ncbi.nlm.nih.gov/?term="Shalin G"[Author])(2021)[24] | Straight walking, turning, walking through narrow spaces, voluntary/required stopping, etc. | Event-based segmentation | / | This study demonstrates the feasibility and effectiveness of using foot pressure data combined with an Long Short-Term Memory (LSTM) neural network model for real-time detection and prediction of FoG in PD patients. |
| [Antonio Prado](https://ieeexplore.ieee.org/author/37086063498)(2021)[25] | Continuous walking on the Zeno Walkway mat | Sliding window (0.5s) | / | This study developed a real-time detection system combining artificial neural networks and instrumented shoes, effectively identifying FoG in PD patients with high sensitivity (96.0%) and accuracy (99.5%). |
| [Benjamin Filtjens](https://pubmed.ncbi.nlm.nih.gov/?term="Filtjens B"[Author])(2021)[26] | Straight walking, 180° turns, 360° turns | Event-based segmentation | / | This study identified key kinematic features preceding the onset of FoG, including reduced peak knee flexion during the swing phase, fixed ankle dorsiflexion, and fixed knee extension during the stance phase. These features contribute to the accurate prediction of FoG occurrence. |
| [Ali Haddadi Esfahani](https://ieeexplore.ieee.org/author/37088989939)(2021)[27] | Straight Corridor Walk, Random Walk, and Daily Activity Walking | Fixed window approach (1s) | / | An innovative data imputation method was proposed, combined with a feature-independent LSTM model, achieving efficient FoG detection with 92.57% sensitivity and 95.62% specificity on the Daphnet dataset. This approach significantly outperforms existing methods. |
| [Thomas Bikias](https://pubmed.ncbi.nlm.nih.gov/?term="Bikias T"[Author])(2021)[28] | Continuous walking, 180°/360° turns, obstacle avoidance, and stop actions | Sliding window (3s) | / | The DeepFoG model, based on single wrist IMU sensor data, efficiently detects FoG events in PD patients. It demonstrates excellent sensitivity and specificity, simplifies device usage, and enhances user acceptance and clinical application potential. |
| [Luis Sigcha](https://pubmed.ncbi.nlm.nih.gov/?term="Sigcha L"[Author])(2020)[29] | Daily walking, turning movements, standing, initiation, and other daily gait tasks | Sliding window (3.2s) | / | The CNN-LSTM model based on a single waist sensor successfully achieved high-performance FoG detection (AUC of 92.3%) by combining FFT data representations with temporal context windows. This approach provides an effective solution for low-cost, real-time freezing of gait detection. |
| [Syed Aziz Shah](https://ieeexplore.ieee.org/author/37086149871)(2020)[30] | Fast walking, slow walking, voluntary stopping, sit-to-stand, and freezing of gait (FoG) | Fixed window (5s or 10s) | / | By fusing Wi-Fi signals with radar imaging and leveraging an enhanced Autoencoder algorithm, the study achieved high-precision, non-contact detection of FoG in PD patients, with a classification accuracy of 98.1%. |
| Bochen Li(2020)[31] | Straight Corridor Walk, Random Walk, and Daily Activity Walking | Sliding window (4s) | / | An efficient FoG detection system based on the deep learning framework SEC-ALSTM was proposed, significantly improving both detection accuracy and operational efficiency. |
| [Kun Hu](https://ieeexplore.ieee.org/author/37087103935)(2020)[32] | TUG | Fixed window (1s) | / | The GS-RNN (Graph Sequence Recurrent Neural Network) model achieved high-precision visual detection of FoG in Parkinson's disease by modeling the dynamic joint graph sequences of the human body, with an AUC of 0.90. |
| Amira S. Ashour(2020)[33] | Not specifically detailed | Event-based segmentation | / | The patient-dependent model based on LSTM networks efficiently detected FoG in PD patients with an average accuracy of 83.38%, outperforming traditional machine learning methods. |
| Ahsen Tahir(2019)[34] | Fast walking, slow walking, sit-to-stand transitions, voluntary stopping, and other FoG-triggering tasks | Event-based segmentation | / | The WiFreeze system, utilizing Wi-Fi CSI and deep learning methods, achieved non-invasive, high-precision detection of FoG in PD patients, with an accuracy of up to 99.7%. |
| Rubén San-Segundo(2019)[35] | Straight Corridor Walk, Random Walk, and Daily Activity Walking | Sliding window (4s) | / | An efficient FoG detection system was proposed, combining deep CNN with contextual windows. This system achieved optimal performance with an equal error rate (EER) of 12.5% and an AUC of 0.93, significantly enhancing detection robustness and accuracy. |
| Yi Xia(2018)[36] | Straight Corridor Walk, Random Walk, and Daily Activity Walking | Sliding window (4s) | / | A deep CNN-based FoG detection system was developed, achieving over 99% classification accuracy in a patient-dependent setting and an average accuracy of 80.70% in a patient-independent setting. |
| Han Byul Kim(2018)[37] | Straight walking task, doorway task, turning task | Sliding window (2.5s) | / | This study proposed a smartphone-based FoG detection system using a CNN, achieving a sensitivity of 93.8% and specificity of 90.1%, enabling high-precision, non-invasive remote monitoring. |
| Julià Camps(2018)[38] | A series of daily living activities performed in a home environment | Fixed window (2.5s) | / | In an early study, a 1D CNN with a Spectral Window Stacking data representation method was used to achieve high-precision automatic detection of FoG in PD patients within a home environment. |
| Mohd Sadiq(2022)[39] | Straight Corridor Walk, Random Walk, and Daily Activity Walking | Sliding window (4s) | / | This study proposes a deep learning model based on an attention mechanism for the early detection of PD and FoG events, as well as for measuring the severity of PD. The model achieves accuracies of 98.74%, 98.72%, and 98.05% for FoG detection, PD diagnosis, and UPDRS scoring, respectively. |
| [Abdullah H Al-Nefaie](https://pubmed.ncbi.nlm.nih.gov/?size=20&term=Al-Nefaie+AH&cauthor_id=38966531)(2024)[40] | Simulate daily living tasks | Event-based segmentation | / | By applying various machine learning and deep learning algorithms, this study successfully classified FoG events in PD patients, achieving an accuracy of up to 91% in the gait classification task. |

### **Abbreviations**

TUG: Timed Up and Go

FOG: freezing of gait

DL: deep learning

PD: Parkinson’s disease

CNN: Convolutional Neural Networks

VMD: Variational Mode Decomposition

TCNN: The time convolutional neural network

GCN: graph convolutional networks

CWT: Continuous Wavelet Transform

1. Yang PK, Filtjens B, Ginis P, Goris M, Nieuwboer A, Gilat M, et al. Freezing of gait assessment with inertial measurement units and deep learning: effect of tasks, medication states, and stops. Journal of neuroengineering and rehabilitation. 2024 Feb 13;21(1):24. PMID: 38350964. doi: 10.1186/s12984-024-01320-1.

2. Yang PK, Filtjens B, Ginis P, Goris M, Nieuwboer A, Gilat M, et al. Automatic Detection and Assessment of Freezing of Gait Manifestations. IEEE transactions on neural systems and rehabilitation engineering : a publication of the IEEE Engineering in Medicine and Biology Society. 2024;32:2699-708. PMID: 39028610. doi: 10.1109/tnsre.2024.3431208.

3. Wang B, Hu X, Ge R, Xu C, Zhang J, Gao Z, et al. Prediction of Freezing of Gait in Parkinson's disease based on multi-channel time-series neural network. Artificial intelligence in medicine. 2024 Aug;154:102932. PMID: 39004005. doi: 10.1016/j.artmed.2024.102932.

4. Sun H, Ye Q, Xia Y. Predicting freezing of gait in patients with Parkinson's disease by combination of Manually-Selected and deep learning features. Biomedical Signal Processing and Control. 2024 Feb;88. PMID: WOS:001092892400001. doi: 10.1016/j.bspc.2023.105639.

5. Sigcha L, Borzi L, Olmo G. Deep learning algorithms for detecting freezing of gait in Parkinson's disease: A cross-dataset study. Expert Systems with Applications. 2024 Dec 1;255. PMID: WOS:001261265000001. doi: 10.1016/j.eswa.2024.124522.

6. Shaban M. A novel variational mode decomposition based convolutional neural network for the identification of freezing of gait intervals for patients with Parkinson's disease. Machine Learning with Applications. 2024 Jun;16. PMID: WOS:001237314800001. doi: 10.1016/j.mlwa.2024.100553.

7. Park JM, Moon CW, Lee BC, Oh E, Lee J, Jang WJ, et al. Detection of freezing of gait in Parkinson's disease from foot-pressure sensing insoles using a temporal convolutional neural network. Frontiers in aging neuroscience. 2024;16:1437707. PMID: 39092074. doi: 10.3389/fnagi.2024.1437707.

8. Park H, Shin S, Youm C, Cheon SM. Deep learning-based detection of affected body parts in Parkinson's disease and freezing of gait using time-series imaging. Scientific reports. 2024 Oct 10;14(1):23732. PMID: 39390087. doi: 10.1038/s41598-024-75445-7.

9. Kondo Y, Bando K, Suzuki I, Miyazaki Y, Nishida D, Hara T, et al. Video-Based Detection of Freezing of Gait in Daily Clinical Practice in Patients With Parkinsonism. IEEE transactions on neural systems and rehabilitation engineering : a publication of the IEEE Engineering in Medicine and Biology Society. 2024;32:2250-60. PMID: 38865235. doi: 10.1109/tnsre.2024.3413055.

10. Huang D, Wu C, Wang Y, Zhang Z, Chen C, Li L, et al. Episode-level prediction of freezing of gait based on wearable inertial signals using a deep neural network model. Biomedical Signal Processing and Control. 2024 Feb;88. PMID: WOS:001102804300001. doi: 10.1016/j.bspc.2023.105613.

11. Habib Z, Mughal MA, Khan MA, Shabaz M. WiFOG: Integrating deep learning and hybrid feature selection for accurate freezing of gait detection. Alexandria Engineering Journal. 2024 Jan;86:481-93. PMID: WOS:001134999900001. doi: 10.1016/j.aej.2023.11.075.

12. Chan LLY, Yang S, Aswani M, Kark L, Henderson E, Lord SR, et al. Development, Validation, and Limits of Freezing of Gait Detection Using a Single Waist-Worn Device. IEEE transactions on bio-medical engineering. 2024 Oct;71(10):3024-31. PMID: 38814761. doi: 10.1109/tbme.2024.3407059.

13. Klaver EC, Heijink IB, Silvestri G, van Vugt JPP, Janssen S, Nonnekes J, et al. Comparison of state-of-the-art deep learning architectures for detection of freezing of gait in Parkinson's disease. Frontiers in neurology. 2023;14:1306129. PMID: 38178885. doi: 10.3389/fneur.2023.1306129.

14. Hu K, Wang Z, Martens KAE, Hagenbuchner M, Bennamoun M, Tsoi AC, et al. Graph Fusion Network-Based Multimodal Learning for Freezing of Gait Detection. IEEE transactions on neural networks and learning systems. 2023 Mar;34(3):1588-600. PMID: 34464270. doi: 10.1109/tnnls.2021.3105602.

15. Hu K, Mei S, Wang W, Martens KAE, Wang L, Lewis SJG, et al. Multi-Level Adversarial Spatio-Temporal Learning for Footstep Pressure Based FoG Detection. IEEE journal of biomedical and health informatics. 2023 Aug;27(8):4166-77. PMID: 37227913. doi: 10.1109/jbhi.2023.3272902.

16. Borzì L, Sigcha L, Rodríguez-Martín D, Olmo G. Real-time detection of freezing of gait in Parkinson's disease using multi-head convolutional neural networks and a single inertial sensor. Artificial intelligence in medicine. 2023 Jan;135:102459. PMID: 36628783. doi: 10.1016/j.artmed.2022.102459.

17. Borzì L, Sigcha L, Olmo G. Context Recognition Algorithms for Energy-Efficient Freezing-of-Gait Detection in Parkinson's Disease. Sensors (Basel, Switzerland). 2023 Apr 30;23(9). PMID: 37177629. doi: 10.3390/s23094426.

18. Bajpai R, Khare S, Joshi D. A Multimodal Model-Fusion Approach for Improved Prediction of Freezing of Gait in Parkinson's Disease. Ieee Sensors Journal. 2023 Jul 15;23(14):16168-75. PMID: WOS:001030784400092. doi: 10.1109/jsen.2023.3284656.

19. Sigcha L, Borzi L, Pavon I, Costa N, Costa S, Arezes P, et al. Improvement of Performance in Freezing of Gait detection in Parkinson's Disease using Transformer networks and a single waist-worn triaxial accelerometer. Engineering Applications of Artificial Intelligence. 2022 Nov;116. PMID: WOS:000869747400005. doi: 10.1016/j.engappai.2022.105482.

20. Shi B, Tay A, Au WL, Tan DML, Chia NSY, Yen SC. Detection of Freezing of Gait Using Convolutional Neural Networks and Data From Lower Limb Motion Sensors. IEEE transactions on bio-medical engineering. 2022 Jul;69(7):2256-67. PMID: 34986092. doi: 10.1109/tbme.2022.3140258.

21. O'Day J, Lee M, Seagers K, Hoffman S, Jih-Schiff A, Kidziński Ł, et al. Assessing inertial measurement unit locations for freezing of gait detection and patient preference. Journal of neuroengineering and rehabilitation. 2022 Feb 13;19(1):20. PMID: 35152881. doi: 10.1186/s12984-022-00992-x.

22. Naghavi N, Wade E. Towards Real-Time Prediction of Freezing of Gait in Patients With Parkinson's Disease: A Novel Deep One-Class Classifier. IEEE journal of biomedical and health informatics. 2022 Apr;26(4):1726-36. PMID: 34375292. doi: 10.1109/jbhi.2021.3103071.

23. Filtjens B, Ginis P, Nieuwboer A, Slaets P, Vanrumste B. Automated freezing of gait assessment with marker-based motion capture and multi-stage spatial-temporal graph convolutional neural networks. Journal of neuroengineering and rehabilitation. 2022 May 21;19(1):48. PMID: 35597950. doi: 10.1186/s12984-022-01025-3.

24. Shalin G, Pardoel S, Lemaire ED, Nantel J, Kofman J. Prediction and detection of freezing of gait in Parkinson's disease from plantar pressure data using long short-term memory neural-networks. Journal of neuroengineering and rehabilitation. 2021 Nov 27;18(1):167. PMID: 34838066. doi: 10.1186/s12984-021-00958-5.

25. Prado A, Kwei SK, Vanegas-Arroyave N, Agrawal SK. Continuous Identification of Freezing of Gait in Parkinson's Patients Using Artificial Neural Networks and Instrumented Shoes. Ieee Transactions on Medical Robotics and Bionics. 2021 Aug;3(3):554-62. PMID: WOS:000896668000003. doi: 10.1109/tmrb.2021.3091526.

26. Filtjens B, Ginis P, Nieuwboer A, Afzal MR, Spildooren J, Vanrumste B, et al. Modelling and identification of characteristic kinematic features preceding freezing of gait with convolutional neural networks and layer-wise relevance propagation. BMC medical informatics and decision making. 2021 Dec 7;21(1):341. PMID: 34876110. doi: 10.1186/s12911-021-01699-0.

27. Esfahani AH, Dyka Z, Ortmann S, Langendoerfer P. Impact of Data Preparation in Freezing of Gait Detection Using Feature-Less Recurrent Neural Network. Ieee Access. 2021 2021;9:138120-31. PMID: WOS:000706816400001. doi: 10.1109/access.2021.3117543.

28. Bikias T, Iakovakis D, Hadjidimitriou S, Charisis V, Hadjileontiadis LJ. DeepFoG: An IMU-Based Detection of Freezing of Gait Episodes in Parkinson's Disease Patients via Deep Learning. Frontiers in robotics and AI. 2021;8:537384. PMID: 34113654. doi: 10.3389/frobt.2021.537384.

29. Sigcha L, Costa N, Pavón I, Costa S, Arezes P, López JM, et al. Deep Learning Approaches for Detecting Freezing of Gait in Parkinson's Disease Patients through On-Body Acceleration Sensors. Sensors (Basel, Switzerland). 2020 Mar 29;20(7). PMID: 32235373. doi: 10.3390/s20071895.

30. Shah SA, Tahir A, Ahmad J, Zahid A, Pervaiz H, Shah SY, et al. Sensor Fusion for Identification of Freezing of Gait Episodes Using Wi-Fi and Radar Imaging. Ieee Sensors Journal. 2020 Dec 1;20(23):14410-22. PMID: WOS:000589257300062. doi: 10.1109/jsen.2020.3004767.

31. Li B, Yao Z, Wang J, Wang S, Yang X, Sun Y. Improved Deep Learning Technique to Detect Freezing of Gait in Parkinson's Disease Based on Wearable Sensors. Electronics. 2020 Nov;9(11). PMID: WOS:000593599400001. doi: 10.3390/electronics9111919.

32. Hu K, Wang Z, Wang W, Martens KAE, Wang L, Tan T, et al. Graph Sequence Recurrent Neural Network for Vision-based Freezing of Gait Detection. IEEE transactions on image processing : a publication of the IEEE Signal Processing Society. 2019 Oct 15. PMID: 31634131. doi: 10.1109/tip.2019.2946469.

33. Ashour AS, El-Attar A, Dey N, Abd El-Kader H, Abd El-Naby MM. Long short term memory based patient-dependent model for FOG detection in Parkinson's disease. Pattern Recognition Letters. 2020 Mar;131:23-9. PMID: WOS:000521971700004. doi: 10.1016/j.patrec.2019.11.036.

34. Tahir A, Ahmad J, Shah SA, Morison G, Skelton DA, Larijani H, et al. WiFreeze: Multiresolution Scalograms for Freezing of Gait Detection in Parkinson's Leveraging 5G Spectrum with Deep Learning. Electronics. 2019 Dec;8(12). PMID: WOS:000506678200060. doi: 10.3390/electronics8121433.

35. San-Segundo R, Navarro-Hellin H, Torres-Sanchez R, Hodgins J, De la Torre F. Increasing Robustness in the Detection of Freezing of Gait in Parkinson's Disease. Electronics. 2019 Feb;8(2). PMID: WOS:000460746500004. doi: 10.3390/electronics8020119.

36. Xia Y, Zhang J, Ye Q, Cheng N, Lu Y, Zhang D. Evaluation of deep convolutional neural networks for detection of freezing of gait in Parkinson's disease patients. Biomedical Signal Processing and Control. 2018 Sep;46:221-30. PMID: WOS:000447109800024. doi: 10.1016/j.bspc.2018.07.015.

37. Kim HB, Lee HJ, Lee WW, Kim SK, Jeon HS, Park HY, et al. Validation of Freezing-of-Gait Monitoring Using Smartphone. Telemedicine and E-Health. 2018 Nov;24(11):899-907. PMID: WOS:000431103100001. doi: 10.1089/tmj.2017.0215.

38. Camps J, Sama A, Martin M, Rodriguez-Martin D, Perez-Lopez C, Moreno Arostegui JM, et al. Deep learning for freezing of gait detection in Parkinson's disease patients in their homes using a waist-worn inertial measurement unit. Knowledge-Based Systems. 2018 Jan 1;139:119-31. PMID: WOS:000417773400011. doi: 10.1016/j.knosys.2017.10.017.

39. Sadiq M, Khan MT, Masood SJC, Materials, Continua. Attention-Based Deep Learning Model for Early Detection of Parkinson’s Disease. 2022;71(3).

40. Al-Nefaie AH, Aldhyani THH, Farhah N, Koundal D. Intelligent diagnosis system based on artificial intelligence models for predicting freezing of gait in Parkinson's disease. Frontiers in medicine. 2024;11:1418684. PMID: 38966531. doi: 10.3389/fmed.2024.1418684.
