# Supplementary material for: Digital Biomarkers for Parkinson Disease: Bibliometric Analysis and a Scoping Review of Deep Learning for Freezing of Gait
Source: J Med Internet Res. 2025 May 20;27:e71560. doi: 10.2196/71560 (PMC12134701; doi:10.2196/71560)
Supplement: Multimedia Appendix 11 [file jmir_v27i1e71560_app11.docx]

**Appendix 11. Data Types and Multimodal Data Fusion.**

**Table S1. Data Types for Deep Learning Models in Parkinson's Gait Freezing.**

| **Studies** | **Modal data** |
| --- | --- |
| Po-Kai Yang (2024)[1] | Inertial sensor data |
| Po-Kai Yang (2024)[2] | Inertial sensor data |
| Boyan Wang(2024)[3] | Inertial sensor data |
| Hua Sun(2024)[4] | Inertial sensor data |
| Luis Sigcha(2024)[5] | Inertial sensor data |
| Mohamed Shaban(2024)[6] | Inertial sensor data |
| [Jae-Min Park](https://pubmed.ncbi.nlm.nih.gov/?term="Park JM"[Author])(2024)[7] | Pressure sensor data |
| [Hwayoung Park](https://pubmed.ncbi.nlm.nih.gov/?term="Park H"[Author])(2024)[8] | Optical motion capture sensor data |
| [Yuki Kondo](https://ieeexplore.ieee.org/author/683667730844449)(2024)[9] | Video data |
| Debin Huang(2024)[10] | Inertial sensor data |
| Zeeshan Habib(2024)[11] | Wireless signal data |
| [Lloyd L.Y. Chan](https://ieeexplore.ieee.org/author/830140785463385)(2024)[12] | Inertial sensor data |
| [Emilie Charlotte Klaver](https://pubmed.ncbi.nlm.nih.gov/?term="Klaver EC"[Author])(2023)[13] | Inertial sensor data |
| [Kun Hu](https://ieeexplore.ieee.org/author/37087103935)  (2023)[14] | Video data + Pressure sensor data |
| [Kun Hu](https://ieeexplore.ieee.org/author/37087103935)  (2023)[15] | Pressure sensor data |
| Luigi Borzì(2023)[16] | Inertial sensor data |
| Luigi Borzì(2023)[17] | Inertial sensor data |
| [Rishabh Bajpai](https://ieeexplore.ieee.org/author/37088665187)(2023)[18] | Neural sensor data + Inertial sensor data |
| Luis Sigcha(2022)[19] | Inertial sensor data |
| [Bohan Shi](https://ieeexplore.ieee.org/author/37088479017)(2022)[20] | Inertial sensor data |
| [Johanna O’Day](https://pubmed.ncbi.nlm.nih.gov/?term="O%E2%80%99Day J"[Author])(2022)[21] | Inertial sensor data |
| [Nader Naghavi](https://ieeexplore.ieee.org/author/37086834486)(2022)[22] | Inertial sensor data |
| [Benjamin Filtjens](https://pubmed.ncbi.nlm.nih.gov/?term="Filtjens B"[Author])(2022)[23] | Optical motion capture sensor data |
| [Gaurav Shalin](https://pubmed.ncbi.nlm.nih.gov/?term="Shalin G"[Author])(2021)[24] | Pressure sensor data |
| [Antonio Prado](https://ieeexplore.ieee.org/author/37086063498)(2021)[25] | Pressure sensor data |
| [Benjamin Filtjens](https://pubmed.ncbi.nlm.nih.gov/?term="Filtjens B"[Author])(2021)[26] | Optical motion capture sensor data |
| [Ali Haddadi Esfahani](https://ieeexplore.ieee.org/author/37088989939)(2021)[27] | Inertial sensor data |
| [Thomas Bikias](https://pubmed.ncbi.nlm.nih.gov/?term="Bikias T"[Author])(2021)[28] | Inertial sensor data |
| [Luis Sigcha](https://pubmed.ncbi.nlm.nih.gov/?term="Sigcha L"[Author])(2020)[29] | Inertial sensor data |
| [Syed Aziz Shah](https://ieeexplore.ieee.org/author/37086149871)(2020)[30] | Radar sensor data + Wireless signal data |
| Bochen Li(2020)[31] | Inertial sensor data |
| [Kun Hu](https://ieeexplore.ieee.org/author/37087103935)(2020)[32] | Video data |
| Amira S. Ashour(2020)[33] | Inertial sensor data |
| Ahsen Tahir(2019)[34] | Wireless signal data |
| Rubén San-Segundo(2019)[35] | Inertial sensor data |
| Yi Xia(2018)[36] | Inertial sensor data |
| Han Byul Kim(2018)[37] | Inertial sensor data |
| Julià Camps(2018)[38] | Inertial sensor data |
| Mohd Sadiq(2022)[39] | Inertial sensor data |
| [Abdullah H Al-Nefaie](https://pubmed.ncbi.nlm.nih.gov/?size=20&term=Al-Nefaie+AH&cauthor_id=38966531)(2024)[40] | Inertial sensor data |

**Table S2. Performance Range of Various Deep Learning Models for Parkinson's Gait Freezing.**

| **Modal data** | **Performance** | | | | **References** |
| --- | --- | --- | --- | --- | --- |
| **Unimodal research** | **ACC** | **SEN** | **SPE** | **AUC** |  |
| Inertial sensor data | 0.750-1.00 | 0.750-0.999 | 0.580-1.000 | 0.720-1.000 | [1-6, 10, 12, 13, 16, 17, 19-22, 27-29, 31, 33, 35-40] |
| Pressure sensor data | 0.757-0.990 | 0.725-0.960 | 0.729-0.996 | 0.847 | [7, 15, 24, 25] |
| Optical motion capture sensor data | 0.868 | 0.700-0.821 | 0.780-0.889 | / | [8, 23, 26] |
| Video data | 0.825-0.932 | 0.838-0.888 | 0.823-0.979 | 0.900 | [9, 32] |
| Wireless signal data | 0.980-0.997 | 0.971 | 1.000 | / | [11, 34] |
| **Multimodal research** | 0.821-0.981 | 0.818 | 0.882 | 0.882 | [14, 18, 30] |

1. Yang PK, Filtjens B, Ginis P, Goris M, Nieuwboer A, Gilat M, et al. Freezing of gait assessment with inertial measurement units and deep learning: effect of tasks, medication states, and stops. Journal of neuroengineering and rehabilitation. 2024 Feb 13;21(1):24. PMID: 38350964. doi: 10.1186/s12984-024-01320-1.

2. Yang PK, Filtjens B, Ginis P, Goris M, Nieuwboer A, Gilat M, et al. Automatic Detection and Assessment of Freezing of Gait Manifestations. IEEE transactions on neural systems and rehabilitation engineering : a publication of the IEEE Engineering in Medicine and Biology Society. 2024;32:2699-708. PMID: 39028610. doi: 10.1109/tnsre.2024.3431208.

3. Wang B, Hu X, Ge R, Xu C, Zhang J, Gao Z, et al. Prediction of Freezing of Gait in Parkinson's disease based on multi-channel time-series neural network. Artificial intelligence in medicine. 2024 Aug;154:102932. PMID: 39004005. doi: 10.1016/j.artmed.2024.102932.

4. Sun H, Ye Q, Xia Y. Predicting freezing of gait in patients with Parkinson's disease by combination of Manually-Selected and deep learning features. Biomedical Signal Processing and Control. 2024 Feb;88. PMID: WOS:001092892400001. doi: 10.1016/j.bspc.2023.105639.

5. Sigcha L, Borzi L, Olmo G. Deep learning algorithms for detecting freezing of gait in Parkinson's disease: A cross-dataset study. Expert Systems with Applications. 2024 Dec 1;255. PMID: WOS:001261265000001. doi: 10.1016/j.eswa.2024.124522.

6. Shaban M. A novel variational mode decomposition based convolutional neural network for the identification of freezing of gait intervals for patients with Parkinson's disease. Machine Learning with Applications. 2024 Jun;16. PMID: WOS:001237314800001. doi: 10.1016/j.mlwa.2024.100553.

7. Park JM, Moon CW, Lee BC, Oh E, Lee J, Jang WJ, et al. Detection of freezing of gait in Parkinson's disease from foot-pressure sensing insoles using a temporal convolutional neural network. Frontiers in aging neuroscience. 2024;16:1437707. PMID: 39092074. doi: 10.3389/fnagi.2024.1437707.

8. Park H, Shin S, Youm C, Cheon SM. Deep learning-based detection of affected body parts in Parkinson's disease and freezing of gait using time-series imaging. Scientific reports. 2024 Oct 10;14(1):23732. PMID: 39390087. doi: 10.1038/s41598-024-75445-7.

9. Kondo Y, Bando K, Suzuki I, Miyazaki Y, Nishida D, Hara T, et al. Video-Based Detection of Freezing of Gait in Daily Clinical Practice in Patients With Parkinsonism. IEEE transactions on neural systems and rehabilitation engineering : a publication of the IEEE Engineering in Medicine and Biology Society. 2024;32:2250-60. PMID: 38865235. doi: 10.1109/tnsre.2024.3413055.

10. Huang D, Wu C, Wang Y, Zhang Z, Chen C, Li L, et al. Episode-level prediction of freezing of gait based on wearable inertial signals using a deep neural network model. Biomedical Signal Processing and Control. 2024 Feb;88. PMID: WOS:001102804300001. doi: 10.1016/j.bspc.2023.105613.

11. Habib Z, Mughal MA, Khan MA, Shabaz M. WiFOG: Integrating deep learning and hybrid feature selection for accurate freezing of gait detection. Alexandria Engineering Journal. 2024 Jan;86:481-93. PMID: WOS:001134999900001. doi: 10.1016/j.aej.2023.11.075.

12. Chan LLY, Yang S, Aswani M, Kark L, Henderson E, Lord SR, et al. Development, Validation, and Limits of Freezing of Gait Detection Using a Single Waist-Worn Device. IEEE transactions on bio-medical engineering. 2024 Oct;71(10):3024-31. PMID: 38814761. doi: 10.1109/tbme.2024.3407059.

13. Klaver EC, Heijink IB, Silvestri G, van Vugt JPP, Janssen S, Nonnekes J, et al. Comparison of state-of-the-art deep learning architectures for detection of freezing of gait in Parkinson's disease. Frontiers in neurology. 2023;14:1306129. PMID: 38178885. doi: 10.3389/fneur.2023.1306129.

14. Hu K, Wang Z, Martens KAE, Hagenbuchner M, Bennamoun M, Tsoi AC, et al. Graph Fusion Network-Based Multimodal Learning for Freezing of Gait Detection. IEEE transactions on neural networks and learning systems. 2023 Mar;34(3):1588-600. PMID: 34464270. doi: 10.1109/tnnls.2021.3105602.

15. Hu K, Mei S, Wang W, Martens KAE, Wang L, Lewis SJG, et al. Multi-Level Adversarial Spatio-Temporal Learning for Footstep Pressure Based FoG Detection. IEEE journal of biomedical and health informatics. 2023 Aug;27(8):4166-77. PMID: 37227913. doi: 10.1109/jbhi.2023.3272902.

16. Borzì L, Sigcha L, Rodríguez-Martín D, Olmo G. Real-time detection of freezing of gait in Parkinson's disease using multi-head convolutional neural networks and a single inertial sensor. Artificial intelligence in medicine. 2023 Jan;135:102459. PMID: 36628783. doi: 10.1016/j.artmed.2022.102459.

17. Borzì L, Sigcha L, Olmo G. Context Recognition Algorithms for Energy-Efficient Freezing-of-Gait Detection in Parkinson's Disease. Sensors (Basel, Switzerland). 2023 Apr 30;23(9). PMID: 37177629. doi: 10.3390/s23094426.

18. Bajpai R, Khare S, Joshi D. A Multimodal Model-Fusion Approach for Improved Prediction of Freezing of Gait in Parkinson's Disease. Ieee Sensors Journal. 2023 Jul 15;23(14):16168-75. PMID: WOS:001030784400092. doi: 10.1109/jsen.2023.3284656.

19. Sigcha L, Borzi L, Pavon I, Costa N, Costa S, Arezes P, et al. Improvement of Performance in Freezing of Gait detection in Parkinson's Disease using Transformer networks and a single waist-worn triaxial accelerometer. Engineering Applications of Artificial Intelligence. 2022 Nov;116. PMID: WOS:000869747400005. doi: 10.1016/j.engappai.2022.105482.

20. Shi B, Tay A, Au WL, Tan DML, Chia NSY, Yen SC. Detection of Freezing of Gait Using Convolutional Neural Networks and Data From Lower Limb Motion Sensors. IEEE transactions on bio-medical engineering. 2022 Jul;69(7):2256-67. PMID: 34986092. doi: 10.1109/tbme.2022.3140258.

21. O'Day J, Lee M, Seagers K, Hoffman S, Jih-Schiff A, Kidziński Ł, et al. Assessing inertial measurement unit locations for freezing of gait detection and patient preference. Journal of neuroengineering and rehabilitation. 2022 Feb 13;19(1):20. PMID: 35152881. doi: 10.1186/s12984-022-00992-x.

22. Naghavi N, Wade E. Towards Real-Time Prediction of Freezing of Gait in Patients With Parkinson's Disease: A Novel Deep One-Class Classifier. IEEE journal of biomedical and health informatics. 2022 Apr;26(4):1726-36. PMID: 34375292. doi: 10.1109/jbhi.2021.3103071.

23. Filtjens B, Ginis P, Nieuwboer A, Slaets P, Vanrumste B. Automated freezing of gait assessment with marker-based motion capture and multi-stage spatial-temporal graph convolutional neural networks. Journal of neuroengineering and rehabilitation. 2022 May 21;19(1):48. PMID: 35597950. doi: 10.1186/s12984-022-01025-3.

24. Shalin G, Pardoel S, Lemaire ED, Nantel J, Kofman J. Prediction and detection of freezing of gait in Parkinson's disease from plantar pressure data using long short-term memory neural-networks. Journal of neuroengineering and rehabilitation. 2021 Nov 27;18(1):167. PMID: 34838066. doi: 10.1186/s12984-021-00958-5.

25. Prado A, Kwei SK, Vanegas-Arroyave N, Agrawal SK. Continuous Identification of Freezing of Gait in Parkinson's Patients Using Artificial Neural Networks and Instrumented Shoes. Ieee Transactions on Medical Robotics and Bionics. 2021 Aug;3(3):554-62. PMID: WOS:000896668000003. doi: 10.1109/tmrb.2021.3091526.

26. Filtjens B, Ginis P, Nieuwboer A, Afzal MR, Spildooren J, Vanrumste B, et al. Modelling and identification of characteristic kinematic features preceding freezing of gait with convolutional neural networks and layer-wise relevance propagation. BMC medical informatics and decision making. 2021 Dec 7;21(1):341. PMID: 34876110. doi: 10.1186/s12911-021-01699-0.

27. Esfahani AH, Dyka Z, Ortmann S, Langendoerfer P. Impact of Data Preparation in Freezing of Gait Detection Using Feature-Less Recurrent Neural Network. Ieee Access. 2021 2021;9:138120-31. PMID: WOS:000706816400001. doi: 10.1109/access.2021.3117543.

28. Bikias T, Iakovakis D, Hadjidimitriou S, Charisis V, Hadjileontiadis LJ. DeepFoG: An IMU-Based Detection of Freezing of Gait Episodes in Parkinson's Disease Patients via Deep Learning. Frontiers in robotics and AI. 2021;8:537384. PMID: 34113654. doi: 10.3389/frobt.2021.537384.

29. Sigcha L, Costa N, Pavón I, Costa S, Arezes P, López JM, et al. Deep Learning Approaches for Detecting Freezing of Gait in Parkinson's Disease Patients through On-Body Acceleration Sensors. Sensors (Basel, Switzerland). 2020 Mar 29;20(7). PMID: 32235373. doi: 10.3390/s20071895.

30. Shah SA, Tahir A, Ahmad J, Zahid A, Pervaiz H, Shah SY, et al. Sensor Fusion for Identification of Freezing of Gait Episodes Using Wi-Fi and Radar Imaging. Ieee Sensors Journal. 2020 Dec 1;20(23):14410-22. PMID: WOS:000589257300062. doi: 10.1109/jsen.2020.3004767.

31. Li B, Yao Z, Wang J, Wang S, Yang X, Sun Y. Improved Deep Learning Technique to Detect Freezing of Gait in Parkinson's Disease Based on Wearable Sensors. Electronics. 2020 Nov;9(11). PMID: WOS:000593599400001. doi: 10.3390/electronics9111919.

32. Hu K, Wang Z, Wang W, Martens KAE, Wang L, Tan T, et al. Graph Sequence Recurrent Neural Network for Vision-based Freezing of Gait Detection. IEEE transactions on image processing : a publication of the IEEE Signal Processing Society. 2019 Oct 15. PMID: 31634131. doi: 10.1109/tip.2019.2946469.

33. Ashour AS, El-Attar A, Dey N, Abd El-Kader H, Abd El-Naby MM. Long short term memory based patient-dependent model for FOG detection in Parkinson's disease. Pattern Recognition Letters. 2020 Mar;131:23-9. PMID: WOS:000521971700004. doi: 10.1016/j.patrec.2019.11.036.

34. Tahir A, Ahmad J, Shah SA, Morison G, Skelton DA, Larijani H, et al. WiFreeze: Multiresolution Scalograms for Freezing of Gait Detection in Parkinson's Leveraging 5G Spectrum with Deep Learning. Electronics. 2019 Dec;8(12). PMID: WOS:000506678200060. doi: 10.3390/electronics8121433.

35. San-Segundo R, Navarro-Hellin H, Torres-Sanchez R, Hodgins J, De la Torre F. Increasing Robustness in the Detection of Freezing of Gait in Parkinson's Disease. Electronics. 2019 Feb;8(2). PMID: WOS:000460746500004. doi: 10.3390/electronics8020119.

36. Xia Y, Zhang J, Ye Q, Cheng N, Lu Y, Zhang D. Evaluation of deep convolutional neural networks for detection of freezing of gait in Parkinson's disease patients. Biomedical Signal Processing and Control. 2018 Sep;46:221-30. PMID: WOS:000447109800024. doi: 10.1016/j.bspc.2018.07.015.

37. Kim HB, Lee HJ, Lee WW, Kim SK, Jeon HS, Park HY, et al. Validation of Freezing-of-Gait Monitoring Using Smartphone. Telemedicine and E-Health. 2018 Nov;24(11):899-907. PMID: WOS:000431103100001. doi: 10.1089/tmj.2017.0215.

38. Camps J, Sama A, Martin M, Rodriguez-Martin D, Perez-Lopez C, Moreno Arostegui JM, et al. Deep learning for freezing of gait detection in Parkinson's disease patients in their homes using a waist-worn inertial measurement unit. Knowledge-Based Systems. 2018 Jan 1;139:119-31. PMID: WOS:000417773400011. doi: 10.1016/j.knosys.2017.10.017.

39. Sadiq M, Khan MT, Masood SJC, Materials, Continua. Attention-Based Deep Learning Model for Early Detection of Parkinson’s Disease. 2022;71(3).

40. Al-Nefaie AH, Aldhyani THH, Farhah N, Koundal D. Intelligent diagnosis system based on artificial intelligence models for predicting freezing of gait in Parkinson's disease. Frontiers in medicine. 2024;11:1418684. PMID: 38966531. doi: 10.3389/fmed.2024.1418684.
